# Antitumor effects of iPSC-based cancer vaccine in pancreatic cancer

**DOI:** 10.1016/j.stemcr.2021.04.004

**Published:** 2021-05-06

**Authors:** Xiaoming Ouyang, Yu Liu, Yang Zhou, Jing Guo, Tzu-Tang Wei, Chun Liu, Bomi Lee, Binbin Chen, Angela Zhang, Kerriann M. Casey, Lin Wang, Nigel G. Kooreman, Aida Habtezion, Edgar G. Engleman, Joseph C. Wu

**Affiliations:** 1 Stanford Cardiovascular Institute, Stanford University, Stanford, CA 94305, USA; 2 Department of Medicine, Division of Cardiovascular Medicine, Stanford University, 265 Campus Drive, Stanford, CA 94305, USA; 3 Department of Microbiology and Immunology, Stanford University, Stanford, CA 94305, USA; 4 Department of Medicine, Division of Gastroenterology & Hepatology, Stanford University, Stanford, CA 94305, USA; 5 Department of Genetics, Stanford University, Stanford, CA 94305, USA; 6 Department of Comparative Medicine, Stanford University, Stanford, CA 94305, USA; 7 Department of Pathology, Stanford University, Stanford, CA 94305, USA; 8 Department of Surgery, Leiden University Medical Center, Leiden, ZA 2333, the Netherlands

**Keywords:** iPSC, cancer vaccine, iPSC-based cancer vaccine, pancreatic ductal adenocarcinoma, tumor-associated antigens

## Abstract

Induced pluripotent stem cells (iPSCs) and cancer cells share cellular similarities and transcriptomic profiles. Here, we show that an iPSC-based cancer vaccine, comprised of autologous iPSCs and CpG, stimulated cytotoxic antitumor CD8^+^ T cell effector and memory responses, induced cancer-specific humoral immune responses, reduced immunosuppressive CD4^+^ T regulatory cells, and prevented tumor formation in 75% of pancreatic ductal adenocarcinoma (PDAC) mice. We demonstrate that shared gene expression profiles of “iPSC-cancer signature genes” and others are overexpressed in mouse and human iPSC lines, PDAC cells, and multiple human solid tumor types compared with normal tissues. These results support further studies of iPSC vaccination in PDAC in preclinical and clinical models and in other cancer types that have low mutational burdens.

## Highlights

• The iPSC-based cancer vaccine prevents tumor growth in pancreatic cancer
• The iPSC-based cancer vaccine induces cytotoxic antitumor T cell and B cell responses
• The iPSC-based cancer vaccine reduces immune-suppressive Treg cells
• iPSC-cancer signature genes are upregulated in mouse PDAC and human tumors

## Graphical abstract

**Figure:**
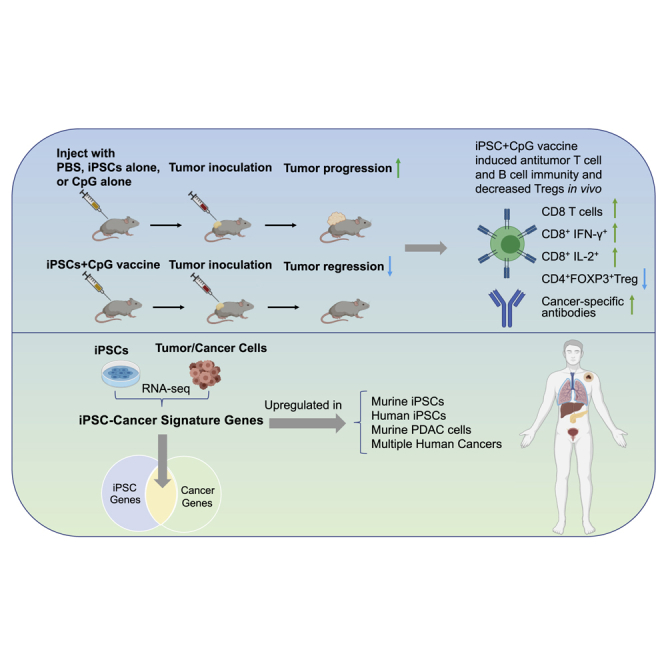

## Teaser

In this article, Wu and colleagues demonstrate that an iPSC-based cancer vaccine, comprised of iPSCs and CpG, stimulated cytotoxic T cell and B cell responses, reduced immune-suppressive Treg cells, and prevented tumor formation in mice injected with pancreatic ductal adenocarcinoma (PDAC) cells. The “iPSC-cancer signature genes” are overexpressed among iPSC lines, PDAC cells, and multiple human cancers. These results support further studies of iPSC-based cancer vaccine for treatment of PDAC.

## Introduction

Pancreatic ductal adenocarcinoma (PDAC) is the fourth leading cause of cancer-related deaths in the USA (Siegel et al., 2019). The 5-year survival rate has remained in the single digits for the last several decades. So far, surgery remains the most effective treatment for this disease; however, only around 10% of patients are diagnosed at a sufficiently early stage when surgical removal of the tumor is possible. Despite the recent success of immune checkpoint inhibitors, PDAC remains mostly resistant to these agents and hence a particularly difficult cancer to treat due to its desmoplastic stroma, the paucity of effector T cells (Torphy et al., 2018), and low mutational burden (Yarchoan et al., 2017). Here, we explored the potential of using non-mutated tumor-associated proteins in induced pluripotent stem cells (iPSCs) as the basis of a PDAC vaccine.

The adaptive immune system can recognize and respond to non-mutated tumor-associated antigens (TAAs) (Ilyas and Yang, 2015). The Food and Drug Administration-approved therapeutic cancer vaccine, Sipuleucel-T (Provenge), was developed as a TAA-based cancer vaccine (Cheever and Higano, 2011). Recently, we reported that induced pluripotent stem cells (iPSCs) share gene expression profiles with cancer cells (Kooreman et al., 2018; Ouyang et al., 2019; Wang et al., 2019). Cluster analysis of RNA sequencing (RNA-seq) data of iPSC lines and cancer cell lines revealed upregulated genes that are shared by both (Kooreman et al., 2018). These genes, which we call iPSC-cancer signature genes, are highly expressed by pluripotent populations but only marginally or not at all by the somatic tissues. We further showed that an iPSC-based cancer vaccine induces iPSC-specific antitumor T cell responses in mice (Kooreman et al., 2018). These findings suggest that the shared proteins between iPSCs and cancer cells contain non-mutant TAAs that can induce antitumor immunity. However, whether an iPSC-based cancer vaccine can induce effective antitumor immunity in tumors, such as PDAC, which have low mutational burdens, is unknown.

In this study, we showed that an iPSC-based cancer vaccine induces protective immunity in a mouse model of PDAC, and that such immunity is associated with an increase in antitumor CD8^+^ effector and memory T cell responses, an induction in cancer cell-specific antibody responses, and a decrease in immunosuppressive CD4^+^ T regulatory cells (Tregs). We further demonstrated that the iPSC-cancer signature genes are commonly overexpressed in mouse and human tumors more than normal tissues in multiple cancer types.

## Results

**Figure 1:**
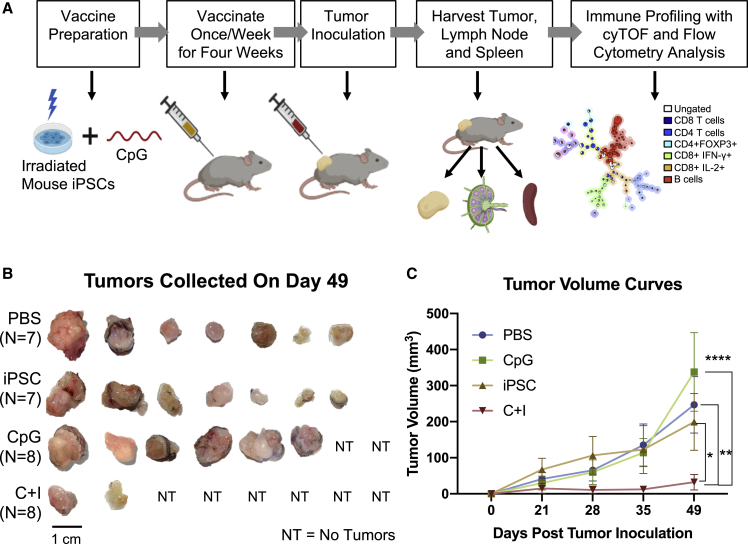
A murine iPSC vaccine prevents tumor formation *in vivo* (A) Diagram showing vaccine preparation consists of sorting murine iPSCs for pluripotency, irradiation, resuspension in adjuvant solution, and subcutaneous injection in the flank. Mice were randomized into different treatment groups and were vaccinated with the C + I vaccine, irradiated iPSCs alone, CpG alone, or PBS for 4 weeks. (B) Vaccination of mice with the C + I vaccine resulted in a complete rejection of the cancer cells in six out of eight mice by day 49 and overall reductions in tumor size (n = 7–8 per group; representative images). (C) Quantification of the tumor volume over time, with values being expressed as means ± SEM (n = 7–8, ^∗^p < 0.05, ^∗∗^p < 0.001, ^∗∗∗∗^p < 0.0001, Tukey's multiple comparison test). Experiments were repeated three times to a total number of seven to eight mice per group.

To evaluate the antitumor effects of the iPSC-based cancer vaccine in PDAC, we generated a mouse iPSC-based vaccine and tested its efficacy in a syngeneic murine PDAC model. The iPSC vaccine consisted of gamma-irradiated autologous iPSCs and an immune adjuvant (synthetic oligodeoxynucleotide [ODN] containing unmethylated CpG motifs) that promotes antigen-presenting cell (APC) maturation (Ballas et al., 2001). Gamma irradiation was needed to prevent teratoma formation by the iPSCs. Mice were injected subcutaneously with (1) phosphate-buffered saline (PBS) control, (2) CpG alone, (3) iPSCs alone, or (4) the combination of CpG + iPSCs (C + I) once a week for 4 weeks (n = 7–8/group), and then inoculated at a separate site with Pan02, a syngeneic murine PDAC line (Corbett et al., 1984) (Figure 1A). In the C + I group, 75% of the vaccinated mice (6/8) completely rejected cancer cells (Figure 1B). In the C + I-vaccinated mice, the mean tumor volume was significantly lower than in mice treated with PBS (p = 0.0050), iPSCs (p = 0.0448), or CpG (p < 0.0001) by day 49 after tumor inoculation (Figure 1C). Interestingly, the CpG alone group had the largest tumor sizes, which was also observed in an orthotopic breast cancer model in a previous study (Kooreman et al., 2018). Histological analysis confirmed the presence of neoplastic cells within the excised tumor from mice treated with PBS, CpG, iPSCs, and the C + I vaccine that developed tumors, and the lack of iPSC-derived teratoma formation in these mice (Figure S1). These results demonstrated the effectiveness and the antitumor effects of the iPSC-based cancer vaccine in PDAC.

**Figure 2:**
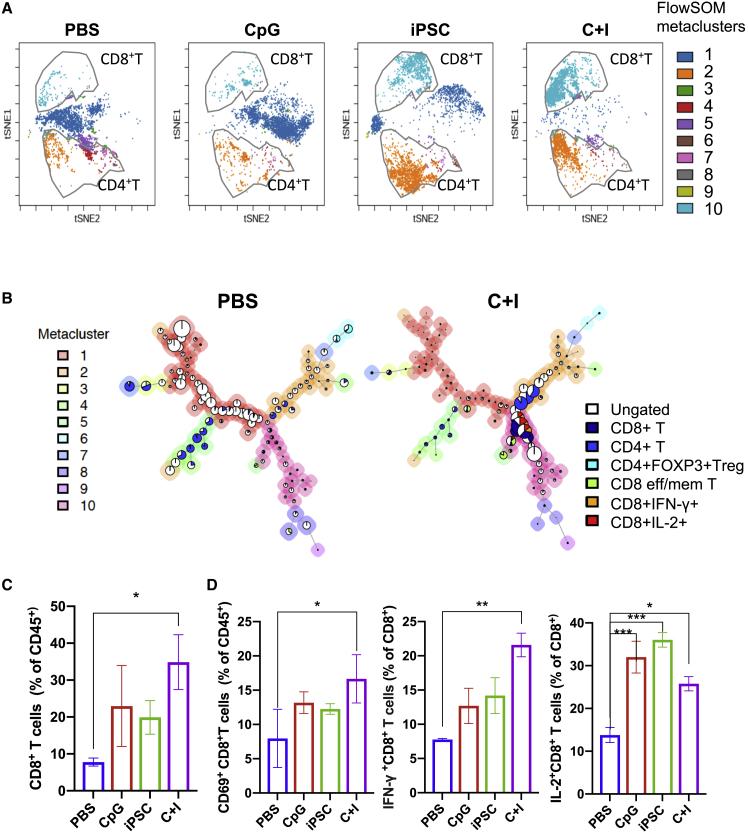
Differences in CD8^+^ T cell activation status and frequency of the T cell subpopulations in TDLNs after iPSC vaccine and control treatments (A) t-Distributed stochastic neighbor embedding (tSNE) visualization of FlowSOM-generated clusters (live CD45^+^ cells) in merged data from cells in draining lymph nodes from mice treated with PBS, CpG alone, iPSCs alone, or the C + I vaccine. CD8^+^ T and CD4^+^ T cells were manually gated and overlaid on tSNE based on marker expression. (B) FlowSOM results from one representative mouse treated with PBS (left panel) or the C + I vaccine (right panel) as minimum spanning trees. FlowSOM was performed using 225 clusters and 10 metaclusters. Each cluster is represented by 1 pie chart, and metaclusters are denoted by background shading. (C) Percentage of CD8^+^ T cells among CD45^+^ cells in TDLNs from mice treated with PBS, CpG, iPSCs, or the C + I vaccine (n = 3, mean ± SEM, ^∗^p < 0.05, compared with PBS, Dunnett's multiple comparison test). (D) Percentage of activated CD69^+^CD8^+^ T cells among CD45^+^ cells in TDLNs from mice treated with PBS, CpG, iPSCs, or the C + I vaccine (left). Percentages of IFN-g^+^CD8^+^ and IL-2^+^CD8^+^ T cells among CD8^+^ T cells in TDLNs from mice treated with PBS, CpG, iPSCs, or the C + I vaccine (middle and right) (n = 3, mean ± SEM, ^∗^p < 0.05, ^∗∗^p < 0.01, ^∗∗∗^p < 0.001 compared with PBS, Dunnett's multiple comparison test).

**Figure 3:**
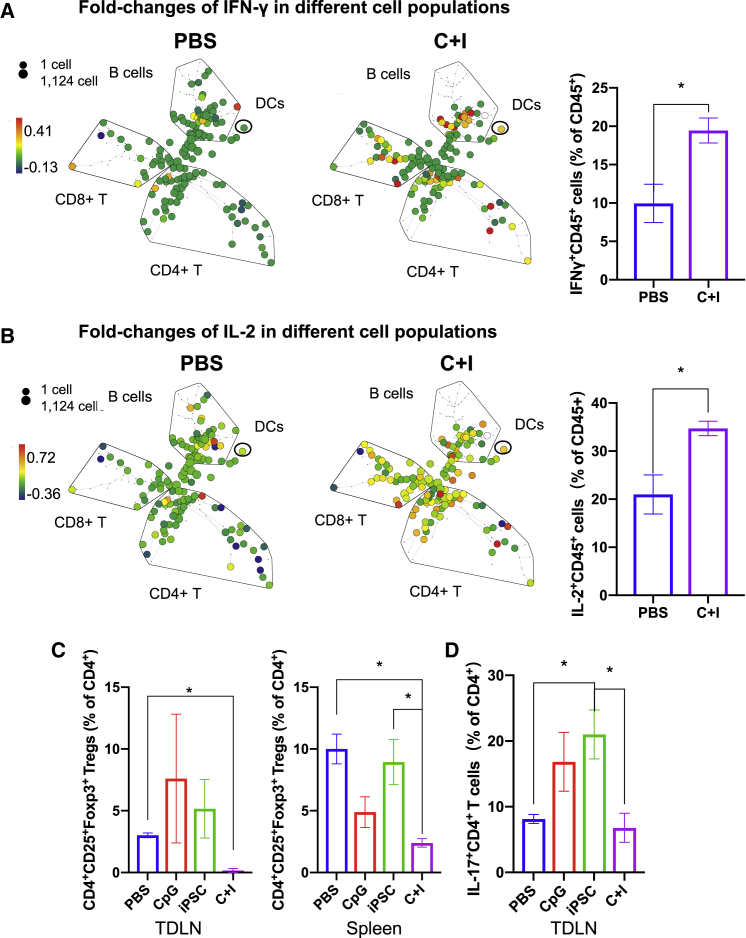
Increased activation in multiple immune cell types and decreased immune suppression after the iPSC vaccine in mice (A and B) Spanning-tree progression analysis of density-normalized events analysis of live CD45^+^ cells in TDLNs from mice treated with PBS and the C + I vaccine with the fold-change intensity of IFN-γ^+^ and IL-2^+^ cells indicated as the color. Frequencies of IFN-γ^+^ and IL-2^+^CD45^+^ cells of total live CD45^+^ cells (n = 3, means ± SEM, ^∗^p < 0.05 compared with PBS, Student's t test). (C) Percentage of CD4^+^CD25^+^FOXP3^+^ Treg cells among CD4^+^ T cells in TDLNs and spleen from mice treated with PBS, CpG, iPSCs, or the C + I vaccine. (D) Percentage of IL-17^+^CD4^+^ T cells among CD4^+^ T cells in TDLNs (right) from mice treated with PBS, CpG, iPSCs, or the C + I vaccine (n = 3–4, mean ± SEM, ^∗^p < 0.05 compared with PBS, Tukey's multiple comparison test).

To define the mechanism underlying the effectiveness of the iPSC vaccine, we next performed immune profiling on vaccinated mice. Because most of the C + I-vaccinated mice did not develop tumors (∼75%), we harvested tumor-draining lymph nodes (TDLNs) from each group and performed cytometry by time-of-flight (CyTOF) analysis to assess potential differences in the frequencies of immune cell populations among the treatment groups. An unsupervised machine learning algorithm, “FlowSOM,” was used for clustering live CD45^+^ cells (Figure S2A) (Gassen et al., 2015). We manually annotated the T cell populations (CD8^+^ T cells, CD4^+^ T cells) based on the median marker intensities in the clusters, and overlaid the cell populations on t-distributed stochastic neighbor-embedding plots for each group (Figure 2A). The results show that C + I immunization significantly increased the frequency of CD8^+^ cytotoxic T cells compared with PBS control. Minimum spanning trees also revealed striking differences in CD8^+^ cytotoxic T cells (metacluster 10 in the pink shade) between the C + I-vaccinated and PBS control groups (Figures 2B, 2C, S2B, and S2C). Of note, large portions of CD8^+^ T cells in the C + I vaccine group, but not in the PBS group, were interferon-γ (IFN-γ) positive and interleukin-2 (IL-2) positive (Figure 2B), indicating enhanced immune activation of these cytotoxic CD8^+^ T cells. We also observed significantly higher frequencies of CD69^+^CD8^+^ T cells and IFN-γ^+^ and IL-2^+^ CD8^+^ T cells in the TDLNs of the C + I vaccine group compared with PBS group (Figure 2D). A fold-change analysis using the spanning-tree progression analysis of density-normalized events showed upregulation of IFN-γ and IL-2 that was induced by the C + I vaccine compared with PBS controls in not only CD8^+^ T cells, but also in CD4^+^ T, B cells, and circulating dendritic cells (DCs) in TDLNs (Figures 3A and 3B). Collectively, these data suggest that the C + I vaccine induced the activation of multiple immune effector cell types in TDLNs.

Tregs can accumulate in the tumor microenvironment to suppress TAA-specific immunity, hence inhibiting antitumor immunity (Bonertz et al., 2009). The FlowSOM MTS revealed a decrease in CD4^+^CD25^+^FOXP3^+^ Tregs, as indicated in cluster 6 (light blue shade), in the C + I vaccine group compared with the PBS group (Figures 2B, S2B, and S2C). We quantified the frequencies of CD4^+^CD25^+^FOXP3^+^ Tregs in all groups and found a significant reduction in Tregs in the C + I vaccine group compared with PBS in both TDLNs and the spleen (Figure 3C), reversing the immune-suppressive microenvironment in mice injected with cancer cells. However, neither the iPSCs alone nor CpG alone treatments reduced the Treg population, and thus both were ineffective in inhibiting tumor growth; in fact, they tended to increase Tregs in TDLNs. These results indicate that the combination of iPSC + CpG exerts a synergistic effect in activating the immune system and inducing antitumor immunity.

Besides Tregs, other tumor-promoting immune cells, such as IL-17-producing-CD4^+^ T cells (T helper 17 [Th17]) (Grivennikov et al., 2012) have also been reported to be a tumor-promoting immune cell type in PDAC murine models (Hegde et al., 2020). In PDAC patients, Th17 are pro-tumor cells and are correlated with poor patient survival (He et al., 2011). In our study, we observed that iPSCs alone increased the frequency of IL-17^+^CD4^+^ Th17 cells in TDLNs compared with PBS control, whereas the C + I vaccine significantly decreased the relative frequency of these cells compared with the iPSCs alone group, and reversed the tumor-promoting immune environment (Figure 3D). Collectively, these data show that the C + I vaccine stimulated anti-cancer cytotoxic T cell responses and suppressed immune-suppressive regulatory T cells.

CpG ODNs have been shown to be able to strongly activate B cells and weakly stimulate plasmacytoid DCs (pDCs) (Krieg et al., 1995; Krug et al., 2001). To determine whether the C + I vaccine induced cancer cell-specific antibodies, we performed serum immunoglobulin G (IgG) binding assay to determine the iPSC- and cancer cell-specific serum IgG levels in PBS and C + I vaccine-treated mice. We found significant increases in iPSC- and cancer cell-specific serum IgG levels, but no significant changes in non-specific IgG that bound to mouse fibroblasts (Figure S3A). These data suggest that the C + I vaccine stimulated a humoral immune response against cancer cells.

To determine whether C + I vaccine increased pDC recruitment in TDLN, we evaluated the percentage of pDCs in the TDLN in PBS and C + I-vaccinated mice. We found that there was a trend of induction of pDC recruitment in the TDLN for the C + I-vaccinated mice compared with PBS control mice (Figure S3B). Collectively, these data suggest that the C + I vaccine could activate B cell responses and increase cancer cell-specific antibodies, and potentially also increase recruitment of pDCs in TDLN.

To determine whether the C + I vaccine stimulated cancer cell-specific T cell memory responses in mice, we performed flow cytometry analysis on splenocytes harvested from mice treated with the C + I vaccine or PBS control, and stimulated the splenocytes with PBS or cancer cell lysate from Panc02 cells. CD8^+^ cytotoxic T cells from C + I-vaccinated mice produced more IFN-γ^+^ upon cancer cell lysate stimulation compared with PBS control-treated mice. Without cancer cell lysate stimulation, no significant difference in IFN-γ^+^ production in CD8^+^ T cells in the spleen was observed in C + I-vaccinated mice or PBS control mice (Figure S3C). These data suggest that a cancer cell-specific CD8^+^ cytotoxic T lymphocyte memory was established in C + I vaccine-treated mice.

Importantly, we also found that the C + I vaccine did not induce significant systemic cytokine production without re-stimulation in peripheral organs, such as the spleen (Figure S3D), nor did it affect the overall appearance or body weights of the mice (Figure S3E), suggesting that the C + I vaccine treatment did not cause significant systemic toxicity and was well tolerated by the mice.

To investigate whether the iPSC vaccine has the potential to provide TAAs specific for PDAC, we first compared the transcriptomics of mouse and human iPSCs and PDAC cancer cells. We evaluated whether there are shared upregulated genes between mouse and human iPSCs with mouse and human pancreatic cancer lines. We performed RNA-seq analysis on mouse and human iPSCs, mouse and human pancreatic cancer lines, and fibroblasts lines. We found shared upregulated genes between mouse/human iPSCs and mouse/human pancreatic cancer lines that are only minimally expressed by mouse or human fibroblasts (Figure S4A).

To extend our study in more cancer types, we investigated the possibility of shared gene expression signatures that are highly expressed by human iPSC lines and multiple cancer lines, but not in normal cell lines. We previously found that human and mouse iPSC lines share their gene expression profiles with those of human and mouse cancer cell lines from multiple cancer types (Kooreman et al., 2018). This analysis revealed 111 upregulated tumor-associated genes that are shared by iPSCs and cancer cells (Table S1). These 111 genes, which we call iPSC-cancer signature genes in this study, are highly expressed by pluripotent populations but only marginally or not at all by the somatic tissues (Kooreman et al., 2018).

To first determine whether the iPSC-cancer signature genes are enriched in mouse PDAC cells and iPSCs that we used in our mouse model, we performed gene set enrichment analysis using RNA-seq data on Panc02 cells and mouse iPSCs, and used the iPSC-cancer signature genes as a user-defined gene set. We found that the expression of the iPSC-cancer signature gene set is enriched in mouse PDAC cell line Panc02 cells and mouse iPSCs compared with mouse embryonic fibroblasts (Figures S4B and S4C).

**Figure 4:**
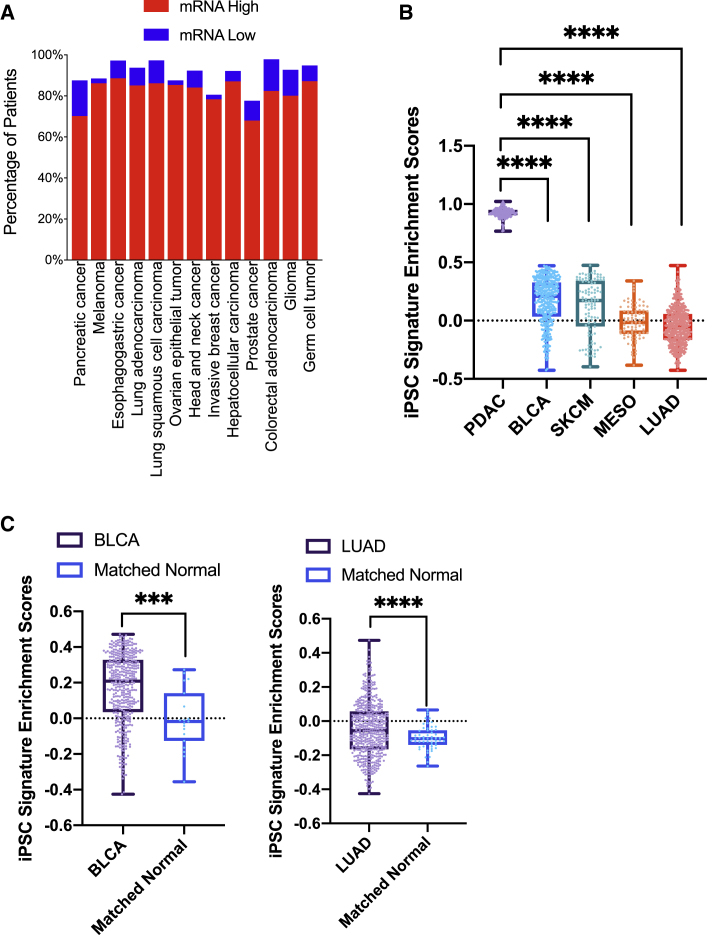
iPSC-cancer signature genes are upregulated in human tumors in TCGA cohorts (A) Analysis of 111 iPSC-cancer signature gene mRNA expression in major human cancer types in TCGA cohorts (*Z* scores > 2 or *Z* scores < −2). (B) Gene enrichment scores of iPSC-cancer signature genes in five cancer types. SKCM, primary skin cutaneous melanoma. PDAC, pancreatic ductal adenocarcinoma. BLCA, bladder urothelial carcinoma. LUAD, lung adenocarcinoma. MESO, mesothelioma (^∗∗∗∗^P < 0.0001 compared with PDAC, Tukey’s multiple comparison test). (C) Enrichment scores of iPSC-cancer signature genes in tumors and matched normal tissues in BLCA and LUAD (^∗∗∗^P < 0.001, ^∗∗∗∗^P < 0.0001, Student’s t test).

To further investigate whether the iPSC-cancer signature is elevated in multiple human cancer types, we examined the expression levels of these genes in human tumors in The Cancer Genome Atlas (TCGA) database. An evaluation of the mRNA expression levels of the iPSC-cancer signature genes in human solid tumors in TCGA PanCancer Atlas Studies (Hoadley et al., 2018; Witkiewicz et al., 2015) revealed high levels of mRNA upregulation in patients' tumors, ranging from 68.1% to 88.7% (Figure 4A), and smaller proportions of mRNA downregulation (compared with all samples that are diploid for that gene in the signature, *Z* score > 2). These data suggest that overexpression of iPSC-cancer signature genes is common in human solid tumors, confirming the resemblance of human tumor cells to iPSCs.

To further investigate the enrichment status of the iPSC-cancer shared genes as a signature, we computed the gene set enrichment scores for the 111 genes as the iPSC-cancer signature genes in five different cancer types in TCGA PanCancer Atlas patients, including pancreatic ductal adenocarcinoma (PDAC), lung adenocarcinoma (LUAD), skin cutaneous melanoma (SKCM), bladder carcinoma (BLCA), and mesothelioma (MESO). We found that the iPSC-cancer signature genes are positively enriched in all PDAC tumors, in the majority of BLCA and primary SKCM tumors, in half of the MESO tumors, and in more than a quarter of LUAD patients (Figure 4B). Notably, in cancer types with sufficient matched adjacent normal samples, such as BLCA and LUAD, the tumor samples have significantly higher enrichment scores than their matched normal counterparts (Figure 4C), suggesting possible roles of these genes in tumor development. Importantly, PDAC tumors not only all have positive enrichment scores, but also have the highest enrichment scores in these genes compared with other cancer types (Figure 4B), indicating that these genes may be particularly important in PDAC.

To determine whether the iPSC-cancer signature genes play direct roles in regulating the immune cells, we performed pathway enrichment analysis of the iPSC-cancer signature genes using the Reactome database, and found that ten of the iPSC-cancer signature genes are involved in immune system-related pathways (Table S2).

As the C + I vaccine stimulated antitumor T cell immune responses, we next sought to identify potential peptide vaccine antigens in the iPSC-cancer signature genes that can be presented to T cells. We scanned 111 iPSC-cancer signature genes with machine learning algorithms, NetMHCpan4.0 (Jurtz et al., 2017) and MARIA (Chen et al., 2019), to identify T cell epitopes highly presentable by human major histocompatibility complex class I (MHC I) and MHC II. Both algorithms are trained not only on binding affinities but also on naturally presented peptides (ligands), taking the antigen presentation process information into account. Optimal vaccine antigens should be presented by multiple MHC I and MHC II alleles in a general population. We ranked candidate T cell epitopes by binding affinity and selected epitopes that are presentable by more than 50% of inputted common MHC I alleles and 100% of inputted common MHC II alleles among the US population. We identified 11 genes that contain T cell epitopes that are highly presentable to both MHC I and MHC II alleles (Table S3). To validate our prediction, we then assessed whether any candidate has been previously demonstrated as a T cell epitope by searching these top candidates with 90% sequence similarity in the Immune Epitope Database. We found that 8 out of the 11 genes contain multiple T cell epitopes that have been previously reported to be recognized by T cells with experimental evidence (Table S3).

## Discussion

In this study, we deployed an iPSC-based cancer vaccine together with an immune adjuvant to target TAAs in PDAC. We evaluated the efficacy of an iPSC-based cancer vaccine and assessed the immune-stimulatory effects of this cancer vaccine in a murine PDAC model. We found that the iPSC-cancer vaccine completely prevented tumor development in 75% of the mice and stimulated antitumor T cell and B cell responses. The iPSC vaccine significantly increased CD8^+^ cytotoxic T cell frequency in the tumor TDLN and promoted T cell activation and antitumor cytokine production (IFN-γ and IL-2) in T lymphocytes, and increased cancer cell-specific antibodies in B cells. Our results demonstrated the effectiveness and the immune-stimulatory effects of this iPSC-based cancer vaccine in PDAC.

The ineffectiveness of current immune checkpoint inhibitors in PDAC may be due to the non-immunogenic and low antigenic nature of these tumors. A clue to the immunogenicity and antigenicity of tumors is tumor mutational load, which has been considered as a response predictor for immune checkpoint inhibitors (Goodman et al., 2017). Studies indicate that a high tumor mutational burden increases the chance that T cells respond and expand to immunogenic neoantigens on tumor cells. Thus, immune checkpoint inhibitors are more effective in tumors with high tumor mutational burden. However, PDAC has a relatively lower somatic mutational burden compared with other immune checkpoint inhibitor-responsive cancers (Chalmers et al., 2017). Interestingly, PDAC tumors are highly enriched in iPSC-cancer signature genes. This suggests that these iPSC genes may have critical roles in the development and progression of PDAC.

The immune system can identify cancer cells by recognizing non-mutated TAAs. Cancer vaccines based on TAAs, such as prostatic acid phosphatase, NY-ESO-1 (cancer/testis antigen), MAGE-A3, and glypican 3, were shown to be immunogenic and induced clinical responses in a subset of patients (Atanackovic et al., 2008; Cheever and Higano, 2011; Vansteenkiste et al., 2016). However, some normal tissues also express low levels of TAAs during development; therefore, the immune system is programmed to develop tolerance toward these antigens by upregulating the immune-suppressive mechanisms, such as Tregs. Treg-mediated suppression of TAA-reactive T cells has been proposed as a potential mechanism for the failure of some TAA-based cancer vaccines (Sakaguchi, 2005).

Our data show that the iPSC-cancer vaccine significantly decreased the CD4^+^CD25^+^FOXP3^+^ Treg population in TDLN and spleen, reversing the immune-suppressive microenvironment in mice injected with cancer cells. However, neither the iPSCs alone nor CpG alone treatments reduced the Treg population and thus both were ineffective in inhibiting tumor growth. These results indicate that the combination of iPSC + CpG exerts a synergistic effect in activating the immune system and inducing antitumor immunity. These data also suggest that the TAA-based iPSC vaccine is a potentially effective anti-cancer strategy suitable for PDAC, which has a low mutational burden and is currently non-responsive to immune checkpoint inhibitors in patients.

Our data show that iPSCs alone did not significantly prevent PDAC formation in mice, and that the addition of CpG is required to achieve the tumor preventive effects. CpGs are unmethylated synthetic oligonucleotides (ODNs) that mimic microbial DNA and thus can activate the Toll-like receptor 9 (TLR9) on APCs, including macrophages, DCs, and B cells (Krieg, 2007). CpG has been investigated in cancer vaccines as an immune adjuvant, as it can improve the function of professional APCs and boost the generation of cellular and humoral vaccine antigen-specific immune responses. Our data show that the induction of both cancer cell-specific T and B cell immune responses in the CpG + iPSCs group compared with the PBS control group, confirming the need of including CpG in the iPSC vaccine.

In this study, we report the iPSC-cancer gene signature consisting of 111 cancer-associated genes that is also highly expressed in iPSCs. However, none of these genes has a well-known anti-cancer immunity-related function. Comparison analysis of transcriptomes of cancer cells of different types, iPSCs clones, and multiple normal tissues using large datasets to identify genes that are only expressed in iPSCs and cancer cells, but not in normal tissues, is still needed. Peptide antigens in iPSCs that can induce the tumor preventive effects and anti-cancer immune responses are under investigation.

The concept of using embryonic stem cells (ESCs) or iPSCs as a prophylactic cancer vaccine has been evaluated in multiple studies in different murine tumor models, including models for lung cancer (Dong et al., 2010; Wang et al., 2020; Yaddanapudi et al., 2012), melanoma (Gąbka-Buszek et al., 2020; Kooreman et al., 2018), ovarian cancer (Zhang et al., 2012), colon cancer (Li et al., 2009), and mesothelioma (Kooreman et al., 2018). These studies show the efficacy of ESC/iPSC-based cancer vaccines in preventing tumor development in mouse models in those cancer types and show the potential of these vaccines as promising prophylactic cancer vaccines, suggesting the need of testing these vaccines in more preclinical models and eventually clinical settings.

Although generating an autologous iPSC line for each patient seems to be less feasible and a prophylactic cancer vaccine seems to be less relevant to clinical medicine at present, under certain scenarios the iPSC-based cancer vaccine described in our study has significant merits as a future immune therapy in clinical settings. Firstly, establishing an autologous iPSC line for every patient is not necessary, as hypoimmunogenic iPSCs can be generated by inactivating MHC I and MHC II genes as a universal iPSC transplantation source for potential clinical use (Deuse et al., 2019). Secondly*,* in a prophylactic setting, the iPSC vaccine can be generated to treat individuals at high risk for developing cancers, such as patients with Lynch syndrome, Li-Fraumeni syndrome, hereditary chronic pancreatitis (Lowenfels et al., 1997; Weiss, 2014), chronic hepatitis B infection (Sherman et al., 1995), or pathogenic germline mutations in BRCA1/2 genes. These patients have a much higher likelihood of developing cancer in their lifetime and thus may be suitable candidates for prophylactic cancer vaccines. Thirdly, the iPSC vaccine can also be used as an adjuvant immunotherapy. We previously showed that, as an adjuvant, the iPSC vaccine inhibited melanoma recurrence at the resection site and reduced metastatic tumor load (Kooreman et al., 2018). The iPSC vaccine could be developed at the time of diagnosis and available at the time of surgical or chemo/radiotherapy treatment of the cancer. Under these scenarios, the clinical development of the iPSC-based cancer vaccine described in our study is warranted and feasible.

In summary, our study demonstrates that the iPSC-based cancer vaccine prevented tumor formation, induced antitumor effector and memory T cell responses and B cell responses, and reduced immune-suppressive Tregs in PDAC, possibly due to synergistic effects of TAAs provided by the iPSCs and APC activation induced by the CpG. We also show the expression and enrichment of iPSC-cancer signature genes in human solid tumors compared with matched adjacent normal tissues, which highlights the clinical relevance of the iPSC-based cancer vaccine in human tumors. Compared with other immunological modalities, iPSC vaccination presents a broad-spectrum of non-mutated tumor antigens to the immune system, potentially making this approach applicable to PDAC and other cancers with low tumor mutational burdens. Further validation of the TAAs in the iPSCs could yield novel peptide-based cancer vaccines suitable for patients with low mutational burden tumors who are non-responsive to immune checkpoint inhibitor or neoantigen vaccine.

## Experimental procedures

### Mouse pancreatic tumor model

Young adult female C57BL/6J (6–8 weeks old) were used. Animals were randomly assigned to different treatment groups (n = 7–8 per group). All experiments were approved by the Stanford University Administrative Panel of Laboratory Animal Care. The murine PDAC cell line Pan02 was a gift from Dr. Aida Habtezion (Stanford University). It was derived from C57BL/6 mice (Corbett et al., 1984). The cancer cells were grown in DMEM and 10% heat-inactivated fetal bovine serum under standard culture conditions. For cancer cell inoculation, 5 × 10^4^ Pan02 cells were resuspended in DMEM without serum and injected subcutaneously in the middle-lower back of the mice. Tumor growth was measured weekly by caliper. Seven weeks after tumor inoculation, mice were euthanized, and tumors, spleens, and TDLNs were harvested for immune profiling.

### iPSC vaccine preparation and immunization

For each mouse, 2 × 10^6^ autologous (C57BL/6J) murine iPSCs were sorted for a pluripotent marker SSEA-1 and were irradiated at 6,000 rads before injection. Irradiated iPSCs were suspended in 5 μM CpG ODN1826 (Invivogen) in PBS and loaded into 28G insulin syringes (Terumo). Mice were anesthetized with 2% isoflurane (Isothesia, Butler Schein) in 100% oxygen until the loss of righting reflex. Immunization was performed by subcutaneous injection of the vaccine in the flanks of the mice, with the injection site alternating every week. Mice were monitored weekly for general health by gross examination of overall appearance and weight measurements.

### Data and code availability

The data that support the findings of this study are available upon request.

## Author contributions

X.O. and J.C.W. conceived the idea and hypothesis. X.O. designed and performed the experiments, data analysis, and wrote the manuscript. Y.L. performed ssGSEA analysis, correlation analysis, and RNA-seq analysis. Y.Z. helped with mouse iPSC irradiation. Y.Z. and L.W. assisted with mouse spleen and lymph node tissue processing. J.G. conjugated anti-FOXP3, anti-Ki67, and anti-CD3 antibodies for cyTOF and provided input on experimental design. T.W. assisted with mouse tissue dissection. C.L. provided technical guidance on mouse iPSC culture. B.L. provided technical guidance on flow cytometry and provided input on experimental design. B.C. and A.Z. provided technical guidance on MARIA. K.M.C. reviewed the histology of the tumors. A.H., N.G.K., E.G.E., and J.C.W. provided critical input on experimental design.

## Conflicts of interests

J.C.W. is the co-founder of Khloris Biosciences but has no competing interests as the work presented here is completely independent.

## Supplemental information

Document S1. Supplemental experimental procedures, Figures S1–S4, and Tables S1–S3

Document S2. Article plus supplemental information

## References

[bib1] Atanackovic D., Altorki N.K., Cao Y., Ritter E., Ferrara C.A., Ritter G., Hoffman E.W., Bokemeyer C., Old L.J., Gnjatic S. (2008). Booster vaccination of cancer patients with MAGE-A3 protein reveals long-term immunological memory or tolerance depending on priming. Proc. Natl. Acad. Sci. U S A.

[bib2] Ballas Z.K., Krieg A.M., Warren T., Rasmussen W., Davis H.L., Waldschmidt M., Weiner G.J. (2001). Divergent therapeutic and immunologic effects of oligodeoxynucleotides with distinct CpG motifs. J. Immunol..

[bib3] Bonertz A., Weitz J., Pietsch D.-H.K., Rahbari N.N., Schlude C., Ge Y., Juenger S., Vlodavsky I., Khazaie K., Jaeger D. (2009). Antigen-specific Tregs control T cell responses against a limited repertoire of tumor antigens in patients with colorectal carcinoma. J. Clin. Invest..

[bib4] Chalmers Z.R., Connelly C.F., Fabrizio D., Gay L., Ali S.M., Ennis R., Schrock A., Campbell B., Shlien A., Chmielecki J. (2017). Analysis of 100,000 human cancer genomes reveals the landscape of tumor mutational burden. Genome Med..

[bib5] Cheever M.A., Higano C.S. (2011). PROVENGE (Sipuleucel-T) in prostate cancer: the first FDA-approved therapeutic cancer vaccine. Clin. Cancer Res..

[bib6] Chen B., Khodadoust M.S., Olsson N., Wagar L.E., Fast E., Liu C.L., Muftuoglu Y., Sworder B.J., Diehn M., Levy R. (2019). Predicting HLA class II antigen presentation through integrated deep learning. Nat. Biotechnol..

[bib7] Corbett T.H., Roberts B.J., Leopold W.R., Peckham J.C., Wilkoff L.J., Griswold D.P., Schabel F.M. (1984). Induction and chemotherapeutic response of two transplantable ductal adenocarcinomas of the pancreas in C57BL/6 mice. Cancer Res..

[bib8] Deuse T., Hu X., Gravina A., Wang D., Tediashvili G., De C., Thayer W.O., Wahl A., Garcia J.V., Reichenspurner H. (2019). Hypoimmunogenic derivatives of induced pluripotent stem cells evade immune rejection in fully immunocompetent allogeneic recipients. Nat. Biotechnol..

[bib42] Dong W., Du J., Shen H., Gao D., Li Z., Wang G., Mu X., Liu Q. (2010). Administration of embryonic stem cells generates effective antitumor immunity in mice with minor and heavy tumor load. Cancer Immunol. Immunother.

[bib45] Gąbka-Buszek A., Kwiatkowska-Borowczyk E., Jankowski J., Kozłowska A.K., Mackiewicz A. (2020). Novel genetic melanoma vaccines based on induced pluripotent stem cells or melanosphere-derived Stem-like cells display high efficacy in a murine tumor rejection model. Vaccines.

[bib10] Gassen S.V., Callebaut B., Helden M.J.V., Lambrecht B.N., Demeester P., Dhaene T., Saeys Y. (2015). FlowSOM: using self-organizing maps for visualization and interpretation of cytometry data. Cytometry A.

[bib11] Goodman A.M., Kato S., Bazhenova L., Patel S.P., Frampton G.M., Miller V., Stephens P.J., Daniels G.A., Kurzrock R. (2017). Tumor mutational burden as an independent predictor of response to immunotherapy in diverse cancers. Mol. Cancer Ther..

[bib12] Grivennikov S.I., Wang K., Mucida D., Stewart C.A., Schnabl B., Jauch D., Taniguchi K., Yu G.-Y., Osterreicher C.H., Hung K.E. (2012). Adenoma-linked barrier defects and microbial products drive IL-23/IL-17-mediated tumour growth. Nature.

[bib13] He S., Fei M., Wu Y., Zheng D., Wan D., Wang L., Li D. (2011). Distribution and clinical significance of Th17 cells in the tumor microenvironment and peripheral blood of pancreatic cancer patients. Int. J. Mol. Sci..

[bib14] Hegde S., Krisnawan V.E., Herzog B.H., Zuo C., Breden M.A., Knolhoff B.L., Hogg G.D., Tang J.P., Baer J.M., Mpoy C. (2020). Dendritic cell paucity leads to dysfunctional immune surveillance in pancreatic cancer. Cancer Cell.

[bib15] Hoadley K.A., Yau C., Hinoue T., Wolf D.M., Lazar A.J., Drill E., Shen R., Taylor A.M., Cherniack A.D., Thorsson V. (2018). Cell-of-origin patterns dominate the molecular classification of 10,000 tumors from 33 types of cancer. Cell.

[bib16] Ilyas S., Yang J.C. (2015). Landscape of tumor antigens in T cell immunotherapy. J. Immunol..

[bib19] Jurtz V., Paul S., Andreatta M., Marcatili P., Peters B., Nielsen M. (2017). NetMHCpan-4.0: improved peptide–MHC class I interaction predictions integrating eluted ligand and peptide binding affinity data. J. Immunol..

[bib20] Kooreman N.G., Kim Y., de Almeida P.E., Termglinchan V., Diecke S., Shao N.-Y., Wei T.-T., Yi H., Dey D., Nelakanti R. (2018). Autologous iPSC-based vaccines elicit anti-tumor responses in vivo. Cell Stem Cell.

[bib21] Krieg A.M. (2007). Antiinfective applications of toll-like receptor 9 agonists. Proc. Am. Thorac. Soc..

[bib22] Krieg A.M., Yi A.K., Matson S., Waldschmidt T.J., Bishop G.A., Teasdale R., Koretzky G.A., Klinman D.M. (1995). CpG motifs in bacterial DNA trigger direct B-cell activation. Nature.

[bib23] Krug A., Rothenfusser S., Hornung V., Jahrsdörfer B., Blackwell S., Ballas Z.K., Endres S., Krieg A.M., Hartmann G. (2001). Identification of CpG oligonucleotide sequences with high induction of IFN-alpha/beta in plasmacytoid dendritic cells. Eur. J. Immunol..

[bib47] Li Y., Zeng H., Xu R.-H., Liu B., Li Z. (2009). Vaccination with human pluripotent stem cells generates a broad spectrum of immunological and clinical responses against colon cancer. Stem Cells.

[bib24] Lowenfels A.B., Maisonneuve P., DiMagno E.P., Elitsur Y., Gates L.K., Perrault J., Whitcomb D.C. (1997). Hereditary pancreatitis and the risk of pancreatic cancer. International Hereditary Pancreatitis Study Group. J. Natl. Cancer Inst..

[bib26] Ouyang X., Telli M.L., Wu J.C. (2019). Induced pluripotent stem cell-based cancer vaccines. Front. Immunol..

[bib27] Sakaguchi S. (2005). Naturally arising Foxp3-expressing CD25+ CD4+ regulatory T cells in immunological tolerance to self and non-self. Nat. Immunol..

[bib28] Sherman M., Peltekian K.M., Lee C. (1995). Screening for hepatocellular carcinoma in chronic carriers of hepatitis B virus: incidence and prevalence of hepatocellular carcinoma in a North American urban population. Hepatology.

[bib30] Siegel R.L., Miller K.D., Jemal A. (2019). Cancer Statistics, 2019. CA Cancer J. Clin..

[bib32] Torphy R.J., Zhu Y., Schulick R.D. (2018). Immunotherapy for pancreatic cancer: barriers and breakthroughs. Ann. Gastroenterol. Surg..

[bib35] Vansteenkiste J.F., Cho B.C., Vanakesa T., De Pas T., Zielinski M., Kim M.S., Jassem J., Yoshimura M., Dahabreh J., Nakayama H. (2016). Efficacy of the MAGE-A3 cancer immunotherapeutic as adjuvant therapy in patients with resected MAGE-A3-positive non-small-cell lung cancer (MAGRIT): a randomised, double-blind, placebo-controlled, phase 3 trial. Lancet Oncol..

[bib36] Wang L., Pegram M.D., Wu J.C. (2019). Induced pluripotent stem cells as a novel cancer vaccine. Expert Opin. Biol. Ther..

[bib43] Wang J., Shao L., Wu L., Ma W., Zheng Y., Hu C., Li F. (2020). Expression levels of a gene signature in hiPSC associated with lung adenocarcinoma stem cells and its capability in eliciting specific antitumor immune-response in a humanized mice model. Thoracic Cancer.

[bib37] Weiss F.U. (2014). Pancreatic cancer risk in hereditary pancreatitis. Front. Physiol..

[bib38] Witkiewicz A.K., McMillan E.A., Balaji U., Baek G., Lin W.-C., Mansour J., Mollaee M., Wagner K.-U., Koduru P., Yopp A. (2015). Whole-exome sequencing of pancreatic cancer defines genetic diversity and therapeutic targets. Nat. Commun..

[bib44] Yaddanapudi K., Mitchell R.A., Putty K., Willer S., Sharma R.K., Yan J., Bodduluri H., Eaton J.W. (2012). Vaccination with Embryonic Stem Cells Protects against Lung Cancer: Is a Broad-Spectrum Prophylactic Vaccine against Cancer Possible?. PLoS One.

[bib39] Yarchoan M., Hopkins A., Jaffee E.M. (2017). Tumor mutational burden and response rate to PD-1 inhibition. N. Engl. J. Med..

[bib46] Zhang Z.-J., Chen X.-H., Chang X.-H., Ye X., Li Y., Cui H. (2012). Human embryonic stem cells--a potential vaccine for ovarian cancer. Asian Pac. J. Cancer Prev..

