# Supplementary material for: Antitumor effects of iPSC-based cancer vaccine in pancreatic cancer
Source: Stem Cell Reports. 2021 May 6;16(6):1468–77. doi: 10.1016/j.stemcr.2021.04.004 (PMC8190592; doi:10.1016/j.stemcr.2021.04.004)
Supplement: Document S1. Supplemental experimental procedures, Figures S1–S4, and Tables S1–S3 [file mmc1.pdf]

Stem Cell Reports, Volume *16*

Supplemental Information

Antitumor effects of iPSC-based cancer vaccine in pancreatic cancer

Xiaoming Ouyang, Yu Liu, Yang Zhou, Jing Guo, Tzu-Tang Wei, Chun Liu, Bomi Lee, Binbin Chen, Angela Zhang, Kerriann M. Casey, Lin Wang, Nigel G. Kooreman, Aida Habtezion, Edgar G. Engleman, and Joseph C. Wu

SUPPLEMENTAL MATERIALS

Antitumor Effects of iPSC-Based Cancer Vaccine in Pancreatic Cancer

Xiaoming Ouyang, PhD[1,2], Yu Liu, PhD[1,2], Yang Zhou, MS[1,2], Jing Guo, PhD[3], Tzu-Tang Wei, PhD[1,2], Chun Liu, PhD[1,2], Bomi Lee, PhD[4], Binbin Chen, PhD[5], Angela Zhang, BS[1,2], Kerriann M. Casey, DVM, DACVP[6], Lin Wang, PhD[1,2], Nigel G. Kooreman, MD, PhD[8], Aida Habtezion, MD, MSc[4], Edgar G. Engleman, MD[7*], Joseph C. Wu, MD, PhD[1,2*]

[1]Stanford Cardiovascular Institute, [2]Department of Medicine, Division of Cardiovascular Medicine, Stanford University, [3]Department of Microbiology and Immunology, [4]Department of Medicine, Division of Gastroenterology & Hepatology, [5]Department of Genetics, [6]Department of Comparative Medicine, [7]Department of Pathology, Stanford University, Stanford, CA 94305, USA, [8]Department of Surgery, Leiden University Medical Center, Leiden, ZA 2333, the Netherlands

Supplemental Experimental Procedures

Generation of murine iPSCs. Murine iPSCs from C57BL/6J mice were generated as previously described (Kooreman et al., 2018). Briefly, fibroblasts from C57BL/6J (The Jackson Laboratory) were transfected with a Neon transfection system (ThermoFisher Scientific) with a codon-optimized mini-intronic plasmid (coMIP) (Diecke et al., 2015). After transfection, cells were cultured on irradiated mouse embryonic feeder cells in DMEM with 15% FBS, MEM Non-Essential Amino Acids (Gibco), and 10 ng/ml murine leukemia inhibiting factor (mLIF; EMD Millipore). Murine iPSC colonies were manually picked and allowed to grow for a few passages, followed by sorting for SSEA-1 using magnetic bead sorting (Miltenyi) to obtain a pure pluripotent population.

Maintenance of murine iPSC line. Murine C57BL/6J iPSC line was maintained in the MEK inhibition (MEKi) and GSK3 inhibition (GSK3i) with leukemia inhibitory factor condition without feeder cells in KnockOut™ DMEM (Gibco) with 15% KnockOut™ Serum Replacement (Gibco), 2 mM GlutaMax, Non-essential Amino Acids (Gibco), 100 µM ß-mercaptoethanol (SigmaAldrich), 0.5 µM ERK-Inhibitor PD0325901 (Selleck Chemicals), 3 µM GSK-3α/ß inhibitor CHIR99021 (Selleck Chemicals), and 10 ng/ml LIF (Millipore) (Silva et al., 2008). Cells were tested for pluripotent marker expression and were tested negative for mycoplasma contamination using the MycoAlert Detection Kit (Lonza). Before use, cells were sorted for SSEA-1 using magnetic bead sorting (Miltenyi) to obtain an enriched pluripotent population.

Histopathology of tumors. The tumors were explanted from mice and processed for histopathology at time of sacrifice. Briefly, the organs were fixed in 4% paraformaldehyde for 72 hr and transferred to 70% ethanol. Fixed samples were embedded in paraffin and sections were cut and stained with hematoxylin and eosin (H&E) for histological analysis by an expert veterinary pathologist (K.M.C).

Cytometry by Time-of-Flight (CyTOF). Immune cells were isolated from tumor draining lymph nodes (TDLNs) and dissociated into a single-cell suspension by pressing the tissue with the plunger of a 3 ml-syringe against a 70 μm strainer. ACK lysing buffer (Quality Biological) was used to remove any red blood cells. Cells were stimulated with PMA/ionomycin for 4 hr together with Brefeldin A. After stimulation, cells were stained with the maxpar mouse spleen/lymph node phenotyping kit (Fluidigm), as well as the maxpar mouse intracellular cytokine I panel kit (Fluidigm) plus anti-mouse FOXP3 and anti-mouse Ki67 antibodies conjugated in-house, and the viability dye Cisplatin (Fluidigm) using the FOXP3/Transcription factor staining buffer set (eBioscience) according to the manufacturer's instructions. Cells were resuspended in MaxPar water at a concentration of $1x10^5$-$1x10^7$ cells/mL with the addition of normalization beads and run on a CyTOF2 (Fluidigm) machine. The resulting data were normalized using the normalization beads. The data were analyzed using Cytobank online software (Kotecha et al., 2010) for viSNE (Amir et al., 2013), FLowSOM (Gassen et al., 2015) and SPADE (Qiu et al., 2011).

IgG binding assay. Cells were washed twice with PBS and resuspended in 100 μl FACS buffer with the addition of 2 μl of serum from the C+I vaccine or PBS treated mice and incubated for

30 min on 4°C. Following incubation, cells were washed twice and incubated with an Anti-IgG (minimal x-reactivity) Goat Polyclonal Antibody (Alexa Fluor® 594) [clone: Poly4053] (BioLegend) for another 20 min on 4°C. Unstained cells were included as negative controls. The cells were then analyzed using the BD FACSymphony™ Flow Cytometer.

Flow cytometry. For the immune profiling of immune cells in the spleen, splenocytes were isolated and incubated with ACK lysing buffer (Quality Biological) to remove red blood cells. Cytokine secretion were blocked for 4 hr by Brefeldin A. Cells were incubated with Panc02 cell lysate or PBS. After incubation, cells were stained with antibodies for surface markers containing CD3 (BioLegend Cat# 100231), CD4 (BioLegend Cat# 116022), CD8a (BioLegend Cat# 100734), CD45 (BioLegend Cat# 103137), and the intracellular markers IL-2 (BD Pharmingen), Granzyme-B (eBioscience), TNF-α (BD Pharmingen), and IFN-γ (Biolegend). A fixable viability dye LIVE/DEAD™ Fixable Near-IR Stain (Thermofisher) was added to exclude dead cells from the analysis. Cells were stained using the FOXP3/Transcription factor staining buffer set (eBioscience) according to the manufacturer's instructions. Samples were analyzed on the LSR-II Flow Cytometer analyzer in the Stanford Shared FACS Facility.

TCGA data sets. Publicly available TCGA patient data sets were used to analyze the percentages of genomic and transcriptomic alterations in cancer-iPSC signature genes in human tumors of major cancer types. The patient cohorts include pancreatic cancer (Hoadley et al., 2018; Witkiewicz et al., 2015), melanoma (Hoadley et al., 2018), esophagogastric cancer (Hoadley et al., 2018), non-small cell lung cancer (Campbell et al., 2016), ovarian epithelial tumor (Hoadley et al., 2018), head and neck cancer (Hoadley et al., 2018), invasive breast

carcinoma (Ciriello et al., 2015), hepatocellular carcinoma (Hoadley et al., 2018), prostate cancer (Armenia et al., 2018), colorectal adenocarcinoma (Hoadley et al., 2018), glioma (Brennan et al., 2013), and germ cell tumor (Bagrodia et al., 2016).

TCGA data analysis using the cBioPortal and R. Publicly available TCGA patient data sets were used to analyze the percentages of transcriptomic alterations in cancer-iPSC signature genes in human tumors of major cancer types. The cBioPortal (http://cbioportal.org) tool (Cerami et al., 2012; Gao et al., 2013) was used for analyzing TCGA data. The patient survival information with or without mRNA overexpression in iPSC-cancer signature genes was queried using cBioportal analysis on TCGA's PanCancer Atlas cohorts. To determine the enrichment scores of the iPSC-cancer signature genes in TCGA tumors, we performed a single-sample Gene Set Enrichment Analysis (ssGSEA) using the GSVA package in R. TCGA RNA sequencing data of tumor samples were downloaded from https://portal.gdc.cancer.gov/.

RNA sequencing data analysis. For RNA-seq data, quality was examined by way of analyzing per base sequence quality plots using FastQC. The trimming of sequence reads was done by TrimGalore. RNA-seq reads were aligned to the mouse genome (mm10) using the STAR software (Dobin et al., 2013). Reads that overlapped with exon coordinates were counted using RSEM and feautrecounts (Li and Dewey, 2011; Liao et al., 2014). Raw read counts were transformed using the variance stabilizing transformation (VST) function included in the DESeq2 R package. Mean and standard deviations of normalized expressions were calculated for each gene. Z-scores were determined by subtracting the mean from each expression value and dividing by the standard deviation. Differentially expressed genes (DEG) between different

groups were identified using DESeq2 R package (Anders and Huber, 2010). Genes with a Benjamin-Hochberg corrected p< 0.05 were considered significant. The RNA-seq expression data sets were downloaded from GEO projects (GEO: GSE157185, GSE160434, GSM4077903 and GSE36294).

Gene Set Enrichment Analysis (GSEA). We utilized the Software "GSEA 4.1.0" (Subramanian et al., 2005) to perform GSEA analysis of iPSC-cancer signature genes as a user-defined gene set with Panc02, mouse iPSCs, and mouse embryonic fibroblasts (MEF) RNA-seq expression data. FDR<0.001 and P < 0.001 was regarded as statistically significant.

Peptide immunogenicity prediction. Immunogenic peptides in iPSC-cancer signature gene were predicted with machine learning algorithms NetMHCpan4.0 and MARIA to identify T cell epitopes highly presentable by HLA alleles (human MHC I and II). For NetMHCpan4.0, we scanned 9-mer peptides in the iPSC-cancer signature genes, and for MARIA we scanned 15-mer peptides. We ranked candidate T cell epitopes by binding affinity and selected epitopes that are presentable by more than 50% of inputted common MHC I alleles and 100% of inputted common MHC II alleles among the US population. We validated the prediction by searching these top candidates with 90% sequence similarity in The Immune Epitope Database (IEDB).

Quantification and statistical analyses. All values in bar graphs and curves are expressed as means ± SEM. Intergroup differences were appropriately assessed by either unpaired two-tailed Student's t-test or one-way analysis of variance (ANOVA) with multiple comparison tests using PRISM GraphPad software. $^{*}P < 0.05$, $^{**}P < 0.01$, $^{***}P < 0.001$, $^{****}P < 0.0001$.

Supplementary Figure Legend

Supplementary Figure 1. H&E staining of mouse tumors confirmed tumor morphology. H&E staining showing histology of mouse tumors from mice treated with PBS, iPSC alone, CpG alone, and the C+I vaccine. NT, no tumors.

Supplementary Figure 2. FlowSOM results for one representative mouse treated with PBS or the C+I vaccine as Minimum Spanning Tree (MST) grid. (**A**) Heatmap representing the expression of the indicated markers within the metaclusters. (**B-C**) FlowSOM was performed using 225 clusters and 10 metaclusters. Each cluster was represented by one pie chart, and metaclusters were denoted by background shading. Major differences observed in metaclusters 10 and 6 between PBS and the C+I vaccine treated mice are outlined in dark blue ($CD8^+$ T cells) and light blue ($CD4^+FOXP3^+$ Treg cells).

Supplementary Figure 3. The C+I vaccine increases serum IgG binding to miPSCs and cancer cells, increases recruitment of plasmocytic dendric cells (pDCs) in tumor draining lymph nodes, and stimulates cancer cell specific IFNγ+ cytotoxic T cells in spleen, but does not increase systemic cytokine levels nor affect mouse body weight production compared to PBS controls. (A) Increased percentage of serum IgG binding to miPSCs and murine PDAC cancer cells from C+I vaccine treated mice compared to PBS treated controls, without a significant increase in non-specific fibroblast binding (*n*=3, mean ± SEM, Student's t-test). **(B)** Percentage of pDCs ($CD45^+CD11c^+NK1.1^-B220^+CD11b^-$) among $CD45^+CD19^-NK1.1^-CD11c^+$ cells in tumor draining lymph nodes from PBS or C+I vaccine treated mice (*n*=3, mean ± SEM, Student's t-test). (**C**) The C+I vaccine stimulates cancer cell specific IFNγ+ cytotoxic T cells in

spleen upon re-exposure to cancer cell lysate compared to PBS controls. Quantification of frequencies of $CD8^+$ IFNγ+ cytotoxic lymphocytes in mouse spleens from C+I vaccine or PBS control treated mice upon exposure to Panc02 lysate or PBS (n=3-4, mean ± SEM, *$P < 0.05$, Student's t-test). **(D)** Quantification of frequencies of $CD3^+$Granzyme B^+, IL-2, or $IFN\gamma^+$ lymphocytes in mouse spleens (n=3-4, means ± SEM, *$P < 0.05$, Tukey's multiple comparisons test). (**E**) No significant difference among different groups in terms of mouse body weights (g) was observed. (n=7-8, mean ± SEM, n.s., not significant, Tukey's multiple comparisons test).

Supplementary Figure 4. Shared upregulated genes in mouse and human iPSCs and pancreatic cancer cell lines. **(A)** Heatmap showing the hierarchical clustering of upregulated genes in mouse iPSC (miPSC.37_rep1, miPSC.37_rep2, miPSC.42_rep1, miPSC.42_rep2), mouse pancreatic cancer cell line Panc02 cells (mPanc02_SY4 and mPanc02_SY13), human iPSC clones (hiPSC_rep1 and hiPSC_rep2), human pancreatic cancer cell lines (hPan_Capan1, hPan_BxPC3, hPan_Aspc1, and hPan_Panc1) compared to mouse embryonic fibroblasts (MEF_rep1 and MEF_rep2) and human adult fibroblasts (hFB_rep1 and hFB_rep1). (**B-C**) Enrichment plots by gene set enrichment analysis (GSEA) for the iPSC cancer gene signature in Panc02 cells and murine iPSCs (miPSCs). iPSC cancer gene signature is significantly enriched in both Panc02 cells and miPSCs, while negatively corelated to mouse embryonic fibroblasts (MEFs). Normalized enrichment scores (NESs), false discovery rate (FDR) q values, and P-values were computed by GSEA.

Supplemental Tables

Supplementary Table 1. iPSC-cancer signature gene table.

Supplementary Table 2. iPSC-cancer signature genes in immune system related pathways.

Supplementary Table 3. Immunogenic iPSC-cancer signature genes and peptides.

References

Amir, E.D., Davis, K.L., Tadmor, M.D., Simonds, E.F., Levine, J.H., Bendall, S.C., Shenfeld, D.K., Krishnaswamy, S., Nolan, G.P., and Pe'er, D. (2013). viSNE enables visualization of high dimensional single-cell data and reveals phenotypic heterogeneity of leukemia. Nat Biotechnol *31*, 545–552.

Anders, S., and Huber, W. (2010). Differential expression analysis for sequence count data. Genome Biology *11*, R106.

Armenia, J., Wankowicz, S.A.M., Liu, D., Gao, J., Kundra, R., Reznik, E., Chatila, W.K., Chakravarty, D., Han, G.C., Coleman, I., et al. (2018). The long tail of oncogenic drivers in prostate cancer. Nat. Genet. *50*, 645–651.

Bagrodia, A., Lee, B.H., Lee, W., Cha, E.K., Sfakianos, J.P., Iyer, G., Pietzak, E.J., Gao, S.P., Zabor, E.C., Ostrovnaya, I., et al. (2016). Genetic determinants of cisplatin resistance in patients with advanced germ cell tumors. J. Clin. Oncol. *34*, 4000–4007.

Brennan, C.W., Verhaak, R.G.W., McKenna, A., Campos, B., Noushmehr, H., Salama, S.R., Zheng, S., Chakravarty, D., Sanborn, J.Z., Berman, S.H., et al. (2013). The somatic genomic landscape of glioblastoma. Cell *155*, 462–477.

Campbell, J.D., Alexandrov, A., Kim, J., Wala, J., Berger, A.H., Pedamallu, C.S., Shukla, S.A., Guo, G., Brooks, A.N., Murray, B.A., et al. (2016). Distinct patterns of somatic genome alterations in lung adenocarcinomas and squamous cell carcinomas. Nat. Genet. *48*, 607–616.

Cerami, E., Gao, J., Dogrusoz, U., Gross, B.E., Sumer, S.O., Aksoy, B.A., Jacobsen, A., Byrne, C.J., Heuer, M.L., Larsson, E., et al. (2012). The cBio cancer genomics portal: an Open platform for exploring multidimensional cancer genomics data. Cancer Discov *2*, 401–404.

Ciriello, G., Gatza, M.L., Beck, A.H., Wilkerson, M.D., Rhie, S.K., Pastore, A., Zhang, H., McLellan, M., Yau, C., Kandoth, C., et al. (2015). Comprehensive molecular portraits of invasive lobular breast cancer. Cell *163*, 506–519.

Diecke, S., Lu, J., Lee, J., Termglinchan, V., Kooreman, N.G., Burridge, P.W., Ebert, A.D., Churko, J.M., Sharma, A., Kay, M.A., et al. (2015). Novel codon-optimized mini-intronic plasmid for efficient, inexpensive, and xeno-free induction of pluripotency. Sci Rep *5*.

Dobin, A., Davis, C.A., Schlesinger, F., Drenkow, J., Zaleski, C., Jha, S., Batut, P., Chaisson, M., and Gingeras, T.R. (2013). STAR: ultrafast universal RNA-seq aligner. Bioinformatics *29*, 15–21.

Gao, J., Aksoy, B.A., Dogrusoz, U., Dresdner, G., Gross, B., Sumer, S.O., Sun, Y., Jacobsen, A., Sinha, R., Larsson, E., et al. (2013). Integrative analysis of complex cancer genomics and clinical profiles using the cBioPortal. Sci Signal *6*, pl1.

Gassen, S.V., Callebaut, B., Helden, M.J.V., Lambrecht, B.N., Demeester, P., Dhaene, T., and Saeys, Y. (2015). FlowSOM: Using self-organizing maps for visualization and interpretation of cytometry data. Cytometry Part A *87*, 636–645.

Hoadley, K.A., Yau, C., Hinoue, T., Wolf, D.M., Lazar, A.J., Drill, E., Shen, R., Taylor, A.M., Cherniack, A.D., Thorsson, V., et al. (2018). Cell-of-origin patterns dominate the molecular classification of 10,000 tumors from 33 types of cancer. Cell *173*, 291-304.e6.

Kooreman, N.G., Kim, Y., de Almeida, P.E., Termglinchan, V., Diecke, S., Shao, N.-Y., Wei, T.-T., Yi, H., Dey, D., Nelakanti, R., et al. (2018). Autologous iPSC-based vaccines elicit anti-tumor responses in vivo. Cell Stem Cell *22*, 501-513.e7.

Kotecha, N., Krutzik, P.O., and Irish, J.M. (2010). Web-based analysis and publication of flow cytometry experiments. Curr Protoc Cytom *Chapter 10*, Unit10.17.

Li, B., and Dewey, C.N. (2011). RSEM: accurate transcript quantification from RNA-Seq data with or without a reference genome. BMC Bioinformatics *12*, 323.

Liao, Y., Smyth, G.K., and Shi, W. (2014). featureCounts: an efficient general purpose program for assigning sequence reads to genomic features. Bioinformatics *30*, 923–930.

Qiu, P., Simonds, E.F., Bendall, S.C., Gibbs, K.D., Bruggner, R.V., Linderman, M.D., Sachs, K., Nolan, G.P., and Plevritis, S.K. (2011). Extracting a cellular hierarchy from high-dimensional cytometry data with SPADE. Nat. Biotechnol. *29*, 886–891.

Silva, J., Barrandon, O., Nichols, J., Kawaguchi, J., Theunissen, T.W., and Smith, A. (2008). Promotion of reprogramming to ground state pluripotency by signal inhibition. PLoS Biol. *6*, e253.

Subramanian, A., Tamayo, P., Mootha, V.K., Mukherjee, S., Ebert, B.L., Gillette, M.A., Paulovich, A., Pomeroy, S.L., Golub, T.R., Lander, E.S., et al. (2005). Gene set enrichment analysis: a knowledge-based approach for interpreting genome-wide expression profiles. Proc Natl Acad Sci U S A *102*, 15545–15550.

Witkiewicz, A.K., McMillan, E.A., Balaji, U., Baek, G., Lin, W.-C., Mansour, J., Mollaee, M., Wagner, K.-U., Koduru, P., Yopp, A., et al. (2015). Whole-exome sequencing of pancreatic cancer defines genetic diversity and therapeutic targets. Nature Communications *6*, 1–11.

Supplementary Figure 1

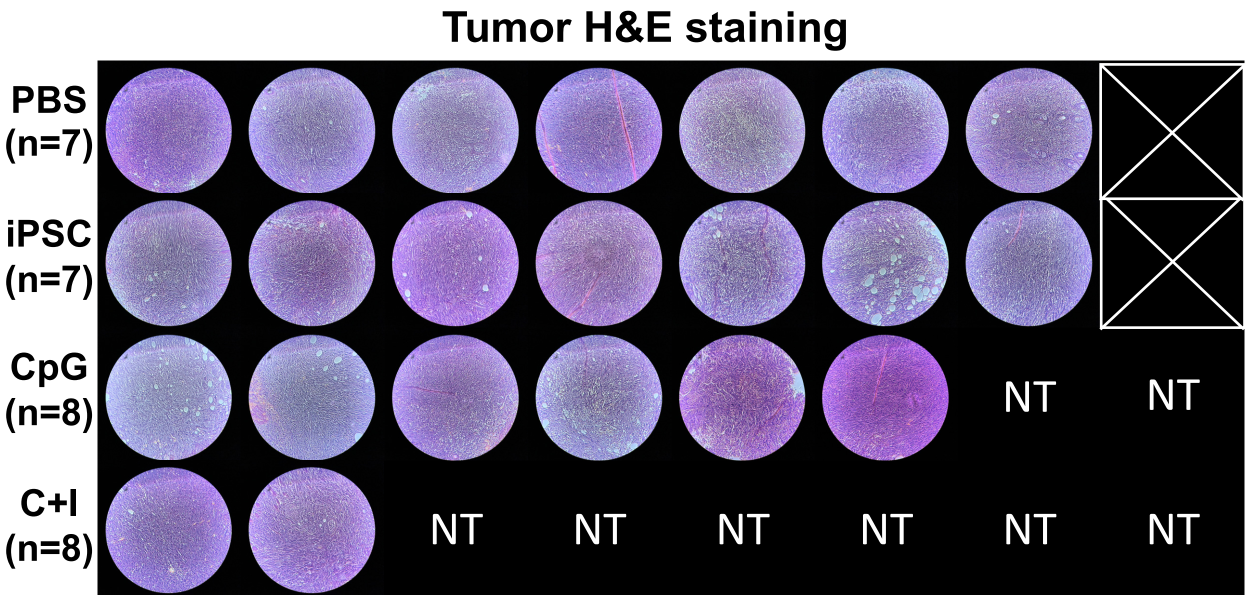

NT , No Tumors.

Supplementary Figure 2

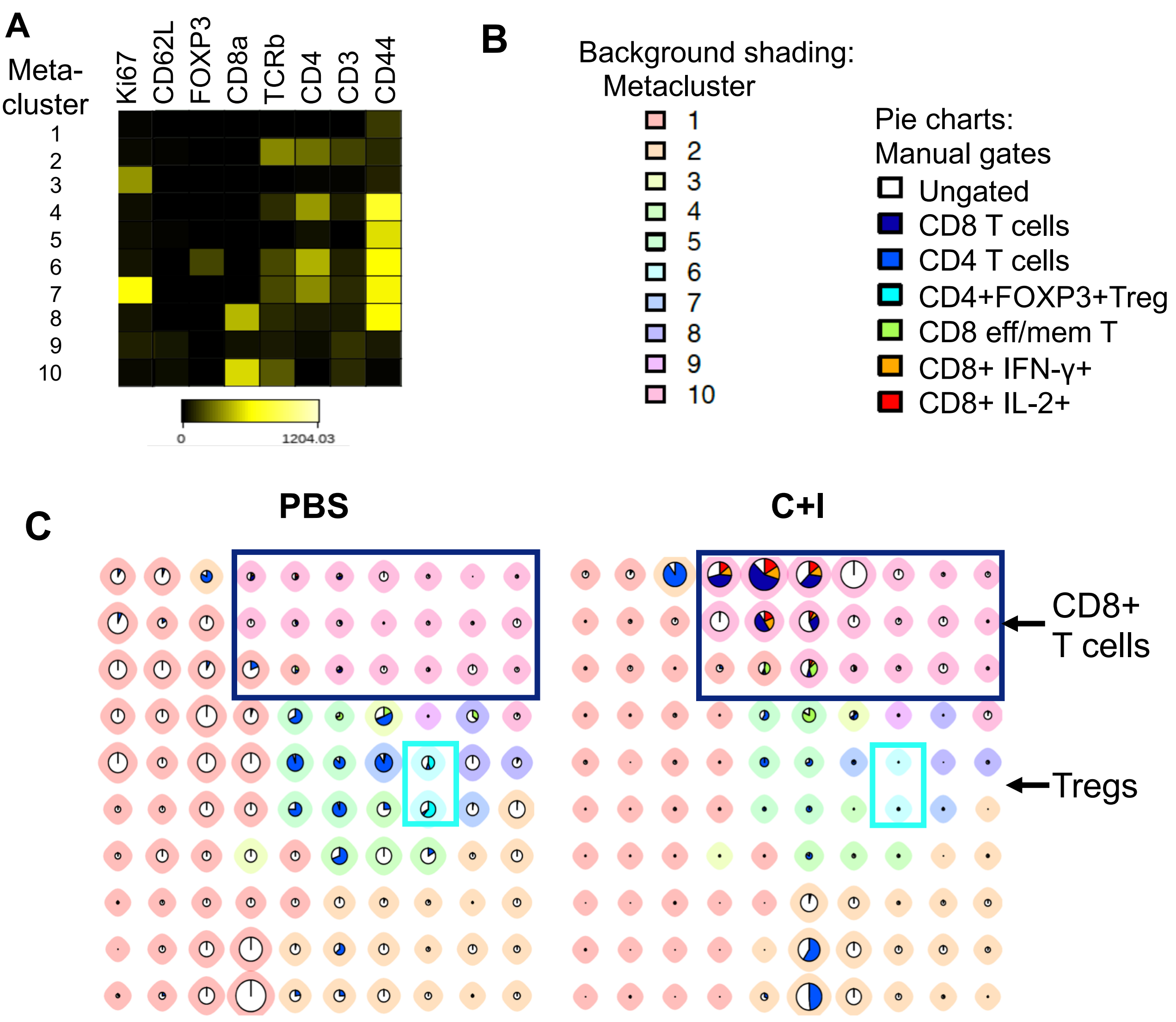

Supplementary Figure 3

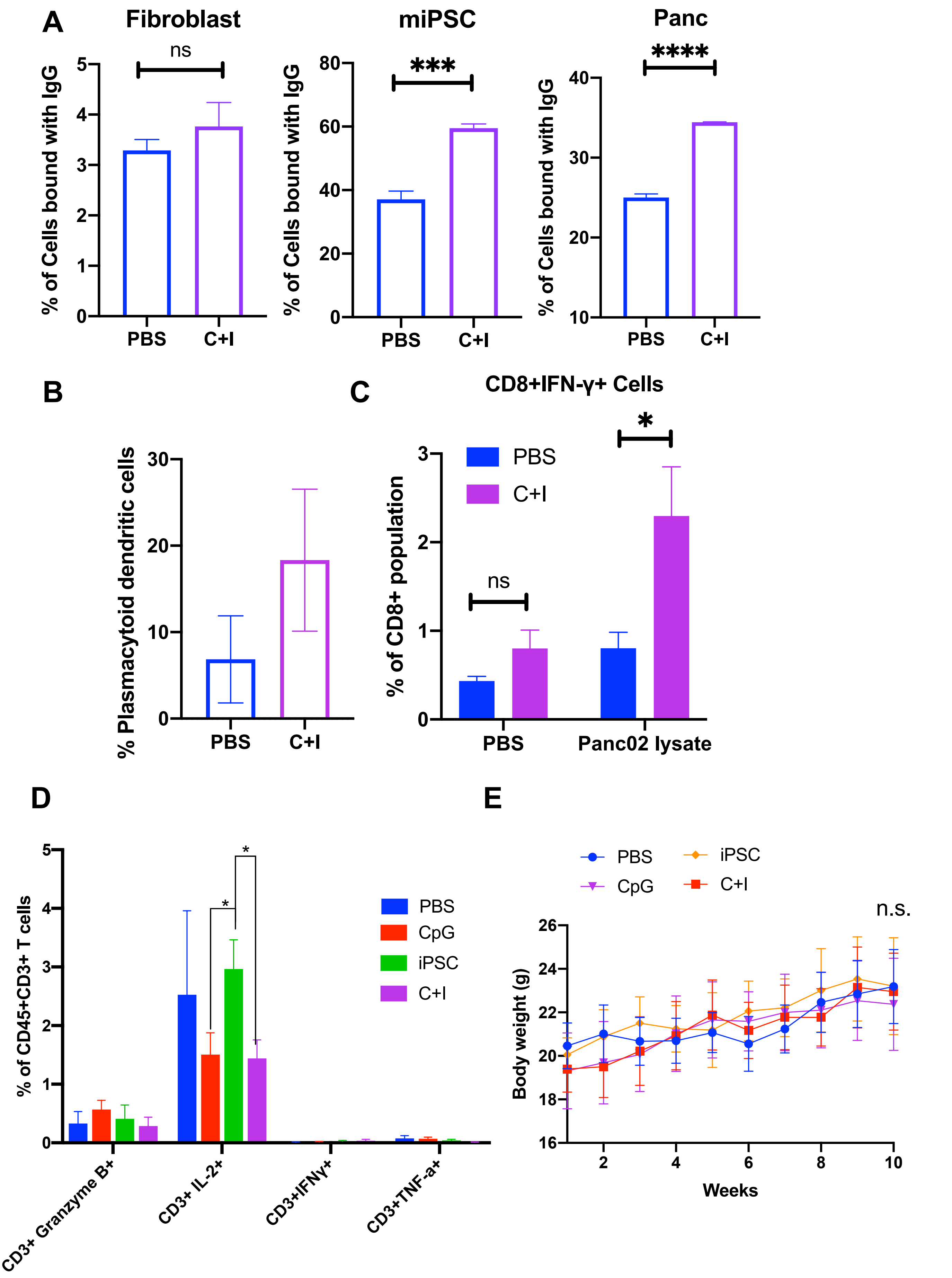

Supplementary Figure 4

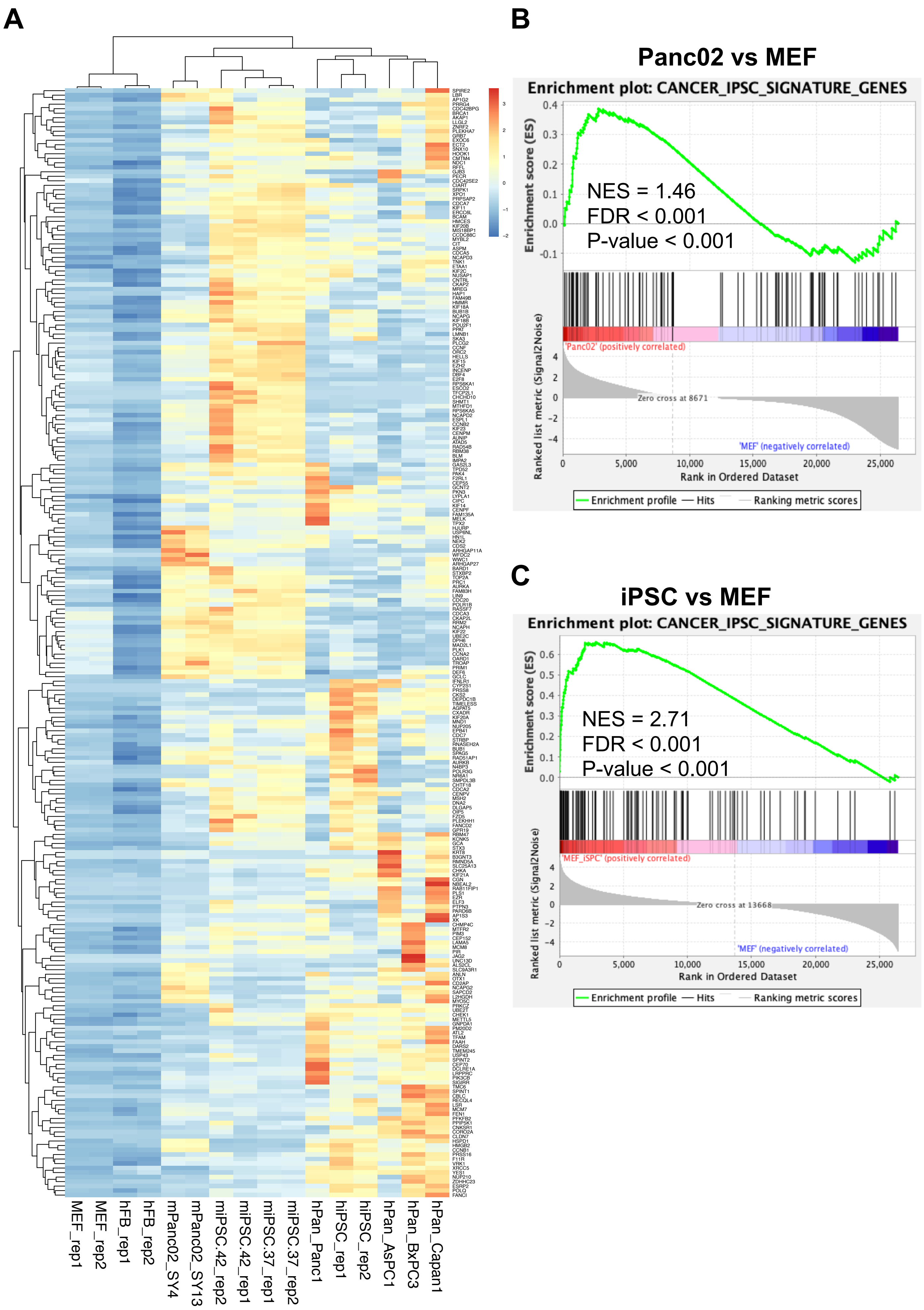

Table S1. iPSC-Cancer Signature Genes

Gene ID	Gene Symbol	Gene Family	Protein Class
HUMAN|HGNC=370|UniProtKB=Q02952	*AKAP12*	A-kinase anchor protein 12;	
HUMAN|HGNC=14082|UniProtKB=Q9NQW6	*ANLN*	Anillin;	
HUMAN|HGNC=11110|UniProtKB=O14497	*ARID1A*	AT-rich interactive domain-containing protein 1A;	
HUMAN|HGNC=19048|UniProtKB=Q8IZT6	*ASPM*	Abnormal spindle-like microcephaly-associated protein;	
HUMAN|HGNC=18318|UniProtKB=Q8IXJ9	*ASXL1*	Putative Polycomb group protein ASXL1;	
HUMAN|HGNC=794|UniProtKB=P31939	*ATIC*	Bifunctional purine biosynthesis protein PURH;	hydrolase(PC00121);methyltransferase(PC00155)
HUMAN|HGNC=11393|UniProtKB=O14965	*AURKA*	Aurora kinase A;	non-receptor serine/threonine protein kinase(PC00167)
HUMAN|HGNC=952|UniProtKB=Q99728	*BARD1*	BRCA1-associated RING domain protein 1;	
HUMAN|HGNC=14347|UniProtKB=Q9H6U6	*BCAS3*	Breast carcinoma-amplified sequence 3;	
HUMAN|HGNC=20893|UniProtKB=Q6W2J9	*BCOR*	BCL-6 corepressor;	
HUMAN|HGNC=1058|UniProtKB=P54132	*BLM*	Bloom syndrome protein;	
HUMAN|HGNC=1100|UniProtKB=P38398	*BRCA1*	Breast cancer type 1 susceptibility protein;	ubiquitin-protein ligase(PC00234)
HUMAN|HGNC=1101|UniProtKB=P51587	*BRCA2*	Breast cancer type 2 susceptibility protein;	damaged DNA-binding protein(PC00086)
HUMAN|HGNC=1103|UniProtKB=P25440	*BRD2*	Bromodomain-containing protein 2;	
HUMAN|HGNC=1104|UniProtKB=Q15059	*BRD3*	Bromodomain-containing protein 3;	
HUMAN|HGNC=13575|UniProtKB=O60885	*BRD4*	Bromodomain-containing protein 4;	
HUMAN|HGNC=20473|UniProtKB=Q9BX63	*BRIP1*	Fanconi anemia group J protein;	DNA helicase(PC00011)
HUMAN|HGNC=1148|UniProtKB=O43683	*BUB1*	Mitotic checkpoint serine/threonine-protein kinase BUB1;	
HUMAN|HGNC=1149|UniProtKB=O60566	*BUB1B*	Mitotic checkpoint serine/threonine-protein kinase BUB1 beta;	
HUMAN|HGNC=1493|UniProtKB=P49589	*CARS*	Cysteine--tRNA ligase, cytoplasmic;	RNA binding protein(PC00031);aminoacyl-tRNA synthetase(PC00047)
HUMAN|HGNC=1578|UniProtKB=P20248	*CCNA2*	Cyclin-A2;	kinase activator(PC00138)
HUMAN|HGNC=1579|UniProtKB=P14635	*CCNB1*	G2/mitotic-specific cyclin-B1;	kinase activator(PC00138)
HUMAN|HGNC=19437|UniProtKB=Q9NPC3	*CCNB1IP1*	E3 ubiquitin-protein ligase CCNB1IP1;	
HUMAN|HGNC=1589|UniProtKB=P24864	*CCNE1*	G1/S-specific cyclin-E1;	kinase activator(PC00138)

HUMAN|HGNC=1591|UniProtKB=P41002	*CCNF*	Cyclin-F;	kinase activator(PC00138)
HUMAN|HGNC=1725|UniProtKB=P30304	*CDC25A*	M-phase inducer phosphatase 1;	protein phosphatase(PC00195)
HUMAN|HGNC=1727|UniProtKB=P30307	*CDC25C*	M-phase inducer phosphatase 3;	protein phosphatase(PC00195)
HUMAN|HGNC=1744|UniProtKB=Q99741	*CDC6*	Cell division control protein 6 homolog;	
HUMAN|HGNC=1722|UniProtKB=P06493	*CDK1*	Cyclin-dependent kinase 1;	non-receptor serine/threonine protein kinase(PC00167);non-receptor tyrosine protein kinase(PC00168)
HUMAN|HGNC=1729|UniProtKB=P21127	*CDK11B*	Cyclin-dependent kinase 11B;	non-receptor serine/threonine protein kinase(PC00167);non-receptor tyrosine protein kinase(PC00168)
HUMAN|HGNC=1772|UniProtKB=Q00526	*CDKN3*	Cyclin-dependent kinase 3;	non-receptor serine/threonine protein kinase(PC00167);non-receptor tyrosine protein kinase(PC00168)
HUMAN|HGNC=1857|UniProtKB=P49454	*CENPF*	Centromere protein F;	
HUMAN|HGNC=1917|UniProtKB=O14647	*CHD2*	Chromodomain-helicase-DNA-binding protein 2;	
HUMAN|HGNC=16627|UniProtKB=O96017	*CHEK2*	Serine/threonine-protein kinase Chk2;	
HUMAN|HGNC=1858|UniProtKB=Q7Z7A1	*CNTRL*	Centriolin;	
HUMAN|HGNC=2348|UniProtKB=Q92793	*CREBBP*	CREB-binding protein;	acetyltransferase(PC00038);chromatin/chromatin-binding protein(PC00077);transcription cofactor(PC00217)
HUMAN|HGNC=18677|UniProtKB=Q9NXZ2	*DDX43*	Probable ATP-dependent RNA helicase DDX43;	
HUMAN|HGNC=3115|UniProtKB=O00716	*E2F3*	Transcription factor E2F3;	nucleic acid binding(PC00171);transcription factor(PC00218)
HUMAN|HGNC=3155|UniProtKB=Q9H8V3	*ECT2*	Protein ECT2;	
HUMAN|HGNC=3279|UniProtKB=Q99613	*EIF3C*	Eukaryotic translation initiation factor 3 subunit C;	translation initiation factor(PC00224)
HUMAN|HGNC=1316|UniProtKB=Q9HC35	*EML4*	Echinoderm microtubule-associated protein-like 4;	
HUMAN|HGNC=3373|UniProtKB=Q09472	*EP300*	Histone acetyltransferase p300;	acetyltransferase(PC00038);chromatin/chromatin-binding protein(PC00077);transcription cofactor(PC00217)
HUMAN|HGNC=11958|UniProtKB=Q96L91	*EP400*	E1A-binding protein p400;	
HUMAN|HGNC=5174|UniProtKB=Q7Z444	*ERAS*	GTPase ERas;	small GTPase(PC00208)
HUMAN|HGNC=27234|UniProtKB=Q86X53	*ERICH1*	Glutamate-rich protein 1;	
HUMAN|HGNC=3494|UniProtKB=P41161	*ETV5*	ETS translocation variant 5;	nucleic acid binding(PC00171);signaling molecule(PC00207);winged helix/forkhead transcription factor(PC00246)

HUMAN|HGNC=3527|UniProtKB=Q15910	*EZH2*	Histone-lysine N-methyltransferase EZH2;	
HUMAN|HGNC=3585|UniProtKB=Q9BXW9	*FANCD2*	Fanconi anemia group D2 protein;	
HUMAN|HGNC=13584|UniProtKB=Q9UKT4	*FBXO5*	F-box only protein 5;	
HUMAN|HGNC=3682|UniProtKB=P08620	*FGF4*	Fibroblast growth factor 4;	growth factor(PC00112)
HUMAN|HGNC=3683|UniProtKB=P12034	*FGF5*	Fibroblast growth factor 5;	growth factor(PC00112)
HUMAN|HGNC=3686|UniProtKB=P55075	*FGF8*	Fibroblast growth factor 8;	growth factor(PC00112)
HUMAN|HGNC=19752|UniProtKB=Q8N3X1	*FNBP4*	Formin-binding protein 4;	
HUMAN|HGNC=3797|UniProtKB=P53539	*FOSB*	Protein fosB;	basic leucine zipper transcription factor(PC00056)
HUMAN|HGNC=3806|UniProtKB=O00358	*FOXE1*	Forkhead box protein E1;	DNA binding protein(PC00009);winged helix/forkhead transcription factor(PC00246)
HUMAN|HGNC=4066|UniProtKB=Q13480	*GAB1*	GRB2-associated-binding protein 1;	transmembrane receptor regulatory/adaptor protein(PC00226)
HUMAN|HGNC=26881|UniProtKB=Q17RS7	*GEN1*	Flap endonuclease GEN homolog 1;	damaged DNA-binding protein(PC00086);endodeoxyribonuclease(PC00093);exodeoxyribonuclease(PC00098);hydrolase(PC00121)
HUMAN|HGNC=5009|UniProtKB=P52926	*HMGA2*	High mobility group protein HMGI-C;	DNA binding protein(PC00009)
HUMAN|HGNC=5125|UniProtKB=P31276	*HOXC13*	Homeobox protein Hox-C13;	
HUMAN|HGNC=5258|UniProtKB=P08238	*HSP90AB1*	Heat shock protein HSP 90-beta;	Hsp90 family chaperone(PC00028)
HUMAN|HGNC=16389|UniProtKB=Q96EW2	*HSPBAP1*	HSPB1-associated protein 1;	
HUMAN|HGNC=17582|UniProtKB=Q8WYB5	*KAT6B*	Histone acetyltransferase KAT6B;	acetyltransferase(PC00038);chromatin/chromatin-binding protein(PC00077);zinc finger transcription factor(PC00244)
HUMAN|HGNC=13610|UniProtKB=Q8NHM5	*KDM2B*	Lysine-specific demethylase 2B;	
HUMAN|HGNC=23025|UniProtKB=Q8TD94	*KLF14*	Krueppel-like factor 14;	DNA binding protein(PC00009);transcription cofactor(PC00217);zinc finger transcription factor(PC00244)
HUMAN|HGNC=6763|UniProtKB=Q13257	*MAD2L1*	Mitotic spindle assembly checkpoint protein MAD2A;	
HUMAN|HGNC=6879|UniProtKB=Q16659	*MAPK6*	Mitogen-activated protein kinase 6;	non-receptor serine/threonine protein kinase(PC00167)
HUMAN|HGNC=6940|UniProtKB=P10911	*MCF2*	Proto-oncogene DBL;	signaling molecule(PC00207)
HUMAN|HGNC=6973|UniProtKB=Q00987	*MDM2*	E3 ubiquitin-protein ligase Mdm2;	chromatin/chromatin-binding protein(PC00077)
HUMAN|HGNC=13363|UniProtKB=Q9H2W2	*MIXL1*	Homeobox protein MIXL1;	

HUMAN|HGNC=7127|UniProtKB=P40692	*MLH1*	DNA mismatch repair protein Mlh1;	DNA binding protein(PC00009)
HUMAN|HGNC=7325|UniProtKB=P43246	*MSH2*	DNA mismatch repair protein Msh2;	DNA binding protein(PC00009)
HUMAN|HGNC=7329|UniProtKB=P52701	*MSH6*	DNA mismatch repair protein Msh6;	DNA binding protein(PC00009)
HUMAN|HGNC=23784|UniProtKB=Q9BTC8	*MTA3*	Metastasis-associated protein MTA3;	chromatin/chromatin-binding protein(PC00077);histone(PC00118)
HUMAN|HGNC=3942|UniProtKB=P42345	*MTOR*	Serine/threonine-protein kinase mTOR;	non-receptor serine/threonine protein kinase(PC00167);nucleic acid binding(PC00171);nucleotide kinase(PC00172)
HUMAN|HGNC=7547|UniProtKB=P10243	*MYBL1*	Myb-related protein A;	
HUMAN|HGNC=7559|UniProtKB=P04198	*MYCN*	N-myc proto-oncogene protein;	basic helix-loop-helix transcription factor(PC00055);nucleic acid binding(PC00171)
HUMAN|HGNC=7670|UniProtKB=Q9Y6Q9	*NCOA3*	Nuclear receptor coactivator 3;	acetyltransferase(PC00038);transcription factor(PC00218)
HUMAN|HGNC=7857|UniProtKB=P30419	*NMT1*	Glycylpeptide N-tetradecanoyltransferase 1;	transferase(PC00220)
HUMAN|HGNC=18016|UniProtKB=Q8WUM0	*NUP133*	Nuclear pore complex protein Nup133;	
HUMAN|HGNC=8522|UniProtKB=P32243	*OTX2*	Homeobox protein OTX2;	homeodomain transcription factor(PC00119)
HUMAN|HGNC=26144|UniProtKB=Q86YC2	*PALB2*	Partner and localizer of BRCA2;	
HUMAN|HGNC=8729|UniProtKB=P12004	*PCNA*	Proliferating cell nuclear antigen;	DNA polymerase processivity factor(PC00015)
HUMAN|HGNC=14005|UniProtKB=Q86TG7	*PEG10*	Retrotransposon-derived protein PEG10;	
HUMAN|HGNC=8987|UniProtKB=Q9P1W9	*PIM2*	Serine/threonine-protein kinase pim-2;	serine/threonine protein kinase receptor(PC00205)
HUMAN|HGNC=9045|UniProtKB=Q6DJT9	*PLAG1*	Zinc finger protein PLAG1;	KRAB box transcription factor(PC00029)
HUMAN|HGNC=9221|UniProtKB=Q01860	*POU5F1*	POU domain, class 5, transcription factor 1;	
HUMAN|HGNC=9347|UniProtKB=Q13029	*PRDM2*	PR domain zinc finger protein 2;	
HUMAN|HGNC=9822|UniProtKB=O15315	*RAD51B*	DNA repair protein RAD51 homolog 2;	
HUMAN|HGNC=14428|UniProtKB=Q9H2T7	*RANBP17*	Ran-binding protein 17;	transfer/carrier protein(PC00219)
HUMAN|HGNC=9965|UniProtKB=Q9P2R6	*RERE*	Arginine-glutamic acid dipeptide repeats protein;	
HUMAN|HGNC=9966|UniProtKB=Q13127	*REST*	RE1-silencing transcription factor;	KRAB box transcription factor(PC00029)
HUMAN|HGNC=10315|UniProtKB=P35268	*RPL22*	60S ribosomal protein L22;	ribosomal protein(PC00202)
HUMAN|HGNC=10451|UniProtKB=P23921	*RRM1*	Ribonucleoside-diphosphate reductase large subunit;	reductase(PC00198)
HUMAN|HGNC=20256|UniProtKB=Q5VUG0	*SFMBT2*	Scm-like with four MBT domains protein 2;	chromatin/chromatin-binding protein(PC00077);transcription factor(PC00218)

HUMAN|HGNC=10901|UniProtKB=Q13309	*SKP2*	S-phase kinase-associated protein 2;	
HUMAN|HGNC=11195|UniProtKB=P48431	*SOX2*	Transcription factor SOX-2;	HMG box transcription factor(PC00024)
HUMAN|HGNC=11199|UniProtKB=P41225	*SOX3*	Transcription factor SOX-3;	HMG box transcription factor(PC00024)
HUMAN|HGNC=10879|UniProtKB=Q15468	*STIL*	SCL-interrupting locus protein;	
HUMAN|HGNC=11524|UniProtKB=Q9Y6A5	*TACC3*	Transforming acidic coiled-coil-containing protein 3;	
HUMAN|HGNC=11648|UniProtKB=P56279	*TCL1A*	T-cell leukemia/lymphoma protein 1A;	
HUMAN|HGNC=11701|UniProtKB=P13385	*TDGF1*	Teratocarcinoma-derived growth factor 1;	calcium-binding protein(PC00060)
HUMAN|HGNC=29484|UniProtKB=Q8NFU7	*TET1*	Methylcytosine dioxygenase TET1;	
HUMAN|HGNC=11752|UniProtKB=P19532	*TFE3*	Transcription factor E3;	
HUMAN|HGNC=11805|UniProtKB=Q13009	*TIAM1*	T-lymphoma invasion and metastasis-inducing protein 1;	
HUMAN|HGNC=11989|UniProtKB=P11388	*TOP2A*	DNA topoisomerase 2-alpha;	
HUMAN|HGNC=7146|UniProtKB=Q7Z4N2	*TRPM1*	Transient receptor potential cation channel subfamily M member 1;	ion channel(PC00133);receptor(PC00197)
HUMAN|HGNC=20071|UniProtKB=Q53GS9	*USP39*	U4/U6.U5 tri-snRNP-associated protein 2;	cysteine protease(PC00081)
HUMAN|HGNC=12718|UniProtKB=Q99986	*VRK1*	Serine/threonine-protein kinase VRK1;	non-receptor serine/threonine protein kinase(PC00167)
HUMAN|HGNC=8014|UniProtKB=P67809	*YBX1*	Nuclease-sensitive element-binding protein 1;	
HUMAN|HGNC=9397|UniProtKB=Q9ULU4	*ZMYND8*	Protein kinase C-binding protein 1;	
HUMAN|HGNC=13099|UniProtKB=P13682	*ZNF35*	Zinc finger protein 35;	KRAB box transcription factor(PC00029)

Table S2. iPSC-Cancer Signature Genes In Immune System Related Pathways

Pathway name	# of genes found	# of total gene in the pathway	Species name	Genes found in pathway
Adaptive Immune System	4	1003	Homo sapiens	ASXL1; CCNF; SKP2; MTOR
Cytokine Signaling in Immune system	3	1108	Homo sapiens	SOX2; NUP133; MTOR
Innate Immune System	4	1331	Homo sapiens	CREBBP; ATIC; HSP90AB1; EP300
Immune System	10	2713	Homo sapiens	SOX2; ASXL1; CREBBP; ATIC; HSP90AB1; NUP133; CCNF; EP300; SKP2; MTOR

Table S3. Immunogenic iPSC-Cancer Signature Genes and Peptides

Gene ID	MHC I Peptide	MHC II Peptide	Position	MHC-I Coverage %	MHC-II Coverage %	IEDB Evidence Number
ASPM	MIIAVTSYK	QSRIRMIIAVTSYKR	1382	55	100	1
	RMHRLHMRY	IQSTFRMHRLHMRYQ	2345	50	100	1
	FAMKVLASY	FRAYIFAMKVLASYQ	1620	50	100	4
	FQVDISLNL	IAFAFQVDISLNLDQ	1054	50	100	0
	LAMFILNRL	GLAMFILNRLLWNPD	846	50	100	0
	RAYKLYLAV	RAYKLYLAVKNANKQ	3125	50	100	0
	FLNVRASAI	RTRFLNVRASAIIIQ	2996	100	100	0
ATIC	KAFTHTAQY	LKAFTHTAQYDEAIS	176	50	100	1
	YTQSNSVCY	TIAVKYTQSNSVCYA	426	100	100	1
BRD4	FAWPFQQPV	HQFAWPFQQPVDAVK	78	100	100	1
CARS1	ALLENIALY	RPNQALLENIALYLT	541	50	100	22
	YVSNGSVYF	DNGYGYVSNGSVYFD	261	100	100	7
CREBBP	QQMRTLNPL	HQQMRTLNPLGNNPM	515	60	100	0
EP300	LMFNNAWLY	DDIWLMFNNAWLYNR	1122	65	100	1
	YLDSVHFFR	VYISYLDSVHFFRPK	1396	50	100	7
	YSYQNRYHF	DATYYSYQNRYHFCE	1191	50	100	3
ERICH1	FSYWITHIL	AFFSYWITHILPEKS	428	50	100	0
MTOR	HPQALIYPL	HPQALIYPLTVASKS	1967	50	100	1
	LEWLRRLSL	KDDWLEWLRRLSLEL	1280	50	100	1
	LQHYVTMEL	ELQHYVTMELREMSQ	43	50	100	4
	MPFLRKMLI	NPAFVMPFLRKMLIQ	726	50	100	2
	YAMKHFGEL	VLEYAMKHFGELEIQ	1434	55	100	0
	YASRIIHPI	SLDFTDYASRIIHPI	1150	60	100	2
NUP133	RERSSFYSL	RERSSFYSLTSSNIS	280	50	100	0
	YSWDINRAL	EKHAYSWDINRALKE	307	100	100	0
PEG10	QTYPTYAAY	IPGYQTYPTYAAYPT	636	55	100	0
	FMMEMKHVF	PAFMMEMKHVFEDPQ	149	65	100	2
	YAAYPTYPV	YPTYAAYPTYPVGFA	641	50	100	5
	YVAQNGIPL	FIDHEYVAQNGIPLR	381	100	100	2
RRM1	IIYDRDFSY	SAIIYDRDFSYNYFG	134	60	100	8
	RVYNNTARY	LRVYNNTARYVDQGG	276	60	100	14
	FQIVNPHLL	SGEFQIVNPHLLKDL	633	50	100	49
