# Supplementary material for: Antitumor effects of iPSC-based cancer vaccine in pancreatic cancer
Source: Stem Cell Reports. 2021 May 6;16(6):1468–77. doi: 10.1016/j.stemcr.2021.04.004 (PMC8190592; doi:10.1016/j.stemcr.2021.04.004)
Supplement: Document S2. Article plus supplemental information [file mmc2.pdf]

Stem Cell Reports

Report

OPEN ACCESS

Antitumor effects of iPSC-based cancer vaccine in pancreatic cancer

Xiaoming Ouyang,[1,2] Yu Liu,[1,2] Yang Zhou,[1,2] Jing Guo,[3] Tzu-Tang Wei,[1,2] Chun Liu,[1,2] Bomi Lee,[4] Binbin Chen,[5] Angela Zhang,[1,2] Kerriann M. Casey,[6] Lin Wang,[1,2] Nigel G. Kooreman,[8] Aida Habtezion,[4] Edgar G. Engleman,[7,*] and Joseph C. Wu[1,2,*]

[1]Stanford Cardiovascular Institute, Stanford University, Stanford, CA 94305, USA
[2]Department of Medicine, Division of Cardiovascular Medicine, Stanford University, 265 Campus Drive, Stanford, CA 94305, USA
[3]Department of Microbiology and Immunology, Stanford University, Stanford, CA 94305, USA
[4]Department of Medicine, Division of Gastroenterology & Hepatology, Stanford University, Stanford, CA 94305, USA
[5]Department of Genetics, Stanford University, Stanford, CA 94305, USA
[6]Department of Comparative Medicine, Stanford University, Stanford, CA 94305, USA
[7]Department of Pathology, Stanford University, Stanford, CA 94305, USA
[8]Department of Surgery, Leiden University Medical Center, Leiden, ZA 2333, the Netherlands
*Correspondence: edgareng@stanford.edu (E.G.E.), joewu@stanford.edu (J.C.W.)
https://doi.org/10.1016/j.stemcr.2021.04.004

SUMMARY

Induced pluripotent stem cells (iPSCs) and cancer cells share cellular similarities and transcriptomic profiles. Here, we show that an iPSC-based cancer vaccine, comprised of autologous iPSCs and CpG, stimulated cytotoxic antitumor CD8^{+} T cell effector and memory responses, induced cancer-specific humoral immune responses, reduced immunosuppressive CD4^{+} T regulatory cells, and prevented tumor formation in 75% of pancreatic ductal adenocarcinoma (PDAC) mice. We demonstrate that shared gene expression profiles of "iPSC-cancer signature genes" and others are overexpressed in mouse and human iPSC lines, PDAC cells, and multiple human solid tumor types compared with normal tissues. These results support further studies of iPSC vaccination in PDAC in preclinical and clinical models and in other cancer types that have low mutational burdens.

INTRODUCTION

Pancreatic ductal adenocarcinoma (PDAC) is the fourth leading cause of cancer-related deaths in the USA (Siegel et al., 2019). The 5-year survival rate has remained in the single digits for the last several decades. So far, surgery remains the most effective treatment for this disease; however, only around 10% of patients are diagnosed at a sufficiently early stage when surgical removal of the tumor is possible. Despite the recent success of immune checkpoint inhibitors, PDAC remains mostly resistant to these agents and hence a particularly difficult cancer to treat due to its desmoplastic stroma, the paucity of effector T cells (Torphy et al., 2018), and low mutational burden (Yarchoan et al., 2017). Here, we explored the potential of using non-mutated tumor-associated proteins in induced pluripotent stem cells (iPSCs) as the basis of a PDAC vaccine.

The adaptive immune system can recognize and respond to non-mutated tumor-associated antigens (TAAs) (Ilyas and Yang, 2015). The Food and Drug Administration-approved therapeutic cancer vaccine, Sipuleucel-T (Provenge), was developed as a TAA-based cancer vaccine (Cheever and Higano, 2011). Recently, we reported that induced pluripotent stem cells (iPSCs) share gene expression profiles with cancer cells (Kooreman et al., 2018; Ouyang et al., 2019; Wang et al., 2019). Cluster analysis of RNA sequencing (RNA-seq) data of iPSC lines and cancer cell lines revealed upregulated genes that are shared by both (Kooreman et al., 2018). These genes, which we call iPSC-cancer signature genes, are highly expressed by pluripotent populations but only marginally or not at all by the somatic tissues. We further showed that an iPSC-based cancer vaccine induces iPSC-specific antitumor T cell responses in mice (Kooreman et al., 2018). These findings suggest that the shared proteins between iPSCs and cancer cells contain non-mutant TAAs that can induce antitumor immunity. However, whether an iPSC-based cancer vaccine can induce effective antitumor immunity in tumors, such as PDAC, which have low mutational burdens, is unknown.

In this study, we showed that an iPSC-based cancer vaccine induces protective immunity in a mouse model of PDAC, and that such immunity is associated with an increase in antitumor CD8^{+} effector and memory T cell responses, an induction in cancer cell-specific antibody responses, and a decrease in immunosuppressive CD4^{+} T regulatory cells (Tregs). We further demonstrated that the iPSC-cancer signature genes are commonly overexpressed in mouse and human tumors more than normal tissues in multiple cancer types.

RESULTS

To evaluate the antitumor effects of the iPSC-based cancer vaccine in PDAC, we generated a mouse iPSC-based vaccine and tested its efficacy in a syngeneic murine PDAC model. The iPSC vaccine consisted of gamma-irradiated autologous iPSCs and an immune adjuvant (synthetic

This is an open access article under the CC BY license (http://creativecommons.org/licenses/by/4.0/).

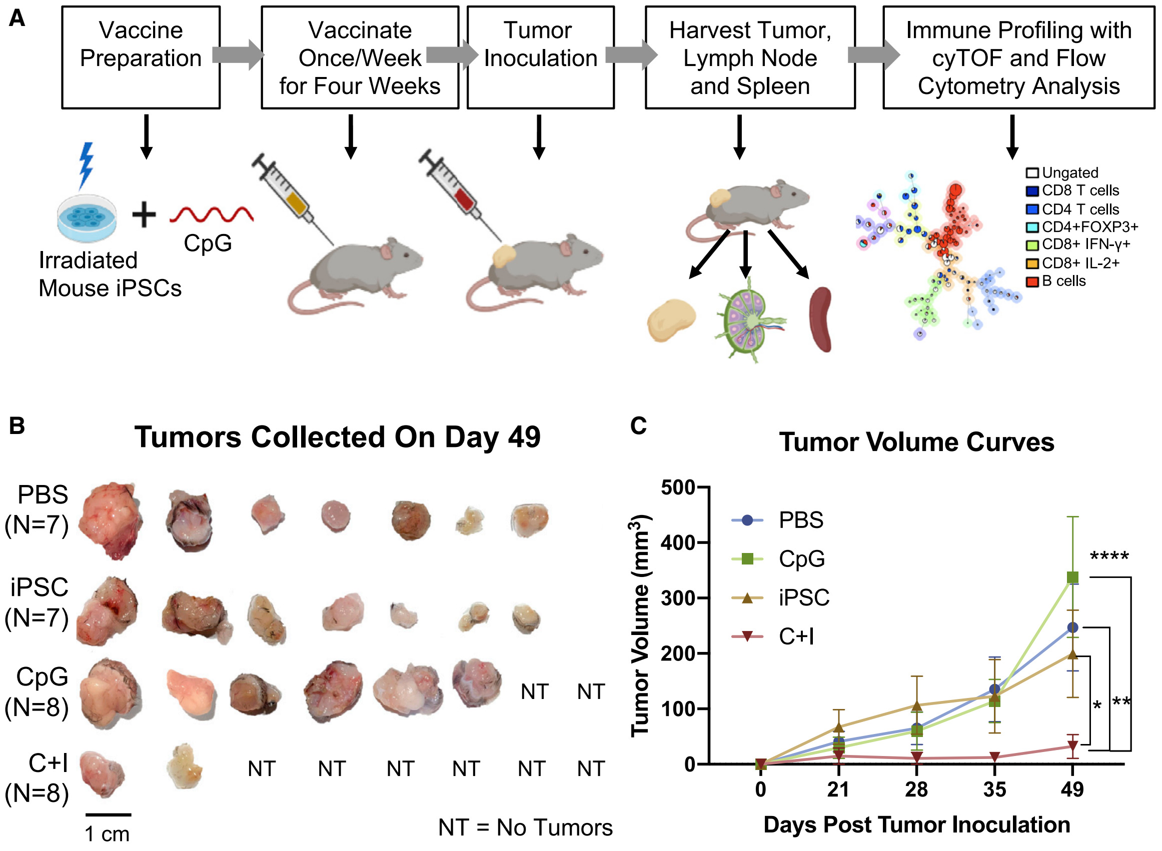

Figure 1. A murine iPSC vaccine prevents tumor formation *in vivo*

(A) Diagram showing vaccine preparation consists of sorting murine iPSCs for pluripotency, irradiation, resuspension in adjuvant solution, and subcutaneous injection in the flank. Mice were randomized into different treatment groups and were vaccinated with the C + I vaccine, irradiated iPSCs alone, CpG alone, or PBS for 4 weeks.

(B) Vaccination of mice with the C + I vaccine resulted in a complete rejection of the cancer cells in six out of eight mice by day 49 and overall reductions in tumor size (n = 7–8 per group; representative images).

(C) Quantification of the tumor volume over time, with values being expressed as means ± SEM (n = 7–8, *p < 0.05, **p < 0.001, ****p < 0.0001, Tukey's multiple comparison test). Experiments were repeated three times to a total number of seven to eight mice per group.

oligodeoxynucleotide [ODN] containing unmethylated CpG motifs) that promotes antigen-presenting cell (APC) maturation (Ballas et al., 2001). Gamma irradiation was needed to prevent teratoma formation by the iPSCs. Mice were injected subcutaneously with (1) phosphate-buffered saline (PBS) control, (2) CpG alone, (3) iPSCs alone, or (4) the combination of CpG + iPSCs (C + I) once a week for 4 weeks (n = 7–8/group), and then inoculated at a separate site with Pan02, a syngeneic murine PDAC line (Corbett et al., 1984) (Figure 1A). In the C + I group, 75% of the vaccinated mice (6/8) completely rejected cancer cells (Figure 1B). In the C + I-vaccinated mice, the mean tumor volume was significantly lower than in mice treated with PBS (p = 0.0050), iPSCs (p = 0.0448), or CpG (p < 0.0001) by day 49 after tumor inoculation (Figure 1C). Interestingly, the CpG alone group had the largest tumor sizes, which was also observed in an orthotopic breast cancer model in a previous study (Kooreman et al., 2018). Histological analysis confirmed the presence of neoplastic cells within the excised tumor from mice treated with PBS, CpG, iPSCs, and the C + I vaccine that developed tumors, and the lack of iPSC-derived teratoma formation in these mice (Figure S1). These results demonstrated the effectiveness and the antitumor effects of the iPSC-based cancer vaccine in PDAC.

To define the mechanism underlying the effectiveness of the iPSC vaccine, we next performed immune profiling on vaccinated mice. Because most of the C + I-vaccinated mice did not develop tumors (~75%), we harvested tumor-draining lymph nodes (TDLNs) from each group and performed cytometry by time-of-flight (CyTOF) analysis to assess potential differences in the frequencies of immune cell populations among the treatment groups. An unsupervised machine learning algorithm, "FlowSOM," was used for clustering live $CD45^+$ cells (Figure S2A) (Gassen et al.,

2015). We manually annotated the T cell populations ($CD8^+$ T cells, $CD4^+$ T cells) based on the median marker intensities in the clusters, and overlaid the cell populations on t-distributed stochastic neighbor-embedding plots for each group (Figure 2A). The results show that C + I immunization significantly increased the frequency of $CD8^+$ cytotoxic T cells compared with PBS control. Minimum spanning trees also revealed striking differences in $CD8^+$ cytotoxic T cells (metacluster 10 in the pink shade) between the C + I-vaccinated and PBS control groups (Figures 2B, 2C, S2B, and S2C). Of note, large portions of $CD8^+$ T cells in the C + I vaccine group, but not in the PBS group, were interferon-γ (IFN-γ) positive and interleukin-2 (IL-2) positive (Figure 2B), indicating enhanced immune activation of these cytotoxic $CD8^+$ T cells. We also observed significantly higher frequencies of $CD69^+CD8^+$ T cells and IFN-γ^+ and IL-2^+ $CD8^+$ T cells in the TDLNs of the C + I vaccine group compared with PBS group (Figure 2D). A fold-change analysis using the spanning-tree progression analysis of density-normalized events showed upregulation of IFN-γ and IL-2 that was induced by the C + I vaccine compared with PBS controls in not only $CD8^+$ T cells, but also in $CD4^+$ T, B cells, and circulating dendritic cells (DCs) in TDLNs (Figures 3A and 3B). Collectively, these data suggest that the C + I vaccine induced the activation of multiple immune effector cell types in TDLNs.

Tregs can accumulate in the tumor microenvironment to suppress TAA-specific immunity, hence inhibiting antitumor immunity (Bonertz et al., 2009). The FlowSOM MTS revealed a decrease in $CD4^+CD25^+FOXP3^+$ Tregs, as indicated in cluster 6 (light blue shade), in the C + I vaccine group compared with the PBS group (Figures 2B, S2B, and S2C). We quantified the frequencies of $CD4^+CD25^+FOXP3^+$ Tregs in all groups and found a significant reduction in Tregs in the C + I vaccine group compared with PBS in both TDLNs and the spleen (Figure 3C), reversing the immune-suppressive microenvironment in mice injected with cancer cells. However, neither the iPSCs alone nor CpG alone treatments reduced the Treg population, and thus both were ineffective in inhibiting tumor growth; in fact, they tended to increase Tregs in TDLNs. These results indicate that the combination of iPSC + CpG exerts a synergistic effect in activating the immune system and inducing antitumor immunity.

Besides Tregs, other tumor-promoting immune cells, such as IL-17-producing-$CD4^+$ T cells (T helper 17 [Th17]) (Grivennikov et al., 2012) have also been reported to be a tumor-promoting immune cell type in PDAC murine models (Hegde et al., 2020). In PDAC patients, Th17 are pro-tumor cells and are correlated with poor patient survival (He et al., 2011). In our study, we observed that iPSCs alone increased the frequency of IL-17^+CD4^+ Th17 cells in TDLNs compared with PBS control, whereas the C + I vaccine significantly decreased the relative frequency of these cells compared with the iPSCs alone group, and reversed the tumor-promoting immune environment (Figure 3D). Collectively, these data show that the C + I vaccine stimulated anti-cancer cytotoxic T cell responses and suppressed immune-suppressive regulatory T cells.

CpG ODNs have been shown to be able to strongly activate B cells and weakly stimulate plasmacytoid DCs (pDCs) (Krieg et al., 1995; Krug et al., 2001). To determine whether the C + I vaccine induced cancer cell-specific antibodies, we performed serum immunoglobulin G (IgG) binding assay to determine the iPSC- and cancer cell-specific serum IgG levels in PBS and C + I vaccine-treated mice. We found significant increases in iPSC- and cancer cell-specific serum IgG levels, but no significant changes in non-specific IgG that bound to mouse fibroblasts (Figure S3A). These data suggest that the C + I vaccine stimulated a humoral immune response against cancer cells.

To determine whether C + I vaccine increased pDC recruitment in TDLN, we evaluated the percentage of pDCs in the TDLN in PBS and C + I-vaccinated mice. We found that there was a trend of induction of pDC recruitment in the TDLN for the C + I-vaccinated mice compared with PBS control mice (Figure S3B). Collectively, these data suggest that the C + I vaccine could activate B cell responses and increase cancer cell-specific antibodies, and potentially also increase recruitment of pDCs in TDLN.

To determine whether the C + I vaccine stimulated cancer cell-specific T cell memory responses in mice, we performed flow cytometry analysis on splenocytes harvested from mice treated with the C + I vaccine or PBS control, and stimulated the splenocytes with PBS or cancer cell lysate from Panc02 cells. $CD8^+$ cytotoxic T cells from C + I-vaccinated mice produced more IFN-γ^+ upon cancer cell lysate stimulation compared with PBS control-treated mice. Without cancer cell lysate stimulation, no significant difference in IFN-γ^+ production in $CD8^+$ T cells in the spleen was observed in C + I-vaccinated mice or PBS control mice (Figure S3C). These data suggest that a cancer cell-specific $CD8^+$ cytotoxic T lymphocyte memory was established in C + I vaccine-treated mice.

Importantly, we also found that the C + I vaccine did not induce significant systemic cytokine production without re-stimulation in peripheral organs, such as the spleen (Figure S3D), nor did it affect the overall appearance or body weights of the mice (Figure S3E), suggesting that the C + I vaccine treatment did not cause significant systemic toxicity and was well tolerated by the mice.

To investigate whether the iPSC vaccine has the potential to provide TAAs specific for PDAC, we first compared the transcriptomics of mouse and human iPSCs and PDAC cancer cells. We evaluated whether there are shared upregulated genes between mouse and human iPSCs with mouse and human pancreatic cancer lines. We performed RNA-seq

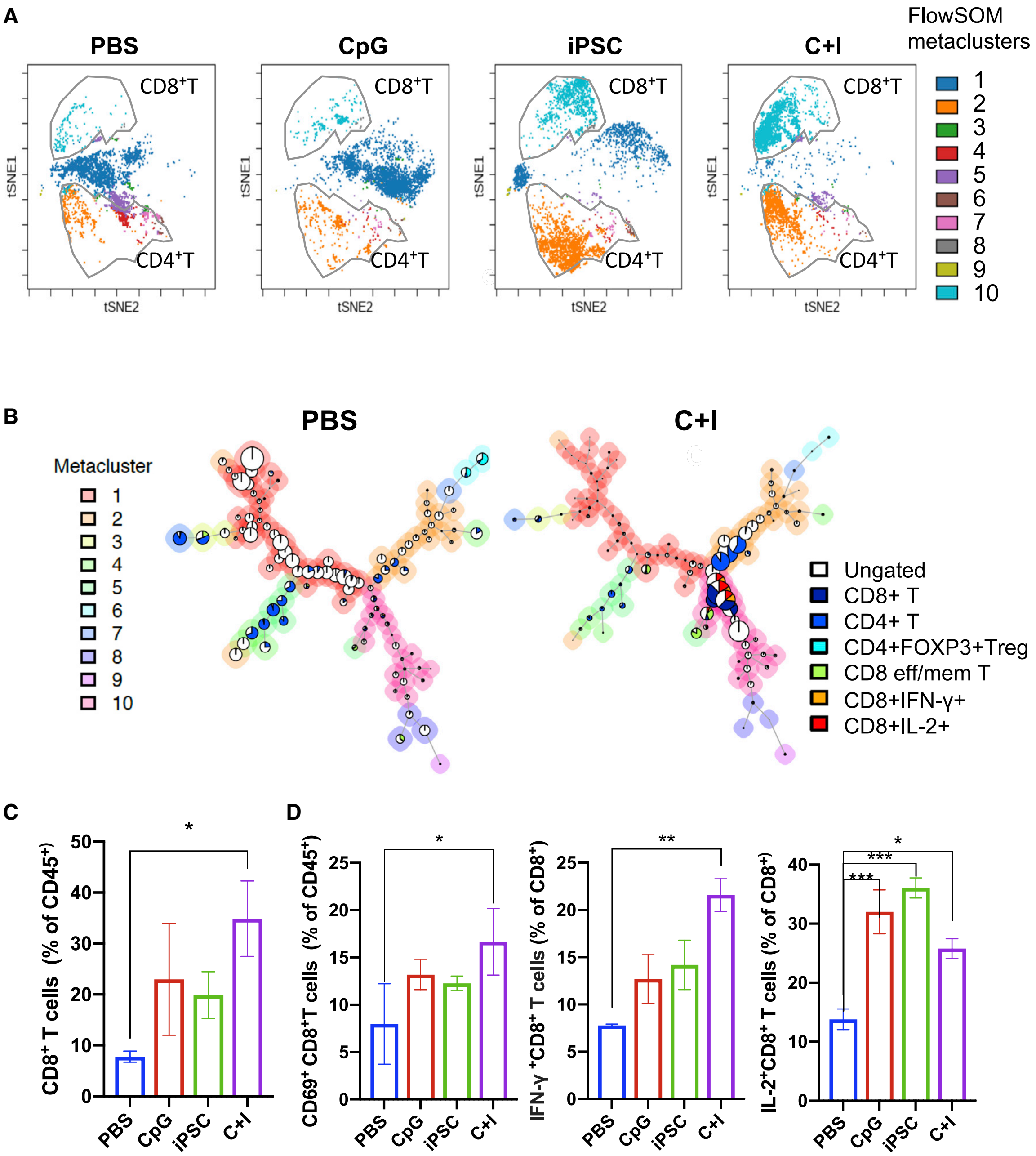

Figure 2. Differences in $CD8^+$ T cell activation status and frequency of the T cell subpopulations in TDLNs after iPSC vaccine and control treatments

(A) t-Distributed stochastic neighbor embedding (tSNE) visualization of FlowSOM-generated clusters (live $CD45^+$ cells) in merged data from cells in draining lymph nodes from mice treated with PBS, CpG alone, iPSCs alone, or the C + I vaccine. $CD8^+$ T and $CD4^+$ T cells were manually gated and overlaid on tSNE based on marker expression.

(B) FlowSOM results from one representative mouse treated with PBS (left panel) or the C + I vaccine (right panel) as minimum spanning trees. FlowSOM was performed using 225 clusters and 10 metaclusters. Each cluster is represented by 1 pie chart, and metaclusters are denoted by background shading.

(C) Percentage of $CD8^+$ T cells among $CD45^+$ cells in TDLNs from mice treated with PBS, CpG, iPSCs, or the C + I vaccine (n = 3, mean ± SEM, *$p < 0.05$, compared with PBS, Dunnett's multiple comparison test).

(D) Percentage of activated $CD69^+CD8^+$ T cells among $CD45^+$ cells in TDLNs from mice treated with PBS, CpG, iPSCs, or the C + I vaccine (left). Percentages of IFN-g$^+$$CD8^+$ and IL-2$^+$$CD8^+$ T cells among $CD8^+$ T cells in TDLNs from mice treated with PBS, CpG, iPSCs, or the C + I vaccine (middle and right) (n = 3, mean ± SEM, *$p < 0.05$, **$p < 0.01$, ***$p < 0.001$ compared with PBS, Dunnett's multiple comparison test).

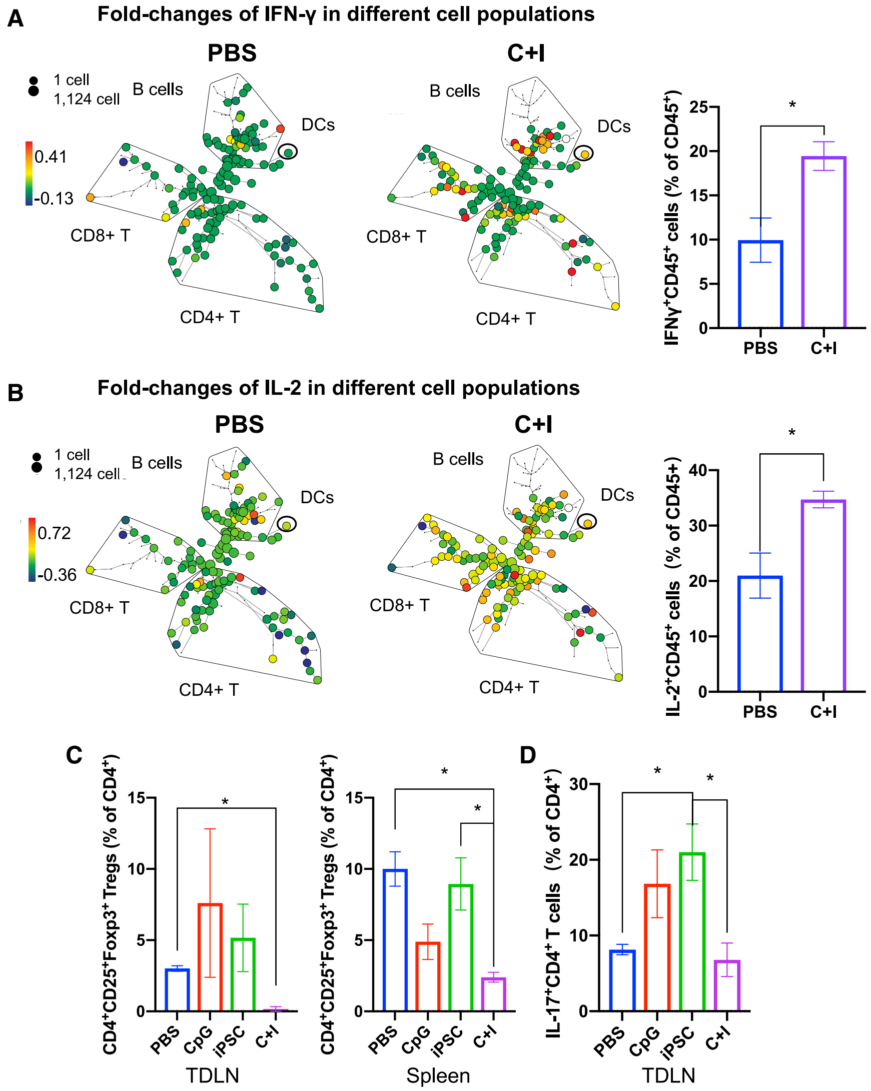

Figure 3. Increased activation in multiple immune cell types and decreased immune suppression after the iPSC vaccine in mice

(A and B) Spanning-tree progression analysis of density-normalized events analysis of live $CD45^+$ cells in TDLNs from mice treated with PBS and the C + I vaccine with the fold-change intensity of IFN-γ^+ and IL-2^+ cells indicated as the color. Frequencies of IFN-γ^+ and IL-2^+CD45^+ cells of total live $CD45^+$ cells (n = 3, means ± SEM, *$p < 0.05$ compared with PBS, Student's t test).

(C) Percentage of $CD4^+CD25^+FOXP3^+$ Treg cells among $CD4^+$ T cells in TDLNs and spleen from mice treated with PBS, CpG, iPSCs, or the C + I vaccine.

(D) Percentage of IL-17^+CD4^+ T cells among $CD4^+$ T cells in TDLNs (right) from mice treated with PBS, CpG, iPSCs, or the C + I vaccine (n = 3–4, mean ± SEM, *$p < 0.05$ compared with PBS, Tukey's multiple comparison test).

analysis on mouse and human iPSCs, mouse and human pancreatic cancer lines, and fibroblasts lines. We found shared upregulated genes between mouse/human iPSCs and mouse/human pancreatic cancer lines that are only minimally expressed by mouse or human fibroblasts (Figure S4A).

To extend our study in more cancer types, we investigated the possibility of shared gene expression signatures that are highly expressed by human iPSC lines and multiple cancer lines, but not in normal cell lines. We previously found that human and mouse iPSC lines share their gene expression profiles with those of human and mouse cancer cell lines from multiple cancer types (Kooreman et al., 2018). This analysis revealed 111 upregulated tumor-associated genes that are shared by iPSCs and cancer cells (Table S1). These 111 genes, which we call iPSC-cancer signature genes in this study, are highly expressed by pluripotent populations but only marginally or not at all by the somatic tissues (Kooreman et al., 2018).

To first determine whether the iPSC-cancer signature genes are enriched in mouse PDAC cells and iPSCs that we used in our mouse model, we performed gene set enrichment analysis using RNA-seq data on Panc02 cells and mouse iPSCs, and used the iPSC-cancer signature genes as a user-defined gene set. We found that the expression of the iPSC-cancer signature gene set is enriched in mouse PDAC cell line Panc02 cells and mouse iPSCs compared with mouse embryonic fibroblasts (Figures S4B and S4C).

To further investigate whether the iPSC-cancer signature is elevated in multiple human cancer types, we examined the expression levels of these genes in human tumors in The Cancer Genome Atlas (TCGA) database. An evaluation of the mRNA expression levels of the iPSC-cancer signature genes in human solid tumors in TCGA PanCancer Atlas

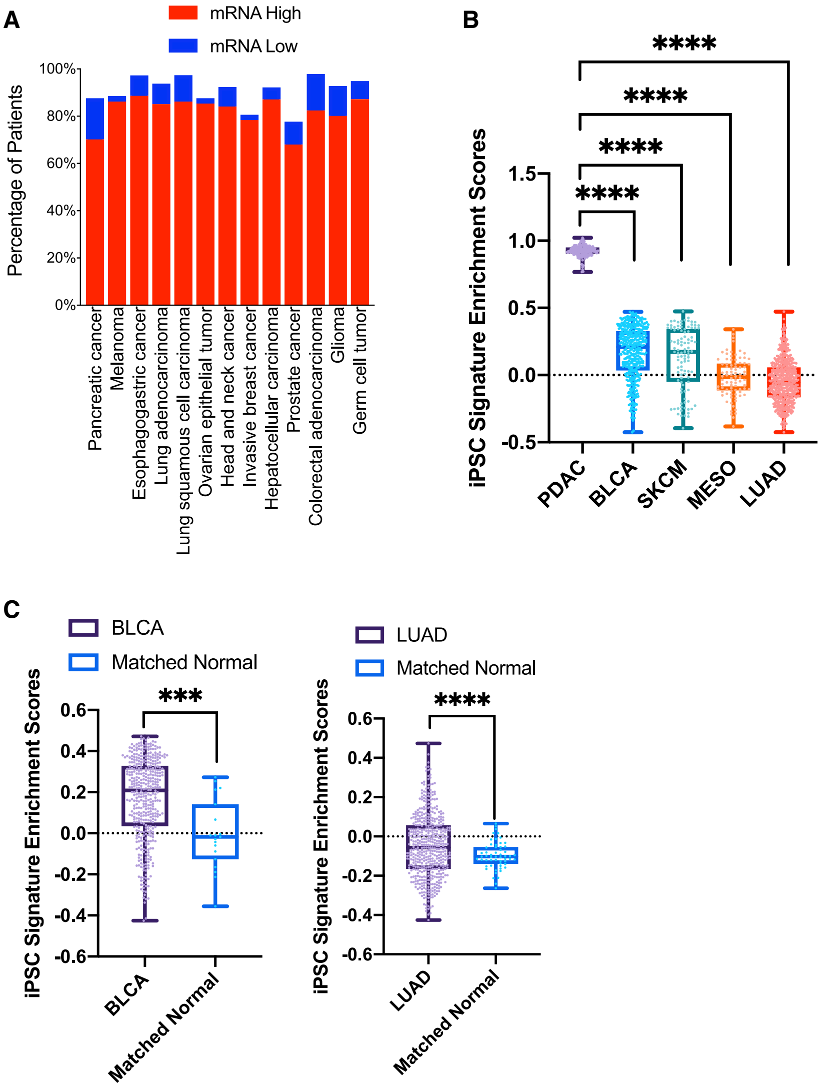

Figure 4. iPSC-cancer signature genes are upregulated in human tumors in TCGA cohorts
(A) Analysis of 111 iPSC-cancer signature gene mRNA expression in major human cancer types in TCGA cohorts (*Z* scores > 2 or *Z* scores < −2).
(B) Gene enrichment scores of iPSC-cancer signature genes in five cancer types. SKCM, primary skin cutaneous melanoma. PDAC, pancreatic ductal adenocarcinoma. BLCA, bladder urothelial carcinoma. LUAD, lung adenocarcinoma. MESO, mesothelioma (****$P < 0.0001$ compared with PDAC, Tukey's multiple comparison test).
(C) Enrichment scores of iPSC-cancer signature genes in tumors and matched normal tissues in BLCA and LUAD (***$P < 0.001$, ****$P < 0.0001$, Student's t test).

Studies (Hoadley et al., 2018; Witkiewicz et al., 2015) revealed high levels of mRNA upregulation in patients' tumors, ranging from 68.1% to 88.7% (Figure 4A), and smaller proportions of mRNA downregulation (compared with all samples that are diploid for that gene in the signature, *Z* score > 2). These data suggest that overexpression of iPSC-cancer signature genes is common in human solid tumors, confirming the resemblance of human tumor cells to iPSCs.

To further investigate the enrichment status of the iPSC-cancer shared genes as a signature, we computed the gene set enrichment scores for the 111 genes as the iPSC-cancer signature genes in five different cancer types in TCGA Pan-Cancer Atlas patients, including pancreatic ductal adenocarcinoma (PDAC), lung adenocarcinoma (LUAD), skin cutaneous melanoma (SKCM), bladder carcinoma (BLCA), and mesothelioma (MESO). We found that the iPSC-cancer signature genes are positively enriched in all PDAC tumors, in the majority of BLCA and primary SKCM tumors, in half of the MESO tumors, and in more than a quarter of LUAD patients (Figure 4B). Notably, in cancer types with sufficient matched adjacent normal samples, such as BLCA and LUAD, the tumor samples have significantly higher enrichment scores than their matched normal counterparts (Figure 4C), suggesting possible roles of these genes in tumor development. Importantly, PDAC tumors not only all have positive enrichment scores, but also have the highest enrichment scores in these genes compared with other cancer types (Figure 4B), indicating that these genes may be particularly important in PDAC.

To determine whether the iPSC-cancer signature genes play direct roles in regulating the immune cells, we performed pathway enrichment analysis of the iPSC-cancer signature genes using the Reactome database, and found

that ten of the iPSC-cancer signature genes are involved in immune system-related pathways (Table S2).

As the C + I vaccine stimulated antitumor T cell immune responses, we next sought to identify potential peptide vaccine antigens in the iPSC-cancer signature genes that can be presented to T cells. We scanned 111 iPSC-cancer signature genes with machine learning algorithms, NetMHC-pan4.0 (Jurtz et al., 2017) and MARIA (Chen et al., 2019), to identify T cell epitopes highly presentable by human major histocompatibility complex class I (MHC I) and MHC II. Both algorithms are trained not only on binding affinities but also on naturally presented peptides (ligands), taking the antigen presentation process information into account. Optimal vaccine antigens should be presented by multiple MHC I and MHC II alleles in a general population. We ranked candidate T cell epitopes by binding affinity and selected epitopes that are presentable by more than 50% of inputted common MHC I alleles and 100% of inputted common MHC II alleles among the US population. We identified 11 genes that contain T cell epitopes that are highly presentable to both MHC I and MHC II alleles (Table S3). To validate our prediction, we then assessed whether any candidate has been previously demonstrated as a T cell epitope by searching these top candidates with 90% sequence similarity in the Immune Epitope Database. We found that 8 out of the 11 genes contain multiple T cell epitopes that have been previously reported to be recognized by T cells with experimental evidence (Table S3).

DISCUSSION

In this study, we deployed an iPSC-based cancer vaccine together with an immune adjuvant to target TAAs in PDAC. We evaluated the efficacy of an iPSC-based cancer vaccine and assessed the immune-stimulatory effects of this cancer vaccine in a murine PDAC model. We found that the iPSC-cancer vaccine completely prevented tumor development in 75% of the mice and stimulated antitumor T cell and B cell responses. The iPSC vaccine significantly increased $CD8^+$ cytotoxic T cell frequency in the tumor TDLN and promoted T cell activation and antitumor cytokine production (IFN-γ and IL-2) in T lymphocytes, and increased cancer cell-specific antibodies in B cells. Our results demonstrated the effectiveness and the immune-stimulatory effects of this iPSC-based cancer vaccine in PDAC.

The ineffectiveness of current immune checkpoint inhibitors in PDAC may be due to the non-immunogenic and low antigenic nature of these tumors. A clue to the immunogenicity and antigenicity of tumors is tumor mutational load, which has been considered as a response predictor for immune checkpoint inhibitors (Goodman et al., 2017). Studies indicate that a high tumor mutational burden increases the chance that T cells respond and expand to immunogenic neoantigens on tumor cells. Thus, immune checkpoint inhibitors are more effective in tumors with high tumor mutational burden. However, PDAC has a relatively lower somatic mutational burden compared with other immune checkpoint inhibitor-responsive cancers (Chalmers et al., 2017). Interestingly, PDAC tumors are highly enriched in iPSC-cancer signature genes. This suggests that these iPSC genes may have critical roles in the development and progression of PDAC.

The immune system can identify cancer cells by recognizing non-mutated TAAs. Cancer vaccines based on TAAs, such as prostatic acid phosphatase, NY-ESO-1 (cancer/testis antigen), MAGE-A3, and glypican 3, were shown to be immunogenic and induced clinical responses in a subset of patients (Atanackovic et al., 2008; Cheever and Higano, 2011; Vansteenkiste et al., 2016). However, some normal tissues also express low levels of TAAs during development; therefore, the immune system is programmed to develop tolerance toward these antigens by upregulating the immune-suppressive mechanisms, such as Tregs. Treg-mediated suppression of TAA-reactive T cells has been proposed as a potential mechanism for the failure of some TAA-based cancer vaccines (Sakaguchi, 2005).

Our data show that the iPSC-cancer vaccine significantly decreased the $CD4^+CD25^+FOXP3^+$ Treg population in TDLN and spleen, reversing the immune-suppressive microenvironment in mice injected with cancer cells. However, neither the iPSCs alone nor CpG alone treatments reduced the Treg population and thus both were ineffective in inhibiting tumor growth. These results indicate that the combination of iPSC + CpG exerts a synergistic effect in activating the immune system and inducing antitumor immunity. These data also suggest that the TAA-based iPSC vaccine is a potentially effective anti-cancer strategy suitable for PDAC, which has a low mutational burden and is currently non-responsive to immune checkpoint inhibitors in patients.

Our data show that iPSCs alone did not significantly prevent PDAC formation in mice, and that the addition of CpG is required to achieve the tumor preventive effects. CpGs are unmethylated synthetic oligonucleotides (ODNs) that mimic microbial DNA and thus can activate the Toll-like receptor 9 (TLR9) on APCs, including macrophages, DCs, and B cells (Krieg, 2007). CpG has been investigated in cancer vaccines as an immune adjuvant, as it can improve the function of professional APCs and boost the generation of cellular and humoral vaccine antigen-specific immune responses. Our data show that the induction of both cancer cell-specific T and B cell immune responses in the CpG + iPSCs group compared with the PBS control group, confirming the need of including CpG in the iPSC vaccine.

In this study, we report the iPSC-cancer gene signature consisting of 111 cancer-associated genes that is also highly expressed in iPSCs. However, none of these genes has a well-known anti-cancer immunity-related function. Comparison analysis of transcriptomes of cancer cells of different types, iPSCs clones, and multiple normal tissues using large datasets to identify genes that are only expressed in iPSCs and cancer cells, but not in normal tissues, is still needed. Peptide antigens in iPSCs that can induce the tumor preventive effects and anti-cancer immune responses are under investigation.

The concept of using embryonic stem cells (ESCs) or iPSCs as a prophylactic cancer vaccine has been evaluated in multiple studies in different murine tumor models, including models for lung cancer (Dong et al., 2010; Wang et al., 2020; Yaddanapudi et al., 2012), melanoma (Gąbka-Buszek et al., 2020; Kooreman et al., 2018), ovarian cancer (Zhang et al., 2012), colon cancer (Li et al., 2009), and mesothelioma (Kooreman et al., 2018). These studies show the efficacy of ESC/iPSC-based cancer vaccines in preventing tumor development in mouse models in those cancer types and show the potential of these vaccines as promising prophylactic cancer vaccines, suggesting the need of testing these vaccines in more preclinical models and eventually clinical settings.

Although generating an autologous iPSC line for each patient seems to be less feasible and a prophylactic cancer vaccine seems to be less relevant to clinical medicine at present, under certain scenarios the iPSC-based cancer vaccine described in our study has significant merits as a future immune therapy in clinical settings. Firstly, establishing an autologous iPSC line for every patient is not necessary, as hypoimmunogenic iPSCs can be generated by inactivating MHC I and MHC II genes as a universal iPSC transplantation source for potential clinical use (Deuse et al., 2019). Secondly, in a prophylactic setting, the iPSC vaccine can be generated to treat individuals at high risk for developing cancers, such as patients with Lynch syndrome, Li-Fraumeni syndrome, hereditary chronic pancreatitis (Lowenfels et al., 1997; Weiss, 2014), chronic hepatitis B infection (Sherman et al., 1995), or pathogenic germline mutations in BRCA1/2 genes. These patients have a much higher likelihood of developing cancer in their lifetime and thus may be suitable candidates for prophylactic cancer vaccines. Thirdly, the iPSC vaccine can also be used as an adjuvant immunotherapy. We previously showed that, as an adjuvant, the iPSC vaccine inhibited melanoma recurrence at the resection site and reduced metastatic tumor load (Kooreman et al., 2018). The iPSC vaccine could be developed at the time of diagnosis and available at the time of surgical or chemo/radiotherapy treatment of the cancer. Under these scenarios, the clinical development of the iPSC-based cancer vaccine described in our study is warranted and feasible.

In summary, our study demonstrates that the iPSC-based cancer vaccine prevented tumor formation, induced antitumor effector and memory T cell responses and B cell responses, and reduced immune-suppressive Tregs in PDAC, possibly due to synergistic effects of TAAs provided by the iPSCs and APC activation induced by the CpG. We also show the expression and enrichment of iPSC-cancer signature genes in human solid tumors compared with matched adjacent normal tissues, which highlights the clinical relevance of the iPSC-based cancer vaccine in human tumors. Compared with other immunological modalities, iPSC vaccination presents a broad-spectrum of non-mutated tumor antigens to the immune system, potentially making this approach applicable to PDAC and other cancers with low tumor mutational burdens. Further validation of the TAAs in the iPSCs could yield novel peptide-based cancer vaccines suitable for patients with low mutational burden tumors who are non-responsive to immune checkpoint inhibitor or neoantigen vaccine.

EXPERIMENTAL PROCEDURES

Mouse pancreatic tumor model

Young adult female C57BL/6J (6–8 weeks old) were used. Animals were randomly assigned to different treatment groups (n = 7–8 per group). All experiments were approved by the Stanford University Administrative Panel of Laboratory Animal Care. The murine PDAC cell line Pan02 was a gift from Dr. Aida Habtezion (Stanford University). It was derived from C57BL/6 mice (Corbett et al., 1984). The cancer cells were grown in DMEM and 10% heat-inactivated fetal bovine serum under standard culture conditions. For cancer cell inoculation, 5×10^4 Pan02 cells were resuspended in DMEM without serum and injected subcutaneously in the middle-lower back of the mice. Tumor growth was measured weekly by caliper. Seven weeks after tumor inoculation, mice were euthanized, and tumors, spleens, and TDLNs were harvested for immune profiling.

iPSC vaccine preparation and immunization

For each mouse, 2×10^6 autologous (C57BL/6J) murine iPSCs were sorted for a pluripotent marker SSEA-1 and were irradiated at 6,000 rads before injection. Irradiated iPSCs were suspended in 5 μM CpG ODN1826 (Invivogen) in PBS and loaded into 28G insulin syringes (Terumo). Mice were anesthetized with 2% isoflurane (Isothesia, Butler Schein) in 100% oxygen until the loss of righting reflex. Immunization was performed by subcutaneous injection of the vaccine in the flanks of the mice, with the injection site alternating every week. Mice were monitored weekly for general health by gross examination of overall appearance and weight measurements.

Data and code availability

The data that support the findings of this study are available upon request.

SUPPLEMENTAL INFORMATION

Supplemental information can be found online at https://doi.org/10.1016/j.stemcr.2021.04.004.

AUTHOR CONTRIBUTIONS

X.O. and J.C.W. conceived the idea and hypothesis. X.O. designed and performed the experiments, data analysis, and wrote the manuscript. Y.L. performed ssGSEA analysis, correlation analysis, and RNA-seq analysis. Y.Z. helped with mouse iPSC irradiation. Y.Z. and L.W. assisted with mouse spleen and lymph node tissue processing. J.G. conjugated anti-FOXP3, anti-Ki67, and anti-CD3 antibodies for cyTOF and provided input on experimental design. T.W. assisted with mouse tissue dissection. C.L. provided technical guidance on mouse iPSC culture. B.L. provided technical guidance on flow cytometry and provided input on experimental design. B.C. and A.Z. provided technical guidance on MARIA. K.M.C. reviewed the histology of the tumors. A.H., N.G.K., E.G.E., and J.C.W. provided critical input on experimental design.

CONFLICTS OF INTERESTS

J.C.W. is the co-founder of Khloris Biosciences but has no competing interests as the work presented here is completely independent.

ACKNOWLEDGMENTS

We thank the Stanford Animal Histology Services for help with the preparation of histologic specimens; Xiaolan Zhang for assistance in mouse spleen and lymph node tissue processing; and Lisa Nichols, Cindy Jiang, and Meredith Weglarz for the technical support on the CyTOF and flow cytometry instruments. We thank Dr. Patricia Nguyen for critical feedback on the manuscript. Funding support was provided by the National Institutes of Health (NIH) T32 HL098049 (to X.O.), NIH R01 HL141851, R01 HL123968, R01 HL132875, and Stanford Cancer Institute Cancer Innovation Award (to J.C.W.), and 1U54CA209971 and 1R01CA222969 (to E.G.E). CyTOF data were collected on an instrument in the Shared FACS Facility obtained under an NIH S10 Shared Instrument Grant (S10OD016318-01).

Received: October 2, 2020
Revised: April 8, 2021
Accepted: April 9, 2021
Published: May 6, 2021

REFERENCES

Atanackovic, D., Altorki, N.K., Cao, Y., Ritter, E., Ferrara, C.A., Ritter, G., Hoffman, E.W., Bokemeyer, C., Old, L.J., and Gnjatic, S. (2008). Booster vaccination of cancer patients with MAGE-A3 protein reveals long-term immunological memory or tolerance depending on priming. Proc. Natl. Acad. Sci. U S A *105*, 1650–1655.

Ballas, Z.K., Krieg, A.M., Warren, T., Rasmussen, W., Davis, H.L., Waldschmidt, M., and Weiner, G.J. (2001). Divergent therapeutic and immunologic effects of oligodeoxynucleotides with distinct CpG motifs. J. Immunol. *167*, 4878–4886.

Bonertz, A., Weitz, J., Pietsch, D.-H.K., Rahbari, N.N., Schlude, C., Ge, Y., Juenger, S., Vlodavsky, I., Khazaie, K., Jaeger, D., et al. (2009). Antigen-specific Tregs control T cell responses against a limited repertoire of tumor antigens in patients with colorectal carcinoma. J. Clin. Invest. *119*, 3311–3321.

Chalmers, Z.R., Connelly, C.F., Fabrizio, D., Gay, L., Ali, S.M., Ennis, R., Schrock, A., Campbell, B., Shlien, A., Chmielecki, J., et al. (2017). Analysis of 100,000 human cancer genomes reveals the landscape of tumor mutational burden. Genome Med. *9*, 34.

Cheever, M.A., and Higano, C.S. (2011). PROVENGE (Sipuleucel-T) in prostate cancer: the first FDA-approved therapeutic cancer vaccine. Clin. Cancer Res. *17*, 3520–3526.

Chen, B., Khodadoust, M.S., Olsson, N., Wagar, L.E., Fast, E., Liu, C.L., Muftuoglu, Y., Sworder, B.J., Diehn, M., Levy, R., et al. (2019). Predicting HLA class II antigen presentation through integrated deep learning. Nat. Biotechnol. *37*, 1332–1343.

Corbett, T.H., Roberts, B.J., Leopold, W.R., Peckham, J.C., Wilkoff, L.J., Griswold, D.P., and Schabel, F.M. (1984). Induction and chemotherapeutic response of two transplantable ductal adenocarcinomas of the pancreas in C57BL/6 mice. Cancer Res. *44*, 717–726.

Deuse, T., Hu, X., Gravina, A., Wang, D., Tediashvili, G., De, C., Thayer, W.O., Wahl, A., Garcia, J.V., Reichenspurner, H., et al. (2019). Hypoimmunogenic derivatives of induced pluripotent stem cells evade immune rejection in fully immunocompetent allogeneic recipients. Nat. Biotechnol. *37*, 252–258.

Dong, W., Du, J., Shen, H., Gao, D., Li, Z., Wang, G., Mu, X., and Liu, Q. (2010). Administration of embryonic stem cells generates effective antitumor immunity in mice with minor and heavy tumor load. Cancer Immunol. Immunother *59*, 1697–1705.

Gąbka-Buszek, A., Kwiatkowska-Borowczyk, E., Jankowski, J., Kozłowska, A.K., and Mackiewicz, A. (2020). Novel genetic melanoma vaccines based on induced pluripotent stem cells or melanosphere-derived Stem-like cells display high efficacy in a murine tumor rejection model. Vaccines *8*, 147.

Gassen, S.V., Callebaut, B., Helden, M.J.V., Lambrecht, B.N., Demeester, P., Dhaene, T., and Saeys, Y. (2015). FlowSOM: using self-organizing maps for visualization and interpretation of cytometry data. Cytometry A *87*, 636–645.

Goodman, A.M., Kato, S., Bazhenova, L., Patel, S.P., Frampton, G.M., Miller, V., Stephens, P.J., Daniels, G.A., and Kurzrock, R. (2017). Tumor mutational burden as an independent predictor of response to immunotherapy in diverse cancers. Mol. Cancer Ther. *16*, 2598–2608.

Grivennikov, S.I., Wang, K., Mucida, D., Stewart, C.A., Schnabl, B., Jauch, D., Taniguchi, K., Yu, G.-Y., Osterreicher, C.H., Hung, K.E., et al. (2012). Adenoma-linked barrier defects and microbial products drive IL-23/IL-17-mediated tumour growth. Nature *491*, 254–258.

He, S., Fei, M., Wu, Y., Zheng, D., Wan, D., Wang, L., and Li, D. (2011). Distribution and clinical significance of Th17 cells in the tumor microenvironment and peripheral blood of pancreatic cancer patients. Int. J. Mol. Sci. *12*, 7424–7437.

Hegde, S., Krisnawan, V.E., Herzog, B.H., Zuo, C., Breden, M.A., Knolhoff, B.L., Hogg, G.D., Tang, J.P., Baer, J.M., Mpoy, C., et al.

(2020). Dendritic cell paucity leads to dysfunctional immune surveillance in pancreatic cancer. Cancer Cell *37*, 289–307.e9.

Hoadley, K.A., Yau, C., Hinoue, T., Wolf, D.M., Lazar, A.J., Drill, E., Shen, R., Taylor, A.M., Cherniack, A.D., Thorsson, V., et al. (2018). Cell-of-origin patterns dominate the molecular classification of 10,000 tumors from 33 types of cancer. Cell *173*, 291–304.e6.

Ilyas, S., and Yang, J.C. (2015). Landscape of tumor antigens in T cell immunotherapy. J. Immunol. *195*, 5117–5122.

Jurtz, V., Paul, S., Andreatta, M., Marcatili, P., Peters, B., and Nielsen, M. (2017). NetMHCpan-4.0: improved peptide–MHC class I interaction predictions integrating eluted ligand and peptide binding affinity data. J. Immunol. *199*, 3360–3368.

Kooreman, N.G., Kim, Y., de Almeida, P.E., Termglinchan, V., Diecke, S., Shao, N.-Y., Wei, T.-T., Yi, H., Dey, D., Nelakanti, R., et al. (2018). Autologous iPSC-based vaccines elicit anti-tumor responses in vivo. Cell Stem Cell *22*, 501–513.e7.

Krieg, A.M. (2007). Antiinfective applications of toll-like receptor 9 agonists. Proc. Am. Thorac. Soc. *4*, 289–294.

Krieg, A.M., Yi, A.K., Matson, S., Waldschmidt, T.J., Bishop, G.A., Teasdale, R., Koretzky, G.A., and Klinman, D.M. (1995). CpG motifs in bacterial DNA trigger direct B-cell activation. Nature *374*, 546–549.

Krug, A., Rothenfusser, S., Hornung, V., Jahrsdörfer, B., Blackwell, S., Ballas, Z.K., Endres, S., Krieg, A.M., and Hartmann, G. (2001). Identification of CpG oligonucleotide sequences with high induction of IFN-alpha/beta in plasmacytoid dendritic cells. Eur. J. Immunol. *31*, 2154–2163.

Li, Y., Zeng, H., Xu, R.-H., Liu, B., and Li, Z. (2009). Vaccination with human pluripotent stem cells generates a broad spectrum of immunological and clinical responses against colon cancer. Stem Cells *27*, 3103–3111.

Lowenfels, A.B., Maisonneuve, P., DiMagno, E.P., Elitsur, Y., Gates, L.K., Perrault, J., and Whitcomb, D.C. (1997). Hereditary pancreatitis and the risk of pancreatic cancer. International Hereditary Pancreatitis Study Group. J. Natl. Cancer Inst. *89*, 442–446.

Ouyang, X., Telli, M.L., and Wu, J.C. (2019). Induced pluripotent stem cell-based cancer vaccines. Front. Immunol. *10*, 1510.

Sakaguchi, S. (2005). Naturally arising Foxp3-expressing CD25+ CD4+ regulatory T cells in immunological tolerance to self and non-self. Nat. Immunol. *6*, 345–352.

Sherman, M., Peltekian, K.M., and Lee, C. (1995). Screening for hepatocellular carcinoma in chronic carriers of hepatitis B virus: incidence and prevalence of hepatocellular carcinoma in a North American urban population. Hepatology *22*, 432–438.

Siegel, R.L., Miller, K.D., and Jemal, A. (2019). Cancer Statistics, 2019. CA Cancer J. Clin. *69*, 7–34.

Torphy, R.J., Zhu, Y., and Schulick, R.D. (2018). Immunotherapy for pancreatic cancer: barriers and breakthroughs. Ann. Gastroenterol. Surg. *2*, 274–281.

Vansteenkiste, J.F., Cho, B.C., Vanakesa, T., De Pas, T., Zielinski, M., Kim, M.S., Jassem, J., Yoshimura, M., Dahabreh, J., Nakayama, H., et al. (2016). Efficacy of the MAGE-A3 cancer immunotherapeutic as adjuvant therapy in patients with resected MAGE-A3-positive non-small-cell lung cancer (MAGRIT): a randomised, double-blind, placebo-controlled, phase 3 trial. Lancet Oncol. *17*, 822–835.

Wang, L., Pegram, M.D., and Wu, J.C. (2019). Induced pluripotent stem cells as a novel cancer vaccine. Expert Opin. Biol. Ther. *19*, 1191–1197.

Wang, J., Shao, L., Wu, L., Ma, W., Zheng, Y., Hu, C., and Li, F. (2020). Expression levels of a gene signature in hiPSC associated with lung adenocarcinoma stem cells and its capability in eliciting specific antitumor immune-response in a humanized mice model. Thoracic Cancer *11*, 1603–1612.

Weiss, F.U. (2014). Pancreatic cancer risk in hereditary pancreatitis. Front. Physiol. *5*, 70.

Witkiewicz, A.K., McMillan, E.A., Balaji, U., Baek, G., Lin, W.-C., Mansour, J., Mollaee, M., Wagner, K.-U., Koduru, P., Yopp, A., et al. (2015). Whole-exome sequencing of pancreatic cancer defines genetic diversity and therapeutic targets. Nat. Commun. *6*, 1–11.

Yaddanapudi, K., Mitchell, R.A., Putty, K., Willer, S., Sharma, R.K., Yan, J., Bodduluri, H., and Eaton, J.W. (2012). Vaccination with Embryonic Stem Cells Protects against Lung Cancer: Is a Broad-Spectrum Prophylactic Vaccine against Cancer Possible? PLoS One *7*.

Yarchoan, M., Hopkins, A., and Jaffee, E.M. (2017). Tumor mutational burden and response rate to PD-1 inhibition. N. Engl. J. Med. *377*, 2500–2501.

Zhang, Z.-J., Chen, X.-H., Chang, X.-H., Ye, X., Li, Y., and Cui, H. (2012). Human embryonic stem cells–a potential vaccine for ovarian cancer. Asian Pac. J. Cancer Prev. *13*, 4295–4300.

Stem Cell Reports, Volume *16*

Supplemental Information

Antitumor effects of iPSC-based cancer vaccine in pancreatic cancer

Xiaoming Ouyang, Yu Liu, Yang Zhou, Jing Guo, Tzu-Tang Wei, Chun Liu, Bomi Lee, Binbin Chen, Angela Zhang, Kerriann M. Casey, Lin Wang, Nigel G. Kooreman, Aida Habtezion, Edgar G. Engleman, and Joseph C. Wu

SUPPLEMENTAL MATERIALS

Antitumor Effects of iPSC-Based Cancer Vaccine in Pancreatic Cancer

Xiaoming Ouyang, PhD[1,2], Yu Liu, PhD[1,2], Yang Zhou, MS[1,2], Jing Guo, PhD[3], Tzu-Tang Wei, PhD[1,2], Chun Liu, PhD[1,2], Bomi Lee, PhD[4], Binbin Chen, PhD[5], Angela Zhang, BS[1,2], Kerriann M. Casey, DVM, DACVP[6], Lin Wang, PhD[1,2], Nigel G. Kooreman, MD, PhD[8], Aida Habtezion, MD, MSc[4], Edgar G. Engleman, MD[7*], Joseph C. Wu, MD, PhD[1,2*]

[1]Stanford Cardiovascular Institute, [2]Department of Medicine, Division of Cardiovascular Medicine, Stanford University, [3]Department of Microbiology and Immunology, [4]Department of Medicine, Division of Gastroenterology & Hepatology, [5]Department of Genetics, [6]Department of Comparative Medicine, [7]Department of Pathology, Stanford University, Stanford, CA 94305, USA, [8]Department of Surgery, Leiden University Medical Center, Leiden, ZA 2333, the Netherlands

Supplemental Experimental Procedures

Generation of murine iPSCs. Murine iPSCs from C57BL/6J mice were generated as previously described (Kooreman et al., 2018). Briefly, fibroblasts from C57BL/6J (The Jackson Laboratory) were transfected with a Neon transfection system (ThermoFisher Scientific) with a codon-optimized mini-intronic plasmid (coMIP) (Diecke et al., 2015). After transfection, cells were cultured on irradiated mouse embryonic feeder cells in DMEM with 15% FBS, MEM Non-Essential Amino Acids (Gibco), and 10 ng/ml murine leukemia inhibiting factor (mLIF; EMD Millipore). Murine iPSC colonies were manually picked and allowed to grow for a few passages, followed by sorting for SSEA-1 using magnetic bead sorting (Miltenyi) to obtain a pure pluripotent population.

Maintenance of murine iPSC line. Murine C57BL/6J iPSC line was maintained in the MEK inhibition (MEKi) and GSK3 inhibition (GSK3i) with leukemia inhibitory factor condition without feeder cells in KnockOut™ DMEM (Gibco) with 15% KnockOut™ Serum Replacement (Gibco), 2 mM GlutaMax, Non-essential Amino Acids (Gibco), 100 µM ß-mercaptoethanol (SigmaAldrich), 0.5 µM ERK-Inhibitor PD0325901 (Selleck Chemicals), 3 µM GSK-3α/ß inhibitor CHIR99021 (Selleck Chemicals), and 10 ng/ml LIF (Millipore) (Silva et al., 2008). Cells were tested for pluripotent marker expression and were tested negative for mycoplasma contamination using the MycoAlert Detection Kit (Lonza). Before use, cells were sorted for SSEA-1 using magnetic bead sorting (Miltenyi) to obtain an enriched pluripotent population.

Histopathology of tumors. The tumors were explanted from mice and processed for histopathology at time of sacrifice. Briefly, the organs were fixed in 4% paraformaldehyde for 72 hr and transferred to 70% ethanol. Fixed samples were embedded in paraffin and sections were cut and stained with hematoxylin and eosin (H&E) for histological analysis by an expert veterinary pathologist (K.M.C).

Cytometry by Time-of-Flight (CyTOF). Immune cells were isolated from tumor draining lymph nodes (TDLNs) and dissociated into a single-cell suspension by pressing the tissue with the plunger of a 3 ml-syringe against a 70 µm strainer. ACK lysing buffer (Quality Biological) was used to remove any red blood cells. Cells were stimulated with PMA/ionomycin for 4 hr together with Brefeldin A. After stimulation, cells were stained with the maxpar mouse spleen/lymph node phenotyping kit (Fluidigm), as well as the maxpar mouse intracellular cytokine I panel kit (Fluidigm) plus anti-mouse FOXP3 and anti-mouse Ki67 antibodies conjugated in-house, and the viability dye Cisplatin (Fluidigm) using the FOXP3/Transcription factor staining buffer set (eBioscience) according to the manufacturer's instructions. Cells were resuspended in MaxPar water at a concentration of $1x10^5$-$1x10^7$ cells/mL with the addition of normalization beads and run on a CyTOF2 (Fluidigm) machine. The resulting data were normalized using the normalization beads. The data were analyzed using Cytobank online software (Kotecha et al., 2010) for viSNE (Amir et al., 2013), FLowSOM (Gassen et al., 2015) and SPADE (Qiu et al., 2011).

IgG binding assay. Cells were washed twice with PBS and resuspended in 100 µl FACS buffer with the addition of 2 µl of serum from the C+I vaccine or PBS treated mice and incubated for

30 min on 4°C. Following incubation, cells were washed twice and incubated with an Anti-IgG (minimal x-reactivity) Goat Polyclonal Antibody (Alexa Fluor® 594) [clone: Poly4053] (BioLegend) for another 20 min on 4°C. Unstained cells were included as negative controls. The cells were then analyzed using the BD FACSymphony™ Flow Cytometer.

Flow cytometry. For the immune profiling of immune cells in the spleen, splenocytes were isolated and incubated with ACK lysing buffer (Quality Biological) to remove red blood cells. Cytokine secretion were blocked for 4 hr by Brefeldin A. Cells were incubated with Panc02 cell lysate or PBS. After incubation, cells were stained with antibodies for surface markers containing CD3 (BioLegend Cat# 100231), CD4 (BioLegend Cat# 116022), CD8a (BioLegend Cat# 100734), CD45 (BioLegend Cat# 103137), and the intracellular markers IL-2 (BD Pharmingen), Granzyme-B (eBioscience), TNF-α (BD Pharmingen), and IFN-γ (Biolegend). A fixable viability dye LIVE/DEAD™ Fixable Near-IR Stain (Thermofisher) was added to exclude dead cells from the analysis. Cells were stained using the FOXP3/Transcription factor staining buffer set (eBioscience) according to the manufacturer's instructions. Samples were analyzed on the LSR-II Flow Cytometer analyzer in the Stanford Shared FACS Facility.

TCGA data sets. Publicly available TCGA patient data sets were used to analyze the percentages of genomic and transcriptomic alterations in cancer-iPSC signature genes in human tumors of major cancer types. The patient cohorts include pancreatic cancer (Hoadley et al., 2018; Witkiewicz et al., 2015), melanoma (Hoadley et al., 2018), esophagogastric cancer (Hoadley et al., 2018), non-small cell lung cancer (Campbell et al., 2016), ovarian epithelial tumor (Hoadley et al., 2018), head and neck cancer (Hoadley et al., 2018), invasive breast

carcinoma (Ciriello et al., 2015), hepatocellular carcinoma (Hoadley et al., 2018), prostate cancer (Armenia et al., 2018), colorectal adenocarcinoma (Hoadley et al., 2018), glioma (Brennan et al., 2013), and germ cell tumor (Bagrodia et al., 2016).

TCGA data analysis using the cBioPortal and R. Publicly available TCGA patient data sets were used to analyze the percentages of transcriptomic alterations in cancer-iPSC signature genes in human tumors of major cancer types. The cBioPortal (http://cbioportal.org) tool (Cerami et al., 2012; Gao et al., 2013) was used for analyzing TCGA data. The patient survival information with or without mRNA overexpression in iPSC-cancer signature genes was queried using cBioportal analysis on TCGA's PanCancer Atlas cohorts. To determine the enrichment scores of the iPSC-cancer signature genes in TCGA tumors, we performed a single-sample Gene Set Enrichment Analysis (ssGSEA) using the GSVA package in R. TCGA RNA sequencing data of tumor samples were downloaded from https://portal.gdc.cancer.gov/.

RNA sequencing data analysis. For RNA-seq data, quality was examined by way of analyzing per base sequence quality plots using FastQC. The trimming of sequence reads was done by TrimGalore. RNA-seq reads were aligned to the mouse genome (mm10) using the STAR software (Dobin et al., 2013). Reads that overlapped with exon coordinates were counted using RSEM and feautrecounts (Li and Dewey, 2011; Liao et al., 2014). Raw read counts were transformed using the variance stabilizing transformation (VST) function included in the DESeq2 R package. Mean and standard deviations of normalized expressions were calculated for each gene. Z-scores were determined by subtracting the mean from each expression value and dividing by the standard deviation. Differentially expressed genes (DEG) between different

groups were identified using DESeq2 R package (Anders and Huber, 2010). Genes with a Benjamin-Hochberg corrected p< 0.05 were considered significant. The RNA-seq expression data sets were downloaded from GEO projects (GEO: GSE157185, GSE160434, GSM4077903 and GSE36294).

Gene Set Enrichment Analysis (GSEA). We utilized the Software "GSEA 4.1.0" (Subramanian et al., 2005) to perform GSEA analysis of iPSC-cancer signature genes as a user-defined gene set with Panc02, mouse iPSCs, and mouse embryonic fibroblasts (MEF) RNA-seq expression data. FDR<0.001 and P < 0.001 was regarded as statistically significant.

Peptide immunogenicity prediction. Immunogenic peptides in iPSC-cancer signature gene were predicted with machine learning algorithms NetMHCpan4.0 and MARIA to identify T cell epitopes highly presentable by HLA alleles (human MHC I and II). For NetMHCpan4.0, we scanned 9-mer peptides in the iPSC-cancer signature genes, and for MARIA we scanned 15-mer peptides. We ranked candidate T cell epitopes by binding affinity and selected epitopes that are presentable by more than 50% of inputted common MHC I alleles and 100% of inputted common MHC II alleles among the US population. We validated the prediction by searching these top candidates with 90% sequence similarity in The Immune Epitope Database (IEDB).

Quantification and statistical analyses. All values in bar graphs and curves are expressed as means ± SEM. Intergroup differences were appropriately assessed by either unpaired two-tailed Student's t-test or one-way analysis of variance (ANOVA) with multiple comparison tests using PRISM GraphPad software. $^{*}P < 0.05$, $^{**}P < 0.01$, $^{***}P < 0.001$, $^{****}P < 0.0001$.

Supplementary Figure Legend

Supplementary Figure 1. H&E staining of mouse tumors confirmed tumor morphology. H&E staining showing histology of mouse tumors from mice treated with PBS, iPSC alone, CpG alone, and the C+I vaccine. NT, no tumors.

Supplementary Figure 2. FlowSOM results for one representative mouse treated with PBS or the C+I vaccine as Minimum Spanning Tree (MST) grid. (**A**) Heatmap representing the expression of the indicated markers within the metaclusters. (**B-C**) FlowSOM was performed using 225 clusters and 10 metaclusters. Each cluster was represented by one pie chart, and metaclusters were denoted by background shading. Major differences observed in metaclusters 10 and 6 between PBS and the C+I vaccine treated mice are outlined in dark blue ($CD8^+$ T cells) and light blue ($CD4^+FOXP3^+$ Treg cells).

Supplementary Figure 3. The C+I vaccine increases serum IgG binding to miPSCs and cancer cells, increases recruitment of plasmocytic dendric cells (pDCs) in tumor draining lymph nodes, and stimulates cancer cell specific IFNγ+ cytotoxic T cells in spleen, but does not increase systemic cytokine levels nor affect mouse body weight production compared to PBS controls. (A) Increased percentage of serum IgG binding to miPSCs and murine PDAC cancer cells from C+I vaccine treated mice compared to PBS treated controls, without a significant increase in non-specific fibroblast binding (*n*=3, mean ± SEM, Student's t-test). **(B)** Percentage of pDCs ($CD45^+CD11c^+NK1.1^-B220^+CD11b^-$) among $CD45^+CD19^-NK1.1^-CD11c^+$ cells in tumor draining lymph nodes from PBS or C+I vaccine treated mice (*n*=3, mean ± SEM, Student's t-test). (**C**) The C+I vaccine stimulates cancer cell specific IFNγ+ cytotoxic T cells in

spleen upon re-exposure to cancer cell lysate compared to PBS controls. Quantification of frequencies of $CD8^+$ IFNγ+ cytotoxic lymphocytes in mouse spleens from C+I vaccine or PBS control treated mice upon exposure to Panc02 lysate or PBS (n=3-4, mean ± SEM, *$P < 0.05$, Student's t-test). **(D)** Quantification of frequencies of $CD3^+$Granzyme B^+, IL-2, or $IFN\gamma^+$ lymphocytes in mouse spleens (n=3-4, means ± SEM, *$P < 0.05$, Tukey's multiple comparisons test). (**E**) No significant difference among different groups in terms of mouse body weights (g) was observed. (n=7-8, mean ± SEM, n.s., not significant, Tukey's multiple comparisons test).

Supplementary Figure 4. Shared upregulated genes in mouse and human iPSCs and pancreatic cancer cell lines. **(A)** Heatmap showing the hierarchical clustering of upregulated genes in mouse iPSC (miPSC.37_rep1, miPSC.37_rep2, miPSC.42_rep1, miPSC.42_rep2), mouse pancreatic cancer cell line Panc02 cells (mPanc02_SY4 and mPanc02_SY13), human iPSC clones (hiPSC_rep1 and hiPSC_rep2), human pancreatic cancer cell lines (hPan_Capan1, hPan_BxPC3, hPan_Aspc1, and hPan_Panc1) compared to mouse embryonic fibroblasts (MEF_rep1 and MEF_rep2) and human adult fibroblasts (hFB_rep1 and hFB_rep1). (**B-C**) Enrichment plots by gene set enrichment analysis (GSEA) for the iPSC cancer gene signature in Panc02 cells and murine iPSCs (miPSCs). iPSC cancer gene signature is significantly enriched in both Panc02 cells and miPSCs, while negatively corelated to mouse embryonic fibroblasts (MEFs). Normalized enrichment scores (NESs), false discovery rate (FDR) q values, and P-values were computed by GSEA.

Supplemental Tables

Supplementary Table 1. iPSC-cancer signature gene table.

Supplementary Table 2. iPSC-cancer signature genes in immune system related pathways.

Supplementary Table 3. Immunogenic iPSC-cancer signature genes and peptides.

References

Amir, E.D., Davis, K.L., Tadmor, M.D., Simonds, E.F., Levine, J.H., Bendall, S.C., Shenfeld, D.K., Krishnaswamy, S., Nolan, G.P., and Pe'er, D. (2013). viSNE enables visualization of high dimensional single-cell data and reveals phenotypic heterogeneity of leukemia. Nat Biotechnol *31*, 545–552.

Anders, S., and Huber, W. (2010). Differential expression analysis for sequence count data. Genome Biology *11*, R106.

Armenia, J., Wankowicz, S.A.M., Liu, D., Gao, J., Kundra, R., Reznik, E., Chatila, W.K., Chakravarty, D., Han, G.C., Coleman, I., et al. (2018). The long tail of oncogenic drivers in prostate cancer. Nat. Genet. *50*, 645–651.

Bagrodia, A., Lee, B.H., Lee, W., Cha, E.K., Sfakianos, J.P., Iyer, G., Pietzak, E.J., Gao, S.P., Zabor, E.C., Ostrovnaya, I., et al. (2016). Genetic determinants of cisplatin resistance in patients with advanced germ cell tumors. J. Clin. Oncol. *34*, 4000–4007.

Brennan, C.W., Verhaak, R.G.W., McKenna, A., Campos, B., Noushmehr, H., Salama, S.R., Zheng, S., Chakravarty, D., Sanborn, J.Z., Berman, S.H., et al. (2013). The somatic genomic landscape of glioblastoma. Cell *155*, 462–477.

Campbell, J.D., Alexandrov, A., Kim, J., Wala, J., Berger, A.H., Pedamallu, C.S., Shukla, S.A., Guo, G., Brooks, A.N., Murray, B.A., et al. (2016). Distinct patterns of somatic genome alterations in lung adenocarcinomas and squamous cell carcinomas. Nat. Genet. *48*, 607–616.

Cerami, E., Gao, J., Dogrusoz, U., Gross, B.E., Sumer, S.O., Aksoy, B.A., Jacobsen, A., Byrne, C.J., Heuer, M.L., Larsson, E., et al. (2012). The cBio cancer genomics portal: an Open platform for exploring multidimensional cancer genomics data. Cancer Discov *2*, 401–404.

Ciriello, G., Gatza, M.L., Beck, A.H., Wilkerson, M.D., Rhie, S.K., Pastore, A., Zhang, H., McLellan, M., Yau, C., Kandoth, C., et al. (2015). Comprehensive molecular portraits of invasive lobular breast cancer. Cell *163*, 506–519.

Diecke, S., Lu, J., Lee, J., Termglinchan, V., Kooreman, N.G., Burridge, P.W., Ebert, A.D., Churko, J.M., Sharma, A., Kay, M.A., et al. (2015). Novel codon-optimized mini-intronic plasmid for efficient, inexpensive, and xeno-free induction of pluripotency. Sci Rep *5*.

Dobin, A., Davis, C.A., Schlesinger, F., Drenkow, J., Zaleski, C., Jha, S., Batut, P., Chaisson, M., and Gingeras, T.R. (2013). STAR: ultrafast universal RNA-seq aligner. Bioinformatics *29*, 15–21.

Gao, J., Aksoy, B.A., Dogrusoz, U., Dresdner, G., Gross, B., Sumer, S.O., Sun, Y., Jacobsen, A., Sinha, R., Larsson, E., et al. (2013). Integrative analysis of complex cancer genomics and clinical profiles using the cBioPortal. Sci Signal *6*, pl1.

Gassen, S.V., Callebaut, B., Helden, M.J.V., Lambrecht, B.N., Demeester, P., Dhaene, T., and Saeys, Y. (2015). FlowSOM: Using self-organizing maps for visualization and interpretation of cytometry data. Cytometry Part A *87*, 636–645.

Hoadley, K.A., Yau, C., Hinoue, T., Wolf, D.M., Lazar, A.J., Drill, E., Shen, R., Taylor, A.M., Cherniack, A.D., Thorsson, V., et al. (2018). Cell-of-origin patterns dominate the molecular classification of 10,000 tumors from 33 types of cancer. Cell *173*, 291-304.e6.

Kooreman, N.G., Kim, Y., de Almeida, P.E., Termglinchan, V., Diecke, S., Shao, N.-Y., Wei, T.-T., Yi, H., Dey, D., Nelakanti, R., et al. (2018). Autologous iPSC-based vaccines elicit anti-tumor responses in vivo. Cell Stem Cell *22*, 501-513.e7.

Kotecha, N., Krutzik, P.O., and Irish, J.M. (2010). Web-based analysis and publication of flow cytometry experiments. Curr Protoc Cytom *Chapter 10*, Unit10.17.

Li, B., and Dewey, C.N. (2011). RSEM: accurate transcript quantification from RNA-Seq data with or without a reference genome. BMC Bioinformatics *12*, 323.

Liao, Y., Smyth, G.K., and Shi, W. (2014). featureCounts: an efficient general purpose program for assigning sequence reads to genomic features. Bioinformatics *30*, 923–930.

Qiu, P., Simonds, E.F., Bendall, S.C., Gibbs, K.D., Bruggner, R.V., Linderman, M.D., Sachs, K., Nolan, G.P., and Plevritis, S.K. (2011). Extracting a cellular hierarchy from high-dimensional cytometry data with SPADE. Nat. Biotechnol. *29*, 886–891.

Silva, J., Barrandon, O., Nichols, J., Kawaguchi, J., Theunissen, T.W., and Smith, A. (2008). Promotion of reprogramming to ground state pluripotency by signal inhibition. PLoS Biol. *6*, e253.

Subramanian, A., Tamayo, P., Mootha, V.K., Mukherjee, S., Ebert, B.L., Gillette, M.A., Paulovich, A., Pomeroy, S.L., Golub, T.R., Lander, E.S., et al. (2005). Gene set enrichment analysis: a knowledge-based approach for interpreting genome-wide expression profiles. Proc Natl Acad Sci U S A *102*, 15545–15550.

Witkiewicz, A.K., McMillan, E.A., Balaji, U., Baek, G., Lin, W.-C., Mansour, J., Mollaee, M., Wagner, K.-U., Koduru, P., Yopp, A., et al. (2015). Whole-exome sequencing of pancreatic cancer defines genetic diversity and therapeutic targets. Nature Communications *6*, 1–11.

Supplementary Figure 1

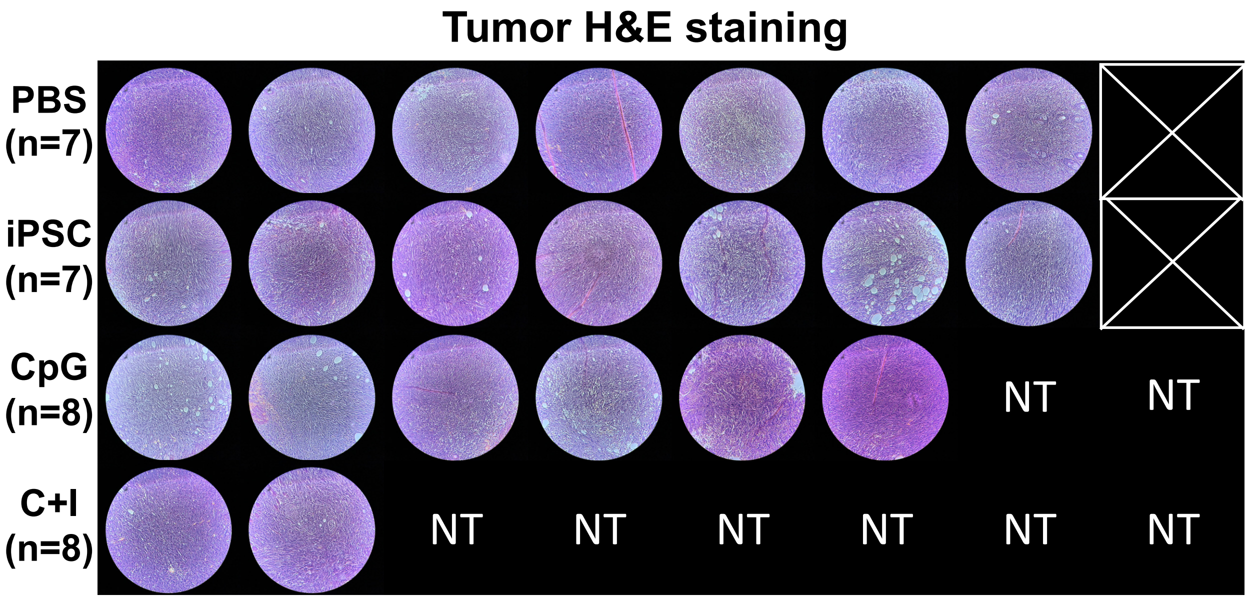

NT , No Tumors.

Supplementary Figure 2

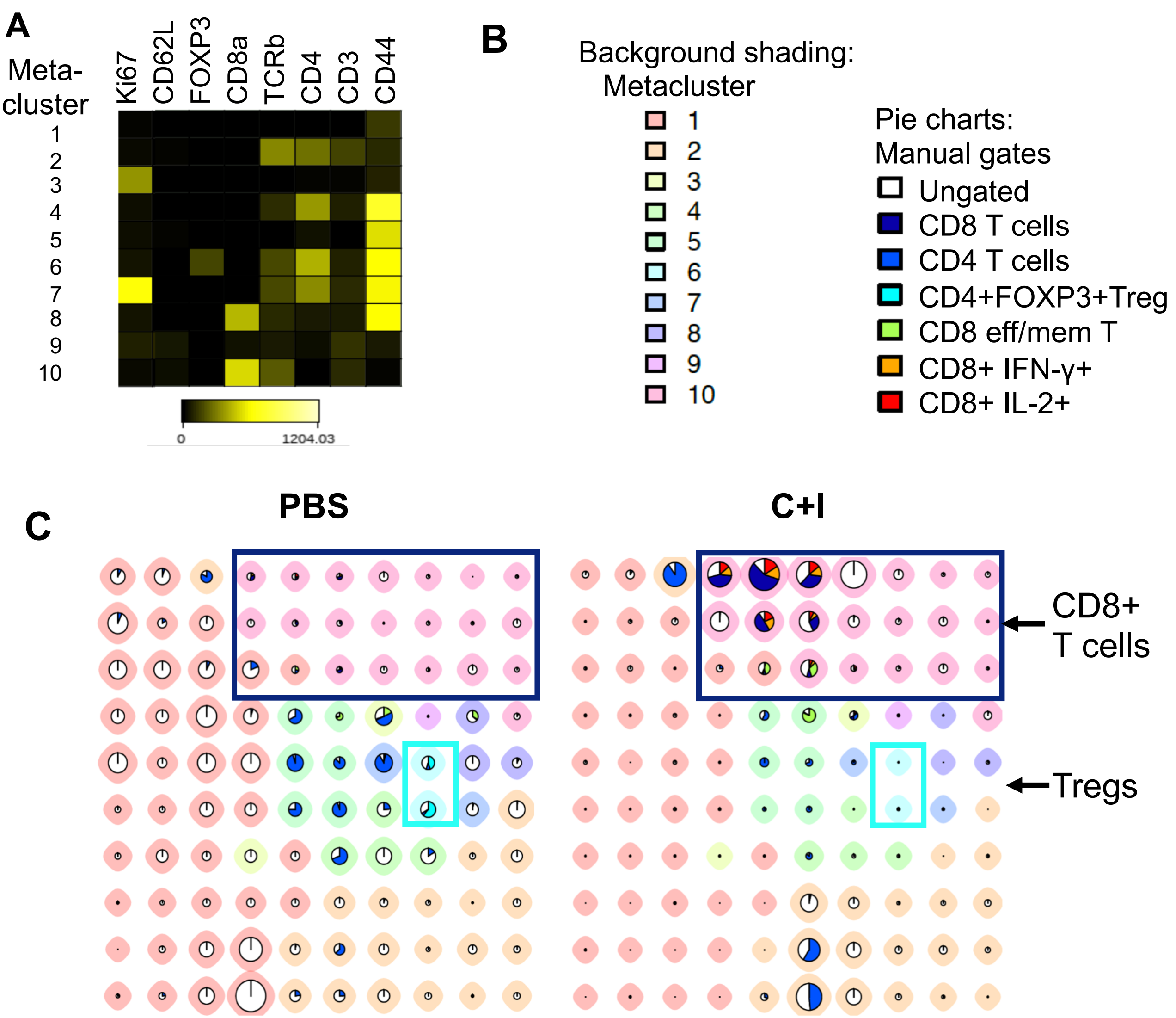

Supplementary Figure 3

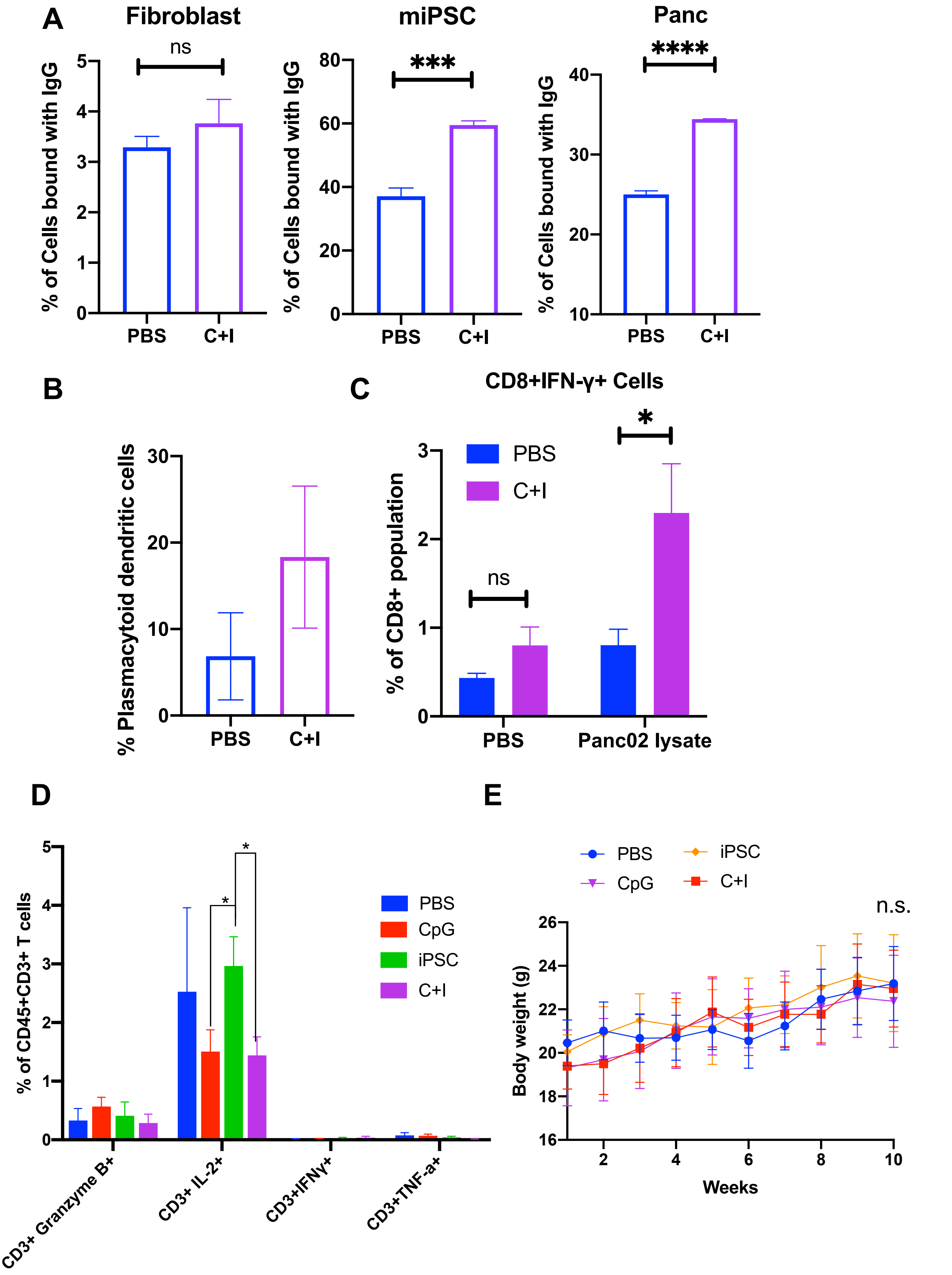

Supplementary Figure 4

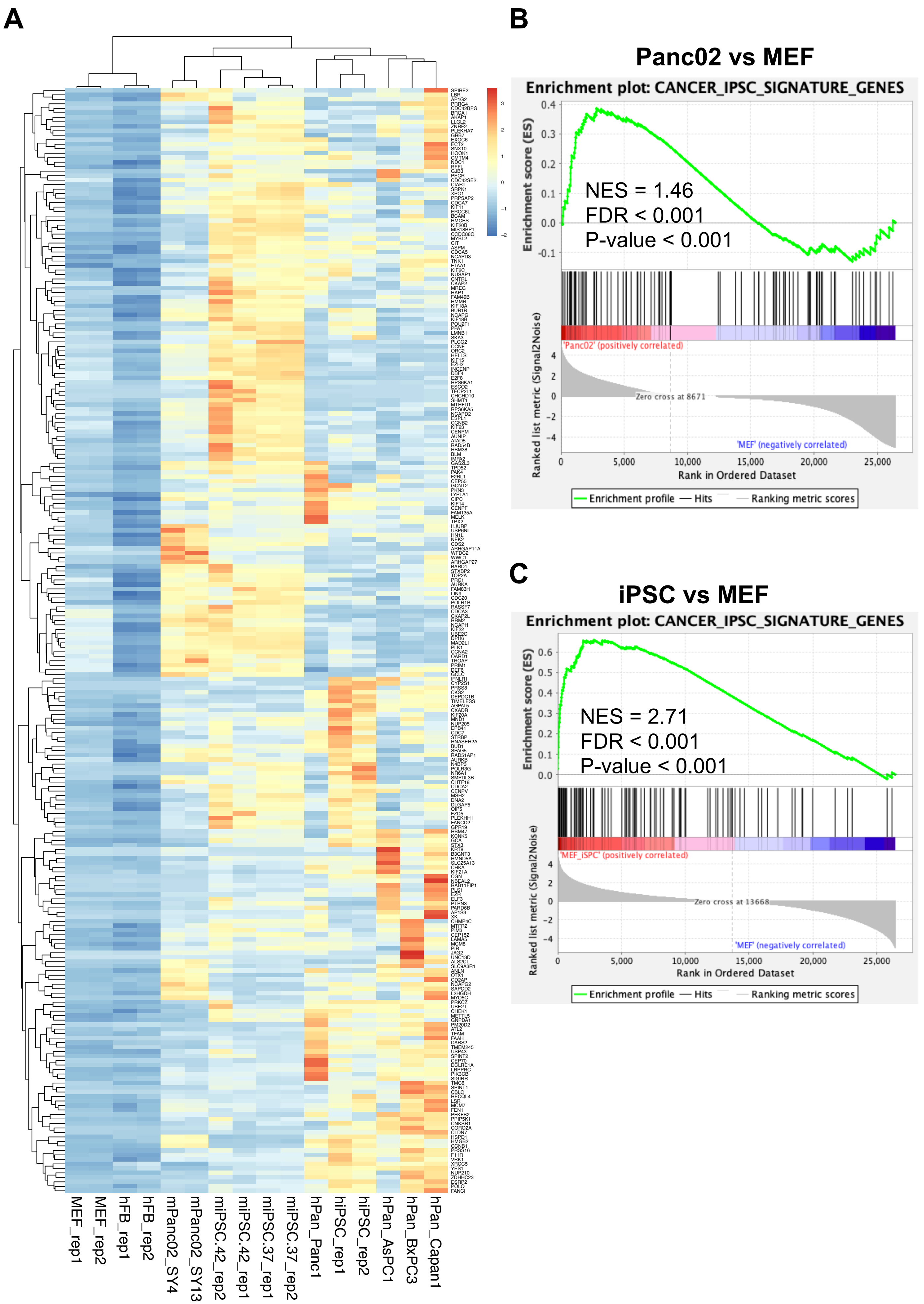

Table S1. iPSC-Cancer Signature Genes

Gene ID	Gene Symbol	Gene Family	Protein Class
HUMAN|HGNC=370|UniProtKB=Q02952	*AKAP12*	A-kinase anchor protein 12;	
HUMAN|HGNC=14082|UniProtKB=Q9NQW6	*ANLN*	Anillin;	
HUMAN|HGNC=11110|UniProtKB=O14497	*ARID1A*	AT-rich interactive domain-containing protein 1A;	
HUMAN|HGNC=19048|UniProtKB=Q8IZT6	*ASPM*	Abnormal spindle-like microcephaly-associated protein;	
HUMAN|HGNC=18318|UniProtKB=Q8IXJ9	*ASXL1*	Putative Polycomb group protein ASXL1;	
HUMAN|HGNC=794|UniProtKB=P31939	*ATIC*	Bifunctional purine biosynthesis protein PURH;	hydrolase(PC00121);methyltransferase(PC00155)
HUMAN|HGNC=11393|UniProtKB=O14965	*AURKA*	Aurora kinase A;	non-receptor serine/threonine protein kinase(PC00167)
HUMAN|HGNC=952|UniProtKB=Q99728	*BARD1*	BRCA1-associated RING domain protein 1;	
HUMAN|HGNC=14347|UniProtKB=Q9H6U6	*BCAS3*	Breast carcinoma-amplified sequence 3;	
HUMAN|HGNC=20893|UniProtKB=Q6W2J9	*BCOR*	BCL-6 corepressor;	
HUMAN|HGNC=1058|UniProtKB=P54132	*BLM*	Bloom syndrome protein;	
HUMAN|HGNC=1100|UniProtKB=P38398	*BRCA1*	Breast cancer type 1 susceptibility protein;	ubiquitin-protein ligase(PC00234)
HUMAN|HGNC=1101|UniProtKB=P51587	*BRCA2*	Breast cancer type 2 susceptibility protein;	damaged DNA-binding protein(PC00086)
HUMAN|HGNC=1103|UniProtKB=P25440	*BRD2*	Bromodomain-containing protein 2;	
HUMAN|HGNC=1104|UniProtKB=Q15059	*BRD3*	Bromodomain-containing protein 3;	
HUMAN|HGNC=13575|UniProtKB=O60885	*BRD4*	Bromodomain-containing protein 4;	
HUMAN|HGNC=20473|UniProtKB=Q9BX63	*BRIP1*	Fanconi anemia group J protein;	DNA helicase(PC00011)
HUMAN|HGNC=1148|UniProtKB=O43683	*BUB1*	Mitotic checkpoint serine/threonine-protein kinase BUB1;	
HUMAN|HGNC=1149|UniProtKB=O60566	*BUB1B*	Mitotic checkpoint serine/threonine-protein kinase BUB1 beta;	
HUMAN|HGNC=1493|UniProtKB=P49589	*CARS*	Cysteine--tRNA ligase, cytoplasmic;	RNA binding protein(PC00031);aminoacyl-tRNA synthetase(PC00047)
HUMAN|HGNC=1578|UniProtKB=P20248	*CCNA2*	Cyclin-A2;	kinase activator(PC00138)
HUMAN|HGNC=1579|UniProtKB=P14635	*CCNB1*	G2/mitotic-specific cyclin-B1;	kinase activator(PC00138)
HUMAN|HGNC=19437|UniProtKB=Q9NPC3	*CCNB1IP1*	E3 ubiquitin-protein ligase CCNB1IP1;	
HUMAN|HGNC=1589|UniProtKB=P24864	*CCNE1*	G1/S-specific cyclin-E1;	kinase activator(PC00138)

HUMAN|HGNC=1591|UniProtKB=P41002	*CCNF*	Cyclin-F;	kinase activator(PC00138)
HUMAN|HGNC=1725|UniProtKB=P30304	*CDC25A*	M-phase inducer phosphatase 1;	protein phosphatase(PC00195)
HUMAN|HGNC=1727|UniProtKB=P30307	*CDC25C*	M-phase inducer phosphatase 3;	protein phosphatase(PC00195)
HUMAN|HGNC=1744|UniProtKB=Q99741	*CDC6*	Cell division control protein 6 homolog;	
HUMAN|HGNC=1722|UniProtKB=P06493	*CDK1*	Cyclin-dependent kinase 1;	non-receptor serine/threonine protein kinase(PC00167);non-receptor tyrosine protein kinase(PC00168)
HUMAN|HGNC=1729|UniProtKB=P21127	*CDK11B*	Cyclin-dependent kinase 11B;	non-receptor serine/threonine protein kinase(PC00167);non-receptor tyrosine protein kinase(PC00168)
HUMAN|HGNC=1772|UniProtKB=Q00526	*CDKN3*	Cyclin-dependent kinase 3;	non-receptor serine/threonine protein kinase(PC00167);non-receptor tyrosine protein kinase(PC00168)
HUMAN|HGNC=1857|UniProtKB=P49454	*CENPF*	Centromere protein F;	
HUMAN|HGNC=1917|UniProtKB=O14647	*CHD2*	Chromodomain-helicase-DNA-binding protein 2;	
HUMAN|HGNC=16627|UniProtKB=O96017	*CHEK2*	Serine/threonine-protein kinase Chk2;	
HUMAN|HGNC=1858|UniProtKB=Q7Z7A1	*CNTRL*	Centriolin;	
HUMAN|HGNC=2348|UniProtKB=Q92793	*CREBBP*	CREB-binding protein;	acetyltransferase(PC00038);chromatin/chromatin-binding protein(PC00077);transcription cofactor(PC00217)
HUMAN|HGNC=18677|UniProtKB=Q9NXZ2	*DDX43*	Probable ATP-dependent RNA helicase DDX43;	
HUMAN|HGNC=3115|UniProtKB=O00716	*E2F3*	Transcription factor E2F3;	nucleic acid binding(PC00171);transcription factor(PC00218)
HUMAN|HGNC=3155|UniProtKB=Q9H8V3	*ECT2*	Protein ECT2;	
HUMAN|HGNC=3279|UniProtKB=Q99613	*EIF3C*	Eukaryotic translation initiation factor 3 subunit C;	translation initiation factor(PC00224)
HUMAN|HGNC=1316|UniProtKB=Q9HC35	*EML4*	Echinoderm microtubule-associated protein-like 4;	
HUMAN|HGNC=3373|UniProtKB=Q09472	*EP300*	Histone acetyltransferase p300;	acetyltransferase(PC00038);chromatin/chromatin-binding protein(PC00077);transcription cofactor(PC00217)
HUMAN|HGNC=11958|UniProtKB=Q96L91	*EP400*	E1A-binding protein p400;	
HUMAN|HGNC=5174|UniProtKB=Q7Z444	*ERAS*	GTPase ERas;	small GTPase(PC00208)
HUMAN|HGNC=27234|UniProtKB=Q86X53	*ERICH1*	Glutamate-rich protein 1;	
HUMAN|HGNC=3494|UniProtKB=P41161	*ETV5*	ETS translocation variant 5;	nucleic acid binding(PC00171);signaling molecule(PC00207);winged helix/forkhead transcription factor(PC00246)

HUMAN|HGNC=3527|UniProtKB=Q15910	*EZH2*	Histone-lysine N-methyltransferase EZH2;	
HUMAN|HGNC=3585|UniProtKB=Q9BXW9	*FANCD2*	Fanconi anemia group D2 protein;	
HUMAN|HGNC=13584|UniProtKB=Q9UKT4	*FBXO5*	F-box only protein 5;	
HUMAN|HGNC=3682|UniProtKB=P08620	*FGF4*	Fibroblast growth factor 4;	growth factor(PC00112)
HUMAN|HGNC=3683|UniProtKB=P12034	*FGF5*	Fibroblast growth factor 5;	growth factor(PC00112)
HUMAN|HGNC=3686|UniProtKB=P55075	*FGF8*	Fibroblast growth factor 8;	growth factor(PC00112)
HUMAN|HGNC=19752|UniProtKB=Q8N3X1	*FNBP4*	Formin-binding protein 4;	
HUMAN|HGNC=3797|UniProtKB=P53539	*FOSB*	Protein fosB;	basic leucine zipper transcription factor(PC00056)
HUMAN|HGNC=3806|UniProtKB=O00358	*FOXE1*	Forkhead box protein E1;	DNA binding protein(PC00009);winged helix/forkhead transcription factor(PC00246)
HUMAN|HGNC=4066|UniProtKB=Q13480	*GAB1*	GRB2-associated-binding protein 1;	transmembrane receptor regulatory/adaptor protein(PC00226)
HUMAN|HGNC=26881|UniProtKB=Q17RS7	*GEN1*	Flap endonuclease GEN homolog 1;	damaged DNA-binding protein(PC00086);endodeoxyribonuclease(PC00093);exodeoxyribonuclease(PC00098);hydrolase(PC00121)
HUMAN|HGNC=5009|UniProtKB=P52926	*HMGA2*	High mobility group protein HMGI-C;	DNA binding protein(PC00009)
HUMAN|HGNC=5125|UniProtKB=P31276	*HOXC13*	Homeobox protein Hox-C13;	
HUMAN|HGNC=5258|UniProtKB=P08238	*HSP90AB1*	Heat shock protein HSP 90-beta;	Hsp90 family chaperone(PC00028)
HUMAN|HGNC=16389|UniProtKB=Q96EW2	*HSPBAP1*	HSPB1-associated protein 1;	
HUMAN|HGNC=17582|UniProtKB=Q8WYB5	*KAT6B*	Histone acetyltransferase KAT6B;	acetyltransferase(PC00038);chromatin/chromatin-binding protein(PC00077);zinc finger transcription factor(PC00244)
HUMAN|HGNC=13610|UniProtKB=Q8NHM5	*KDM2B*	Lysine-specific demethylase 2B;	
HUMAN|HGNC=23025|UniProtKB=Q8TD94	*KLF14*	Krueppel-like factor 14;	DNA binding protein(PC00009);transcription cofactor(PC00217);zinc finger transcription factor(PC00244)
HUMAN|HGNC=6763|UniProtKB=Q13257	*MAD2L1*	Mitotic spindle assembly checkpoint protein MAD2A;	
HUMAN|HGNC=6879|UniProtKB=Q16659	*MAPK6*	Mitogen-activated protein kinase 6;	non-receptor serine/threonine protein kinase(PC00167)
HUMAN|HGNC=6940|UniProtKB=P10911	*MCF2*	Proto-oncogene DBL;	signaling molecule(PC00207)
HUMAN|HGNC=6973|UniProtKB=Q00987	*MDM2*	E3 ubiquitin-protein ligase Mdm2;	chromatin/chromatin-binding protein(PC00077)
HUMAN|HGNC=13363|UniProtKB=Q9H2W2	*MIXL1*	Homeobox protein MIXL1;	

HUMAN|HGNC=7127|UniProtKB=P40692	*MLH1*	DNA mismatch repair protein Mlh1;	DNA binding protein(PC00009)
HUMAN|HGNC=7325|UniProtKB=P43246	*MSH2*	DNA mismatch repair protein Msh2;	DNA binding protein(PC00009)
HUMAN|HGNC=7329|UniProtKB=P52701	*MSH6*	DNA mismatch repair protein Msh6;	DNA binding protein(PC00009)
HUMAN|HGNC=23784|UniProtKB=Q9BTC8	*MTA3*	Metastasis-associated protein MTA3;	chromatin/chromatin-binding protein(PC00077);histone(PC00118)
HUMAN|HGNC=3942|UniProtKB=P42345	*MTOR*	Serine/threonine-protein kinase mTOR;	non-receptor serine/threonine protein kinase(PC00167);nucleic acid binding(PC00171);nucleotide kinase(PC00172)
HUMAN|HGNC=7547|UniProtKB=P10243	*MYBL1*	Myb-related protein A;	
HUMAN|HGNC=7559|UniProtKB=P04198	*MYCN*	N-myc proto-oncogene protein;	basic helix-loop-helix transcription factor(PC00055);nucleic acid binding(PC00171)
HUMAN|HGNC=7670|UniProtKB=Q9Y6Q9	*NCOA3*	Nuclear receptor coactivator 3;	acetyltransferase(PC00038);transcription factor(PC00218)
HUMAN|HGNC=7857|UniProtKB=P30419	*NMT1*	Glycylpeptide N-tetradecanoyltransferase 1;	transferase(PC00220)
HUMAN|HGNC=18016|UniProtKB=Q8WUM0	*NUP133*	Nuclear pore complex protein Nup133;	
HUMAN|HGNC=8522|UniProtKB=P32243	*OTX2*	Homeobox protein OTX2;	homeodomain transcription factor(PC00119)
HUMAN|HGNC=26144|UniProtKB=Q86YC2	*PALB2*	Partner and localizer of BRCA2;	
HUMAN|HGNC=8729|UniProtKB=P12004	*PCNA*	Proliferating cell nuclear antigen;	DNA polymerase processivity factor(PC00015)
HUMAN|HGNC=14005|UniProtKB=Q86TG7	*PEG10*	Retrotransposon-derived protein PEG10;	
HUMAN|HGNC=8987|UniProtKB=Q9P1W9	*PIM2*	Serine/threonine-protein kinase pim-2;	serine/threonine protein kinase receptor(PC00205)
HUMAN|HGNC=9045|UniProtKB=Q6DJT9	*PLAG1*	Zinc finger protein PLAG1;	KRAB box transcription factor(PC00029)
HUMAN|HGNC=9221|UniProtKB=Q01860	*POU5F1*	POU domain, class 5, transcription factor 1;	
HUMAN|HGNC=9347|UniProtKB=Q13029	*PRDM2*	PR domain zinc finger protein 2;	
HUMAN|HGNC=9822|UniProtKB=O15315	*RAD51B*	DNA repair protein RAD51 homolog 2;	
HUMAN|HGNC=14428|UniProtKB=Q9H2T7	*RANBP17*	Ran-binding protein 17;	transfer/carrier protein(PC00219)
HUMAN|HGNC=9965|UniProtKB=Q9P2R6	*RERE*	Arginine-glutamic acid dipeptide repeats protein;	
HUMAN|HGNC=9966|UniProtKB=Q13127	*REST*	RE1-silencing transcription factor;	KRAB box transcription factor(PC00029)
HUMAN|HGNC=10315|UniProtKB=P35268	*RPL22*	60S ribosomal protein L22;	ribosomal protein(PC00202)
HUMAN|HGNC=10451|UniProtKB=P23921	*RRM1*	Ribonucleoside-diphosphate reductase large subunit;	reductase(PC00198)
HUMAN|HGNC=20256|UniProtKB=Q5VUG0	*SFMBT2*	Scm-like with four MBT domains protein 2;	chromatin/chromatin-binding protein(PC00077);transcription factor(PC00218)

HUMAN|HGNC=10901|UniProtKB=Q13309	*SKP2*	S-phase kinase-associated protein 2;	
HUMAN|HGNC=11195|UniProtKB=P48431	*SOX2*	Transcription factor SOX-2;	HMG box transcription factor(PC00024)
HUMAN|HGNC=11199|UniProtKB=P41225	*SOX3*	Transcription factor SOX-3;	HMG box transcription factor(PC00024)
HUMAN|HGNC=10879|UniProtKB=Q15468	*STIL*	SCL-interrupting locus protein;	
HUMAN|HGNC=11524|UniProtKB=Q9Y6A5	*TACC3*	Transforming acidic coiled-coil-containing protein 3;	
HUMAN|HGNC=11648|UniProtKB=P56279	*TCL1A*	T-cell leukemia/lymphoma protein 1A;	
HUMAN|HGNC=11701|UniProtKB=P13385	*TDGF1*	Teratocarcinoma-derived growth factor 1;	calcium-binding protein(PC00060)
HUMAN|HGNC=29484|UniProtKB=Q8NFU7	*TET1*	Methylcytosine dioxygenase TET1;	
HUMAN|HGNC=11752|UniProtKB=P19532	*TFE3*	Transcription factor E3;	
HUMAN|HGNC=11805|UniProtKB=Q13009	*TIAM1*	T-lymphoma invasion and metastasis-inducing protein 1;	
HUMAN|HGNC=11989|UniProtKB=P11388	*TOP2A*	DNA topoisomerase 2-alpha;	
HUMAN|HGNC=7146|UniProtKB=Q7Z4N2	*TRPM1*	Transient receptor potential cation channel subfamily M member 1;	ion channel(PC00133);receptor(PC00197)
HUMAN|HGNC=20071|UniProtKB=Q53GS9	*USP39*	U4/U6.U5 tri-snRNP-associated protein 2;	cysteine protease(PC00081)
HUMAN|HGNC=12718|UniProtKB=Q99986	*VRK1*	Serine/threonine-protein kinase VRK1;	non-receptor serine/threonine protein kinase(PC00167)
HUMAN|HGNC=8014|UniProtKB=P67809	*YBX1*	Nuclease-sensitive element-binding protein 1;	
HUMAN|HGNC=9397|UniProtKB=Q9ULU4	*ZMYND8*	Protein kinase C-binding protein 1;	
HUMAN|HGNC=13099|UniProtKB=P13682	*ZNF35*	Zinc finger protein 35;	KRAB box transcription factor(PC00029)

Table S2. iPSC-Cancer Signature Genes In Immune System Related Pathways

Pathway name	# of genes found	# of total gene in the pathway	Species name	Genes found in pathway
Adaptive Immune System	4	1003	Homo sapiens	ASXL1; CCNF; SKP2; MTOR
Cytokine Signaling in Immune system	3	1108	Homo sapiens	SOX2; NUP133; MTOR
Innate Immune System	4	1331	Homo sapiens	CREBBP; ATIC; HSP90AB1; EP300
Immune System	10	2713	Homo sapiens	SOX2; ASXL1; CREBBP; ATIC; HSP90AB1; NUP133; CCNF; EP300; SKP2; MTOR

Table S3. Immunogenic iPSC-Cancer Signature Genes and Peptides

Gene ID	MHC I Peptide	MHC II Peptide	Position	MHC-I Coverage %	MHC-II Coverage %	IEDB Evidence Number
ASPM	MIIAVTSYK	QSRIRMIIAVTSYKR	1382	55	100	1
	RMHRLHMRY	IQSTFRMHRLHMRYQ	2345	50	100	1
	FAMKVLASY	FRAYIFAMKVLASYQ	1620	50	100	4
	FQVDISLNL	IAFAFQVDISLNLDQ	1054	50	100	0
	LAMFILNRL	GLAMFILNRLLWNPD	846	50	100	0
	RAYKLYLAV	RAYKLYLAVKNANKQ	3125	50	100	0
	FLNVRASAI	RTRFLNVRASAIIIQ	2996	100	100	0
ATIC	KAFTHTAQY	LKAFTHTAQYDEAIS	176	50	100	1
	YTQSNSVCY	TIAVKYTQSNSVCYA	426	100	100	1
BRD4	FAWPFQQPV	HQFAWPFQQPVDAVK	78	100	100	1
CARS1	ALLENIALY	RPNQALLENIALYLT	541	50	100	22
	YVSNGSVYF	DNGYGYVSNGSVYFD	261	100	100	7
CREBBP	QQMRTLNPL	HQQMRTLNPLGNNPM	515	60	100	0
EP300	LMFNNAWLY	DDIWLMFNNAWLYNR	1122	65	100	1
	YLDSVHFFR	VYISYLDSVHFFRPK	1396	50	100	7
	YSYQNRYHF	DATYYSYQNRYHFCE	1191	50	100	3
ERICH1	FSYWITHIL	AFFSYWITHILPEKS	428	50	100	0
MTOR	HPQALIYPL	HPQALIYPLTVASKS	1967	50	100	1
	LEWLRRLSL	KDDWLEWLRRLSLEL	1280	50	100	1
	LQHYVTMEL	ELQHYVTMELREMSQ	43	50	100	4
	MPFLRKMLI	NPAFVMPFLRKMLIQ	726	50	100	2
	YAMKHFGEL	VLEYAMKHFGELEIQ	1434	55	100	0
	YASRIIHPI	SLDFTDYASRIIHPI	1150	60	100	2
NUP133	RERSSFYSL	RERSSFYSLTSSNIS	280	50	100	0
	YSWDINRAL	EKHAYSWDINRALKE	307	100	100	0
PEG10	QTYPTYAAY	IPGYQTYPTYAAYPT	636	55	100	0
	FMMEMKHVF	PAFMMEMKHVFEDPQ	149	65	100	2
	YAAYPTYPV	YPTYAAYPTYPVGFA	641	50	100	5
	YVAQNGIPL	FIDHEYVAQNGIPLR	381	100	100	2
RRM1	IIYDRDFSY	SAIIYDRDFSYNYFG	134	60	100	8
	RVYNNTARY	LRVYNNTARYVDQGG	276	60	100	14
	FQIVNPHLL	SGEFQIVNPHLLKDL	633	50	100	49
